# The Structural Complexity of the Human *BORIS* Gene in Gametogenesis and Cancer

**DOI:** 10.1371/journal.pone.0013872

**Published:** 2010-11-08

**Authors:** Elena M. Pugacheva, Teruhiko Suzuki, Svetlana D. Pack, Natsuki Kosaka-Suzuki, Jeongheon Yoon, Alexander A. Vostrov, Eugene Barsov, Alexander V. Strunnikov, Herbert C. Morse, Dmitri Loukinov, Victor Lobanenkov

**Affiliations:** 1 Laboratory of Immunopathology, National Institute of Allergy and Infectious Disease, National Institutes of Health (NIH), Rockville, Maryland, United States of America; 2 Chromosome Pathology Unit, Laboratory of Pathology, Center for Cancer Research (CCR), National Cancer Institute (NCI), National Institutes of Health (NIH), Bethesda, Maryland, United States of America; 3 Department of Psychiatry and Behavioral Science, State University of New York at Stony Brook, Stony Brook, New York, United States of America; 4 AIDS and Cancer Viruses Program, SAIC-Frederick/NCI-Frederick, Frederick, Maryland, United States of America; National Institute on Aging, United States of America

## Abstract

**Background:**

*BORIS*/*CTCFL* is a paralogue of *CTCF*, the major epigenetic regulator of vertebrate genomes. BORIS is normally expressed only in germ cells but is aberrantly activated in numerous cancers. While recent studies demonstrated that BORIS is a transcriptional activator of testis-specific genes, little is generally known about its biological and molecular functions.

**Methodology/Principal Findings:**

Here we show that *BORIS* is expressed as 23 isoforms in germline and cancer cells. The isoforms are comprised of alternative N- and C-termini combined with varying numbers of zinc fingers (ZF) in the DNA binding domain. The patterns of *BORIS* isoform expression are distinct in germ and cancer cells. Isoform expression is activated by downregulation of *CTCF*, upregulated by reduction in CpG methylation caused by inactivation of DNMT1 or DNMT3b, and repressed by activation of *p53*. Studies of ectopically expressed isoforms showed that all are translated and localized to the nucleus. Using the testis-specific cerebroside sulfotransferase (*CST)* promoter and the *IGF2/H19* imprinting control region (ICR), it was shown that binding of BORIS isoforms to DNA targets *in vitro* is methylation-sensitive and depends on the number and specific composition of ZF. The ability to bind target DNA and the presence of a specific long amino terminus (N258) in different isoforms are necessary and sufficient to activate *CST* transcription. Comparative sequence analyses revealed an evolutionary burst in mammals with strong conservation of BORIS isoproteins among primates.

**Conclusions:**

The extensive repertoire of spliced *BORIS* variants in humans that confer distinct DNA binding and transcriptional activation properties, and their differential patterns of expression among germ cells and neoplastic cells suggest that the gene is involved in a range of functionally important aspects of both normal gametogenesis and cancer development. In addition, a burst in isoform diversification may be evolutionarily tied to unique aspects of primate speciation.

## Introduction

*BORIS* (Brother Of the Regulator of Imprinted Sites) is a paralog of the multifunctional *CTCF* gene, which is involved in reading epigenetic marks, transcriptional gene activation and repression, X-chromosome inactivation, chromatin loop formation through dimerization and in global three-dimensional genome organization [1], [2], [3], [4], [5]. While the two proteins share a central 11 zinc finger (ZF) DNA binding domain, they have distinct amino- and carboxy-termini [2], [6]. In normal tissues, the two paralogous genes show mutually exclusive expression patterns: *BORIS* mRNA is abundant in male germ cells, particularly in primary spermatocytes and round spermatids, where *CTCF*, which is expressed ubiquitously in somatic cells, is repressed [2]. BORIS acts as transcriptional activator of multiple testis-specific target genes during spermatogenesis, while CTCF suppresses the same targets in somatic cells [7], [8], [9]. In germ cells, BORIS was suggested to be involved in the resetting of imprinting at the *Igf2/H19* imprinting control region (ICR) [10]. In contrast, CTCF is the known reader and protector of *Igf2/H19* imprinting marks in somatic cells [11], [12], [13], [14], [15]. The segregation of *BORIS* and *CTCF* expression in different cell types in mammals is tightly controlled. Normally, CTCF, p53, and CpG methylation suppress *BORIS* transcription in somatic cells, effectively restricting its expression to testicular germ cells [8], [16], where the absence of CTCF [2] and several waves of genome-wide demethylation create the conditions for *BORIS* activation.

*BORIS* is aberrantly activated in many types of cancer cells, its expression coinciding with the loss of CpG methylation, the first epigenetic change identified in cancer cells [2], [6], [17]. Aberrant expression of *BORIS* in cancer cells likely results in a competition between BORIS and CTCF proteins for binding to CTCF DNA binding target sites (CTSes). BORIS can interfere with CTCF functions in cancer cells not just by virtue of having the identical ZF binding domain and overlapping DNA binding specificity, but also due to its distinct amino- and carboxy-termini that likely confer a discrete set of molecular functions [2]. Indeed, both CTCF and BORIS bind the *MAGE A1* promoter, but with opposing results: while CTCF acts as a transcriptional repressor, BORIS functions as an activator [8]. A recent study also demonstrated that BORIS and CTCF perform different transcriptional functions upon binding to the promoter of mouse testis-specific *CST* splice variant [9]. In conclusion, although molecular functions of BORIS in cancer remain to be studied in depth, aberrant co-expression of *CTCF* and *BORIS* is one of the gene expression signatures characteristic of many cancers [6].

Previous studies showed that the evolutionary emergence of *BORIS* in amniotes occurred before the divergence of reptiles and mammals and could be attributed to an initial duplication of the entire *CTCF* sequence [18]. While BORIS is widely expressed in reptiles and monotremes, expression was shown to be gonad-specific in marsupials and eutherians, indicating that BORIS became functionally specialized during mammalian evolution in concert with the evolution of genomic imprinting [18]. While *CTCF* is highly conserved from drosophila to humans [18], [19], [20], *BORIS* coding and noncoding sequences are evolutionarily plastic [18]. Indeed, a comparison of the amino- and carboxy-termini of human *BORIS* with orthologs in other species reveals relatively low similarity, 32.3% and 23.7%, respectively, whereas the corresponding similarity of human *CTCF* with other orthologs is 90.1% and 80.7%, respectively. Nonetheless, the BORIS ZF region has 80.4% identity to its orthologs, similar to the 99.5% conservation for CTCF ZFs [18]. The rapid evolution of *BORIS* is not limited to protein coding sequences. Remarkably, the structure of the locus as a whole is notably different even between mice and humans, which may suggest a specialized set of functions and/or splicing isoforms. The human *BORIS* gene spans over 29 kb at 20q13 and is comprised of 11 exons, 10 of which are coding [2]. This may permit the generation of a number of different isoforms by alternative splicing. Evolutionarily, alternative splicing is the most widely used mechanism for increasing the coding capacity of mRNA transcripts to allow generation of different protein isoforms with distinct activities.

The first evidence for alternative *BORIS* transcripts came from the recent demonstration that human *BORIS* is expressed from at least three alternative promoters utilizing five distinct 5′ UTRs [16]. In the present study, we characterized 23 *BORIS* splice variants with distinct expression profiles in normal germline and cancer cells, while also exhibiting differential DNA binding activities and varying transcriptional properties. Thus, *BORIS* is expressed as a repertoire of alternative transcripts and proteins, indicating that alternative splicing generates a complex mechanism for BORIS-mediated function in germline and cancer cells.

## Results

### Multiple *BORIS* transcripts are expressed in human testis, ES cells and cell lines from different types of cancers

**Figure 1:**
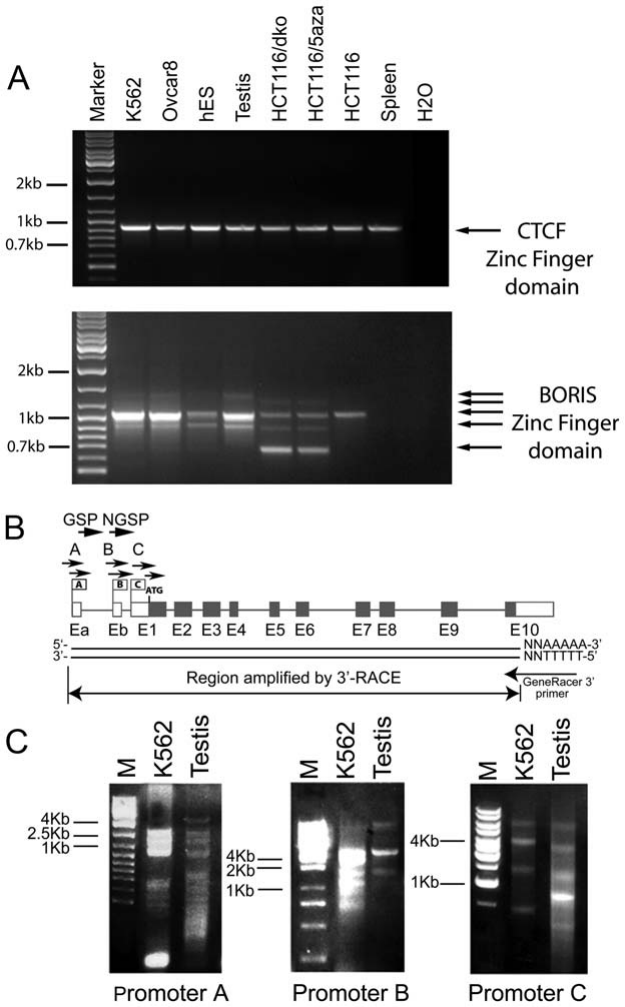
RT-PCR of alternatively spliced *BORIS* transcripts in human testis, ES cells and cancer cell lines. (**A**) Total RNAs from the indicated cell lines and testis tissue were analyzed by RT-PCR using primers designed to amplify the full-length *CTCF* and *BORIS* ZFs domains. The sequences of primers are shown in Table S1. Nested PCR was performed with 20 and 35 cycles for first and second rounds, respectively. The sources of RNA are shown on top of each gel. Arrows point to the single *CTCF* transcript and multiple *BORIS* alternative transcripts. (**B**) 3′ RLM-RACE strategy for cloning alternatively spliced *BORIS* forms. Three *BORIS* alternative promoters and 12 exons with noncoding sequences (white boxes) or coding sequences (grey boxes) are shown. Total RNA from adult human testis and the K562 cell line was processed with GeneRacer PCR kit for the amplification of full-length cDNAs. The first round of PCR was performed with three forward gene-specific (GSP) primers A, B, and C that were designed to identify mRNA expressed from corresponding alternative *BORIS* promoters A, B or C, respectively. The 3′ GeneRacer primer attached to the poly A tail was used as a reverse primer. l µl of the first-round PCR mixture was used as a template to perform nested PCR with three forward nested gene-specific (NGSP) primers A1, B1, and C1. (**C**) 3′ RLM-RACE was performed on human testis and the K562 cell line. Multiple *BORIS* transcripts were detected in both samples. PCR products of the nested PCR are shown separated on 1% agarose gels. M is the size marker.

We previously demonstrated that expression of *BORIS* is restricted to testis tissues and cancer cells, with expression dependent on the CpG methylation status of alternative *BORIS* promoters [8], [16]. In analyzing *CTCF* and *BORIS* expression in human testis, embryonic stem (ES) cells, and several cancer cell lines by RT-PCR, we were able to amplify the full-length ZF-encoding regions of both genes. Only a single PCR band specific for the *CTCF* ZF domain was detected in all cell types examined; however, multiple PCR bands were generated by primers amplifying the *BORIS* region corresponding to ZF domain (Fig. 1A). Sequence analyses of cloned *BORIS* PCR products identified a number of alternative transcripts with different combinations of ZF exons that were generated due to the utilization of alternative splice sites (Fig. 1A). The abundance and distribution of *BORIS* transcript variants differed among cancer cell lines and in testis, suggesting distinct mechanisms of regulation of *BORIS* gene in normal germline and cancer cells.

Knowing that CpG hypomethylation is involved in *BORIS* activation [2], [8], [16], we compared *BORIS* expression in the HCT116 colon cancer cell line to HCT116 cells treated with 5aza-dC, and to HCT116 bearing a double knockout (DKO) of *DNMT3b* and *DNMT1* [21]. A dramatic decrease in CpG methylation in HCT116 DKO and HCT116 cells treated with 5aza-dC, described previously [21], correlated with the appearance of multiple *BORIS*-specific RT-PCR products (Fig. 1A), which were absent in the parental HCT116 cell line. Interestingly, human ES cells were found to express at least two alternative *BORIS* transcripts, confirming *BORIS* expression in ES cell lines as demonstrated previously by immunofluorescence [22]. We therefore conclude that while *CTCF* is expressed as a single transcript in human testis and cancers, *BORIS* is expressed as multiple isoforms in the testis, ES cells and in cancer cell lines, specifically in cells with increased DNA hypomethylation.

### Twenty three alternatively spliced *BORIS* isoforms are expressed in testis and in cancer cells

**Figure 2:**
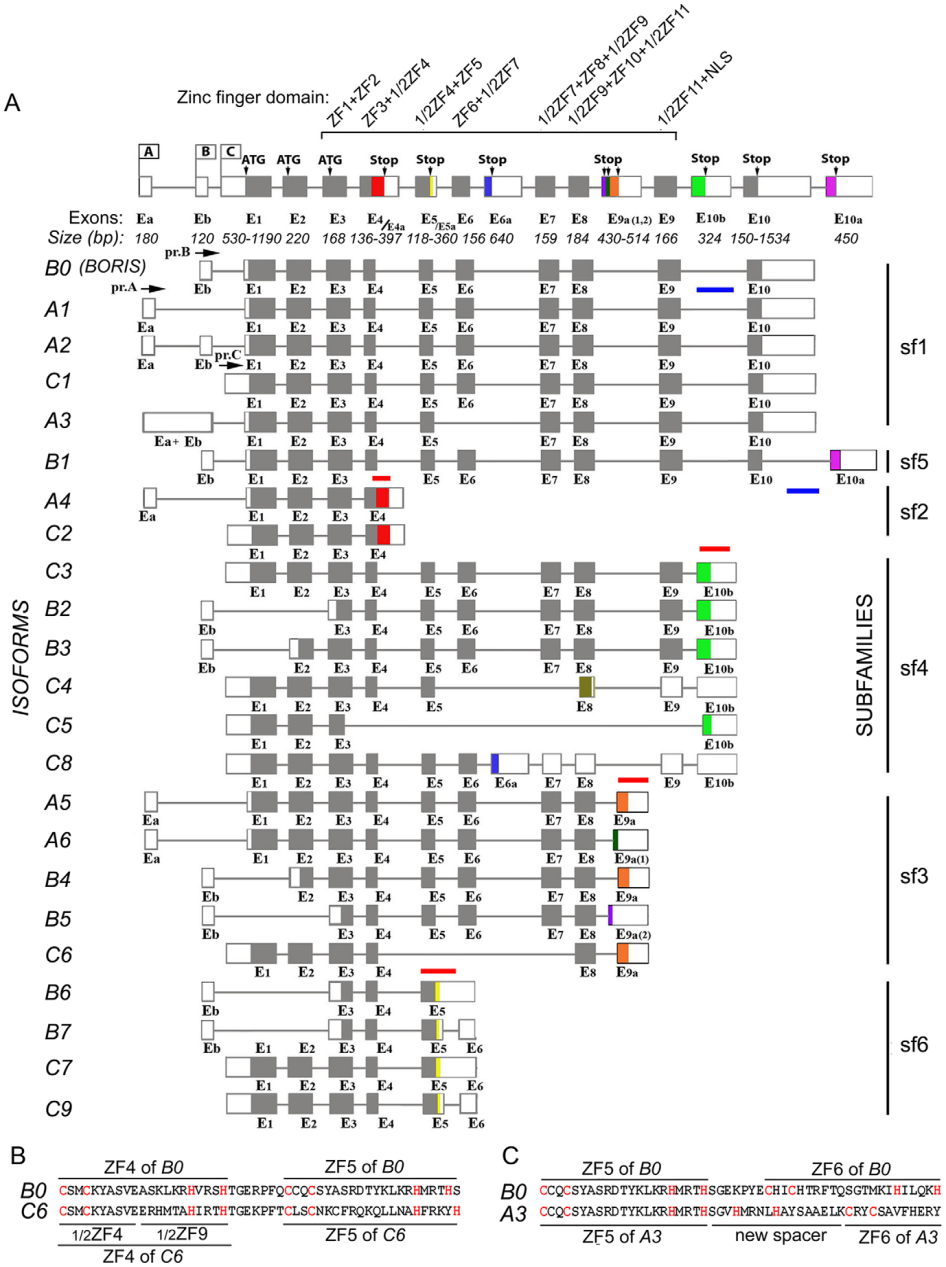
Organization of the human *BORIS* gene with alternative *BORIS* transcripts. (**A**) Schematic illustration of the *BORIS* gene with three alternative promoters and sixteen exons used for generation of 23 isoform mRNAs. Exons sizes are shown under the scheme, with minimum and maximum number of nucleotides for the alternative exons. The start and stop codons for ORFs are indicated as ATG and Stop, respectively. The first *BORIS* transcript represents the originally cloned *BORIS* form, designated here as *B0*; the *BORIS* transcripts shown below are novel cloned isoforms. On the left are the names of *BORIS* isoforms, corresponding to schematic representation of given transcripts. On the right, the six *BORIS* subfamilies, divided on the basis of similar 3′ ends, are indicated. The red and blue lines on the top or bottom of the schematic representation of isoforms depict the locations of Taqman probes. Untranslated regions are represented by open boxes. Introns (thin lines) are not shown to exact scale. (**B**) Alternative splicing creates a new ZF in the *C6* isoform. Amino acid sequence comparisons of ZF 4 of *B0* and new alternative ZF 4/9 of *C6,* which combines the first half of ZF 4 and the second half of ZF 9. (**C**) Alternative splicing creates a new coding spacer between ZFs 5 and 6 in *A3* isoform.

Prompted by our previous identification of five alternative 5′-UTR splicing *BORIS* variants generated from three alternative *BORIS* promoters (A, B and C) [16], we conducted a screen for full-length alternatively spliced *BORIS* transcripts in human testis and the K562 cancer cell line, the cells with the highest levels of *BORIS* expression. To isolate full-length *BORIS* alternative transcripts, we utilized the 3′-RLM-RACE approach shown in Figure 1B. We amplified multiple *BORIS* RT-PCR products that were then cloned and sequenced (Fig. 1C). From this, we identified 19 previously unknown *BORIS* splice variants (Fig. 2). The two main sources of heterogeneity in *BORIS* mRNAs were the usage of alternative promoters and splice sites. We also observed differential usage of distinct 5′- and 3′-UTRs as well as alternative translation frames in the amino- and carboxy-terminal coding regions. Alignment of the *BORIS* genomic sequence with alternative transcripts revealed that exon-intron boundaries had classic splice site sequences (Table S4). Most of the alternative *BORIS* transcripts possessed a polyA tail located 20–30 bp downstream from the canonical polyadenylation signal, AAUAAA (File S1), indicating that *BORIS* isoforms reached the stage of expression as mature mRNAs.

The *BORIS* isoforms were categorized according to promoter usage (Fig. 2A). Isoforms driven from promoter A included *BORIS A1*, *A2*, *A3, A4, A5*, and *A6*. Compared to the originally described *BORIS* transcript [2], which is now designated as the *BORIS B0* isoform, isoforms *A1* and *A2* contained alternative 5′ UTRs, but encoded the same BORIS polypeptide (Fig. 2A, Table S3). Isoform *A3* had several new features that distinguished it from *B0* including a long non-coding 5′ UTR and the exclusion of exon 6 due to alternative splicing. This resulted in the presence of only 9 ZFs, rather than the 11 of *BORIS B0*, but also produced a new long spacer between ZF5 and ZF8 (Fig. 2A, C). For isoforms *A4* and *C2*, which both encode the same polypeptide, ORFs continue into intron 4 until an alternative stop codon resulted in the truncation of the ZF domain while producing an alternative COOH-terminus. Isoforms *A5* and *A6* both encoded 10 full ZFs and half of ZF11, which is alternatively spliced from exon 8 to new exons 9a or 9a(1), respectively, resulting in two alternative carboxy-termini. The isoform *B1* has the same 10 coding exons as the *BORIS B0* prototype, but possessed an additional exon 11, which encoded an alternative carboxy-terminus. In addition, some isoforms had alternative amino-termini due to the utilization of different start codons, which result from alternative splicing of exon Eb to exon 2 (in *B3* and *B4*) or to exon 3 (in *B2, B5, B6, B7*).

Most surprisingly, only 7 out of 23 *BORIS* isoforms encoded a full-length 11 ZF DNA binding domain, with the number of ZF in the other isoforms ranging from 1 to 10 (File S1, Table S3). As shown by the following examples, reductions in the numbers of ZFs resulted from the utilization of alternative splice sites and stop codons. The isoform *C5* had only one ZF and an alternative carboxy-terminus resulting from splicing from the middle of exon 3 to exon 10b. While isoform *C8* contained all 11 exons, the presence of an additional exon 6a with an in-frame stop codon resulted in just six ZFs. Remarkably, splicing from exon 4 to exon 8 in isoform *C6* created a new hybrid ZF comprised of half of ZF 4 and half of ZF 9. This raises the possibility that the new ZF could confer new DNA binding properties to this isoform (Fig. 2B). Finally, some alternative exons, such as 5a in *B6, B7, C7*, and *C9*, retained intronic sequences that incorporated premature stop codons, and therefore may not produce stable protein due to the nonsense-mediated mRNA decay (NMD) pathway, a possibility that should be verified experimentally.

### Characteristic features of *BORIS* alternative variants and their evolutionary conservation in humans and non-human primates

The 23 *BORIS* mRNA splice variants have the potential to encode 17 different polypeptides that we designate as BORIS isoform proteins 1 through 17. To categorize the isoforms, the alternative amino- and carboxy-termini were named according to the number of amino acid residues upstream and downstream of the ZF domain, respectively (Table S3). For example, N258 denotes an amino-terminus with 258 amino acid residues upstream of the ZF domain that is encoded in many *BORIS* isoforms including *B0, B1, A3, A4, A5, A6, C3, C4, C5, C6, C7/C9* and *C8*. Notably, N24 and N53, truncated versions of N258, have no amino acid differences with N258 within the 24 and 53 amino acids upstream from ZF1. Eleven alternative carboxy-termini found in distinct isoforms are designated as “C”, with their numbers corresponding to the number of codons downstream of the last ZF. For example, *B1* has C132, *C3* has C97, *C5* has C53, etc. (File S1, Table S3).

To search for homology with known proteins or domains, the eleven unique alternative C-termini were compared by BLAST to GenBank amino acid sequences. Only one alternative carboxy-terminus, C97, showed a significant degree of similarity with multiple non-BORIS proteins, including several involved in the processes of transcription or translation (Fig. S2). This newly recognized putative domain is a previously uncharacterized component of a known helicase-like domain (COG0553). Further analyses of alternative 3′UTRs of some isoforms revealed the presence of specific repetitive DNA elements. For example, a part of the 3′UTR for the *B6* and *C7* isoforms belongs to an Alu-J consensus. The 3′UTR of isoform *B1* is also highly repetitive in human and primate genomes. Isoforms *C3, B2, B3, C4, C5*, and *C8* have the primate-specific repetitive DNA element, MER1 [23] in their 3′UTRs.

**Figure 3:**
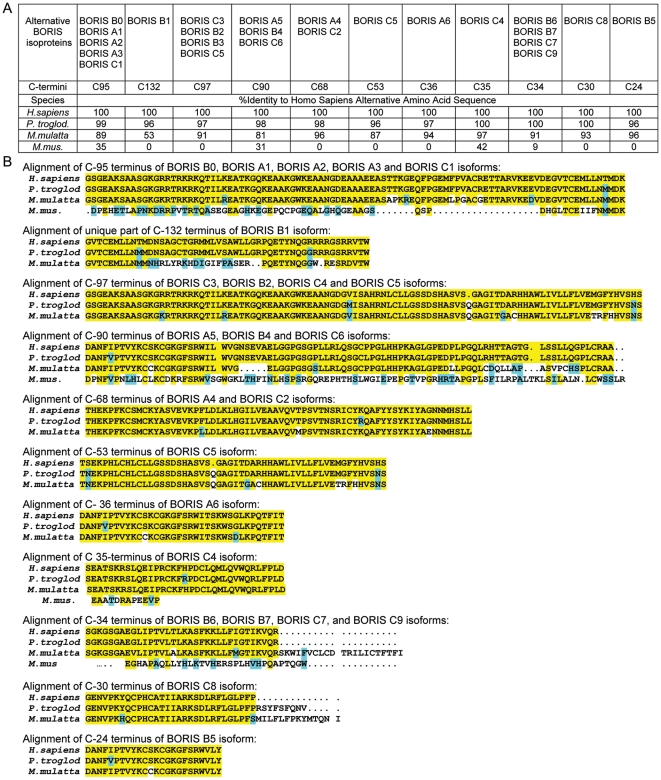
Human BORIS isoform amino acid sequences are evolutionarily conserved in primates. (**A**) Percent identity of human (*H.sapiens*) BORIS isoforms C-termini to putative alternative amino acid sequences those of chimpanzee (*P.troglod.*), macaque (*M.mulatta*), and mouse (*M.mus*.). Eleven alternative C-termini found in distinct isoforms are defined by the number of codons downstream of the last ZF. The table shows names of BORIS isoforms with corresponding C-termini and their identity in percent. (**B**) Alignment of BORIS alternative C-termini in human, chimpanzee, macaque and mouse. The alternative amino acid sequences of human, chimpanzee, macaque and mouse are aligned by ClustalW (Vector NTI). Human BORIS splice variants are highly conserved in primates, but not in mice. Amino acid sequences for some of BORIS alternative C-termini (C132, C97, C68, C53, C36, C30, C24) are completely absent in mice. Yellow highlighted sequences are 100% identical to the human amino acid sequence; the blue highlight indicates conservative substitutions relative to human homologous BORIS sequences. Dots indicate insertions or deletions.

The fact that *BORIS* isoforms are conserved in other species suggests their biological significance. For example, all features of human *BORIS* isoforms are highly conserved in the apes *Pan troglodytes* and *Macaca mullata,* with conserved splice sites and corresponding protein identities ranging from 96% to 100% and from 53% to 97%, respectively (Fig. 3). Among the most conserved C-termini are: C95 (with identities of 99% and 89% in chimpanzee and macaques, respectively), C97 (97% and 91%), C68 (98% and 96%), C35 (100% and 97%), and C24 (96% and 96%). C132 is highly conserved in the chimpanzee (96%), but less so in macaques (53%). While alignment of human *BORIS* isoforms with the mouse genomic locus uncovered several putative mouse BORIS carboxy-termini (C95, C90, C35, and C34), the homology levels were rather low, ranging from 9% to 42%. This suggests that the pace of *BORIS* evolution in mammals has been quite rapid and the complexity of *BORIS* locus likely coincided with the emergence of primates. The fact that the mouse *Boris* locus does not have the same range of isoforms as humans or other primates may be related to primate-specific evolution of intron sequences of *BORIS* loci [18]. It remains to be determined whether mice have alternative *Boris* isoform species. If they exist, it should be anticipated that they would be distinct from those of humans despite the conservation of splice sites. The emergence of isoforms in primates may thus be attributed to putative intronic splicing enhancers.

### Alternative *BORIS* transcripts are expressed in normal male and female gonads

**Figure 4:**
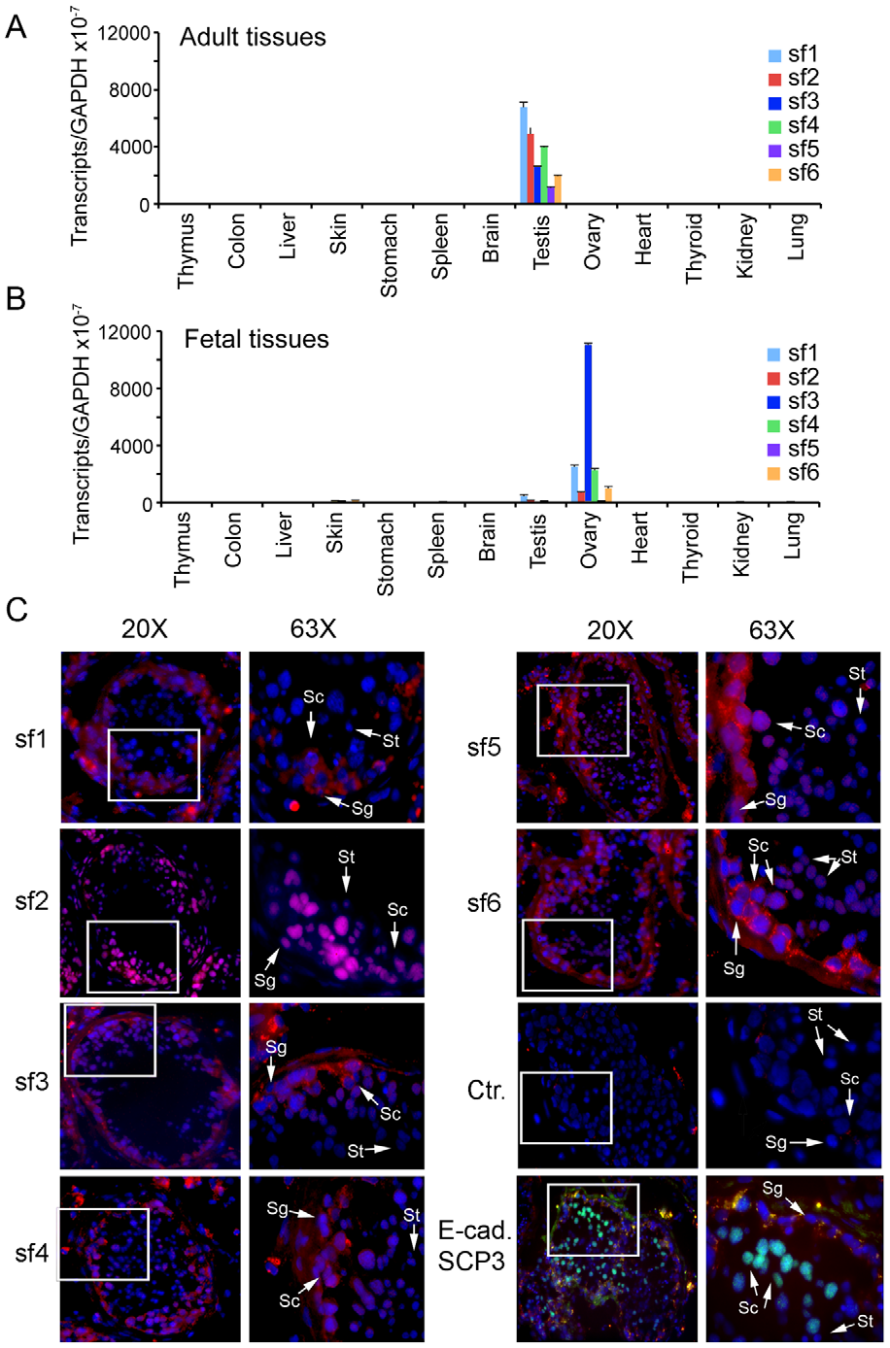
Expression of *BORIS* alternative transcripts in male and female gonads. qRT-PCR analysis of *BORIS* isoform expression in normal human adult (**A**) and fetal (**B**) tissues, respectively, was quantified by the absolute quantification approach. The 23 *BORIS* isoforms were divided into 6 subfamilies based on their unique 3′ end sequences to enable the design of Taqman probes (Material and Methods). (**C**) *BORIS* isoform expression in human normal adult testis analyzed by *in situ* hybridization, RNA FISH. For FISH assays, the probes for six *BORIS* subfamilies (sf1–sf6) were labeled with digoxygenin-11-dUTP by PCR and individually hybridized to formaldehyde-fixed adult human testis. The slides were incubated with anti-DIG antibodies overnight and then visualized with rhodamine-conjugated secondary antibody. To identify spermatogonia and spermatocytes, we performed immunostaining with antibodies against SCP3 (red) and E-cadherin (light green), respectively. Merged images of DAPI-stained nuclei (blue) and *BORIS* isoform RNA (red) taken with 20X magnification; higher magnification images are 63X. Arrows with letters indicate: Sg-spermatogonia, Sc – spermatocytes, St – spermatids (small, elongated cells with elongated nuclei, respectively). The control (Ctr) is staining with rhodamine-conjugated secondary antibody alone.

We previously reported that expression of *BORIS B0* in normal human tissues was restricted to testis [2]. To analyze the patterns of *BORIS* isoform expression in testis, we designed a series of primers and Taqman probes to amplify the alternative transcripts by qRT-PCR. Only 8 of 23 *BORIS* isoforms could be specifically discriminated by qRT-PCR because most isoforms share sequences, making it impossible to design primers and probes that would detect every *BORIS* isoform as a separate species. Consequently, we operationally divided the 23 isoforms into six subfamilies (sf1 to sf6) based on their unique 3′ terminal sequences, which were used to design 6 Taqman probes for qRT-PCR (Fig. 2A, Materials and Methods). Among 13 adult and 13 fetal tissues tested, expression of the six *BORIS* subfamilies was detected only in adult testis and in embryonic ovary, but the relative levels of isoform expression were reproducibly different in the two tissues (Fig. 4A,B). In adult testis, all six subfamilies were expressed at comparable levels, with sf1 being the most prevalent group, expressed at approximately 1.3- to 3-fold higher levels than the other five subfamilies (Fig. 4A). In contrast, sf3 was the most prevalent form in embryonic ovaries (Fig. 4B), being expressed at levels approximately 4-fold higher than sf1 and sf4, and 11- to 127-fold higher than the sf6, sf2, and sf5 groups (Fig. 4B). While among adult tissues only testis was strongly positive for *BORIS* isoforms, they were expressed at very low albeit reproducible levels in several tissues in the fetal panel, including testis, skin, and spleen (Fig. 4B). This suggests that *BORIS* isoforms may be functionally active outside the germline during fetal development.

To identify the specific cell types that express *BORIS* isoforms in adult testis, we carried out RNA *in situ* hybridization using fixed preparations of normal human testis. Immunostaining of testis with antibodies to SCP3 and E-cadherin was used to distinguish spermatogonia and spermatocytes, respectively, while spermatids were identified morphologically as small and elongated cells with elongated nuclei (Fig. 4C, E-cad, SCP3). Following hybridization to six labeled PCR probes designed to specifically detect each of the six *BORIS* subfamilies, isoform transcripts were detected at nearly all stages of spermatogenesis (Fig. 4C). All six *BORIS* transcript subfamilies were less abundant in the cytoplasm of spermatogonia than spermatocytes, while spermatids were highly positive for *BORIS* sf2 and marginally positive for sf5 and sf6. Thus, sf2, sf5 and sf6 appear to characterize the later stages of spermatogenesis from spermatocytes to spermatids. A previous study [2] using chicken anti-BORIS antibody and a 5′ end-labeled *BORIS* probe, both specific for the N258 terminus, showed that expression of the *BORIS B0* isoform was restricted primarily to spermatocytes. The present work complements our previous assessment of *BORIS* transcript localization by providing evidence for the expression of all six *BORIS* subfamilies in adult human testis. The apparent differential expression of individual subfamilies during the progression from early to later stages of spermatogenesis indicates that expression of *BORIS* isoforms is developmentally regulated.

### *BORIS* isoforms encode putative cancer-testis antigens

**Figure 5:**
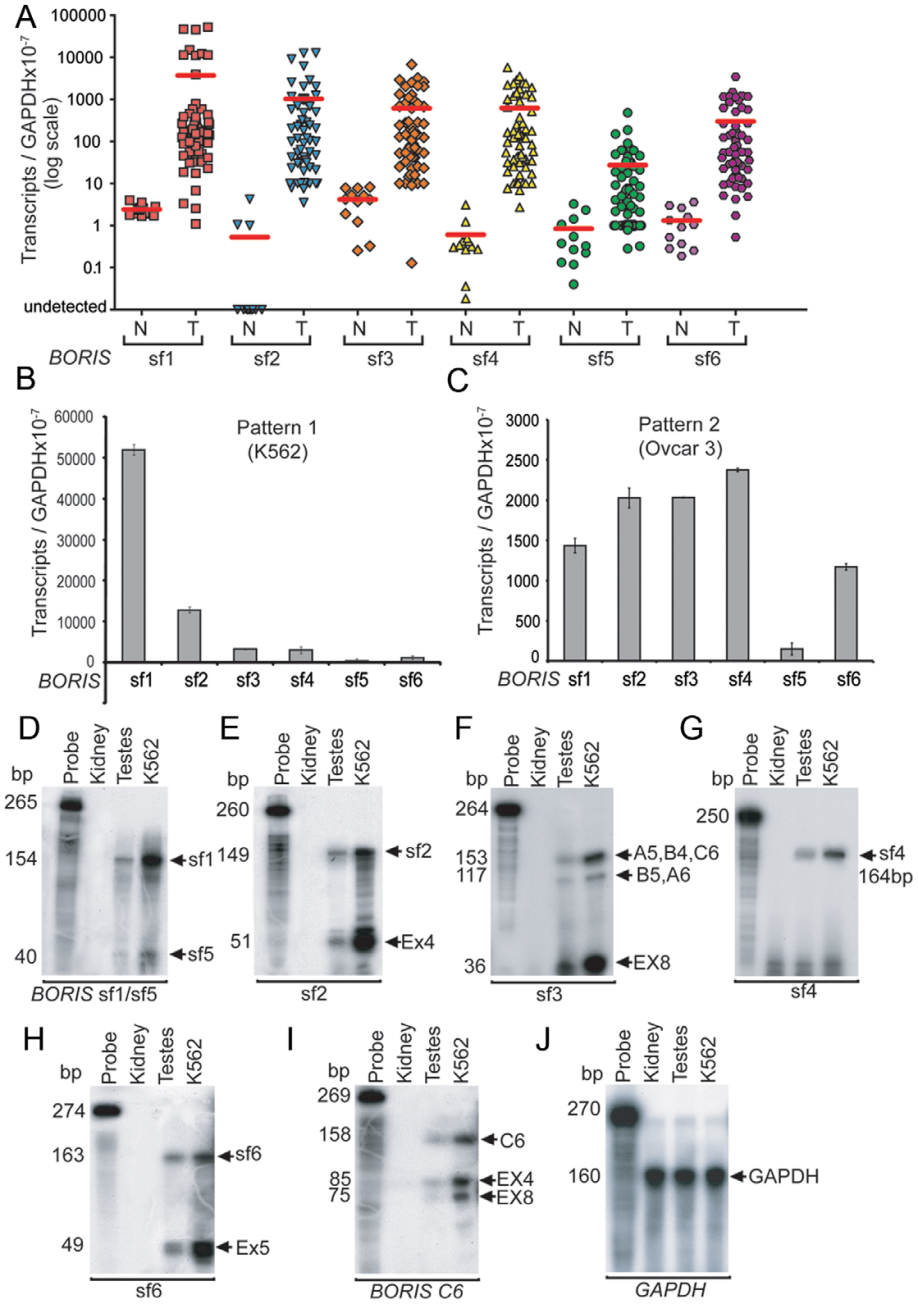
Expression of *BORIS* isoforms in cancer cell lines. (**A**) The *BORIS* subfamilies are significantly upregulated in cancer cells as compared to normal cells. For normal cells (N) – the expression of *BORIS* isoforms was analysed in 13 tissues and 4 primary cell lines. For tumor cells (T) - the NCI-60 cancer cell lines were analyzed. 59 of the NCI-60 cancer cell lines, 13 normal tissues, and 4 normal primary cell lines were analyzed by the absolute quantification approach with normalization to GAPDH levels, and plotted using a logarithmic scale. Levels of *BORIS* isoform expression vary greatly (from 0 to 60000 transcripts per 50 ng of total RNA). Red lines indicate mean values for each *BORIS* subfamily. (**B**) The first pattern of *BORIS* isoform expression in NCI-60 cancer cells, as represented by the K562 cancer cell line. (**C**) The second pattern of *BORIS* isoforms expression in NCI-60 panel is exemplified by the Ovcar3 cancer cell line. (**D-J**) *BORIS* isoforms expression analyzed by RPA. Aliquots of total RNA obtained from kidney, testis and K562 cell line were hybridized with ^32^P–labeled antisense RNA probes. The RNase protected fragments (arrowheads) were detected only in testis and K562, but not in kidney. The first lane on each gel is a control, an undigested probe incubated with yeast RNA. (**D**) RPA with riboprobe sf1/sf5 yields 154 bp and 40 bp fragments, corresponding to sf1 and sf5, respectively. (**E**) The sf2 probe protects 149 bp of sf2 and 51 bp of exon 4. (**F**) The sf3 riboprobe protects 153 and 117 bp of sf3. (**G**) The sf4 riboprobe protects 164 bp sequence of sf4. (**H**) The sf6 riboprobe yields 163 bp of sf6 and 49 bp of exon 5. (**I**) The C6 riboprobe demonstrates alternative (158 bp) splicing in ZF region. (**J**) GAPDH probe was used as a loading control. This experiment was repeated twice with consistent results.

Previous studies showed that the *BORIS B0* isoform is abnormally expressed in many types of human cancers, including both primary cancers and cancer cell lines, qualifying it as encoding a putative cancer-testis antigen (CTA) [6], [8], [24], [25], [26]. To understand the relevance of the newly discovered multiple *BORIS* isoforms to cancer development and progression, we tested the NCI-60 cancer cell line panel by RT-PCR and found that about 70% of those cell lines expressed transcripts of some isoforms (Fig. 5A). Most of the positive lines, however, expressed levels of *BORIS* transcripts that were quite low at less than 500–1,500 transcripts per 50 ng of total RNA. Nevertheless, the levels were sufficient to detect two distinct patterns of *BORIS* isoform expression. The first pattern, exemplified in Figure 5B for the K562 cell line, was associated with more than 20,000 *BORIS* transcripts (summed over all six *BORIS* subfamilies) per 50 ng of total RNA. In this subset of cell lines (8 out of 60, 13% of the NCI-60 panel), sf1 transcripts were present at the highest levels, averaging about 3-fold higher than levels for sf2, 10-fold higher than for sf3 and sf4, 50-fold higher than for sf6, and more than 100-fold higher than for sf5 (Fig. 5B). The cell lines showing this expression pattern originated from different tissues, including ovary, lung, breast, blood, and skin, indicating that expression of *BORIS* isoforms is not specifically associated with cancers of particular origins.

More than 50% of the positive cell lines from the NCI-60 panel exhibited a second pattern of *BORIS* isoform expression. This pattern, exemplified by the Ovcar 3 cell line (Fig. 5C), was characterized by less than 20,000 summed *BORIS* transcripts per 50 ng of total RNA. Unlike the first pattern, five of the six subfamilies were expressed at approximately equal levels, while sf5 showed notably lower level of expression (Fig. 5C). Furthermore, the pattern of differential expression is even more complex, as some *BORIS* isoforms belonging to a single family were not expressed equally, with some more abundant than the others. This enabled us to identify the dominant *BORIS* isoforms for all six subfamilies (File S2, Fig. S4).

To confirm the expression of *BORIS* isoforms in cancer cell lines and testis by independent means, we used both northern blotting (File S2, Fig. S4) and Ribonuclease Protection Assay (RPA) (Fig. 5D–J). Both are extremely specific methods for detecting and quantifying RNA species. For RPA, five riboprobes spanning the unique regions of *BORIS* subfamilies were used, along with one riboprobe to detect the rearrangement of zinc fingers in the ZF binding domain of isoform *C6*. The protected bands in RPA resulted from the hybridization of riboprobes to total RNAs from either testis or the K562 cell line; all six probes were fully digested when incubated with kidney RNA(Fig. 5D–J). These results confirm the Real-Time PCR data and the assignment of *BORIS* isoforms to the CTA family. The sizes of fully protected major bands correspond to sf1 (154 bp), sf2 (149 bp), sf3 (153 bp), sf4 (146 bp), sf5 (40 bp), sf6 (163 bp), and C6 (158 bp) (Fig. 5D–J). Furthermore, due to the overlapping sequences among *BORIS* subfamilies, several smaller bands corresponding to the protected fragments of other subfamilies/isoforms were present on one gel. For example, the riboprobe sf1/sf5 (Fig. 5D), which spans coding and noncoding sequences of exon 10, protected two bands of 154 bp and 40 bp, indicating the transcrption of sf1 and sf5 transcripts, respectively, in both testis and the K562 cell line. Similarly, the sf2 probe protected 149 bp of sf2 and 51 bp of exon 4 coding sequence that presents in most *BORIS* subfamilies, documenting the relatively equal and low expression, compared to other subfamilies, of sf2 in testis and the K562 cell line, respectively, (Fig. 5E). The sf3 riboprobe specifically protected 153 bp of the *A5, B4*, and *C6* isoforms of sf3, 117 bp of the *B5* and *A6* isoforms of sf3, and 36 bp of exon 8, revealing relatively low expression of sf3 when compared to the total transcripts for sf1, sf4 and sf5 (Fig. 5F). The sf4 probe detected only subfamily 4, resulting in a single protected fragment of expected size (Fig. 5G). The riboprobe sf6 yielded 163 bp of sf6 and 46 bp of exon5, present in most *BORIS* isoforms (Fig. 5H) The C6 riboprobe detected alternative (185 bp) and originally-described (85 bp and 75 bp) splicing in ZF region of *BORIS* (Fig. 5I). Thus, the results from RPA analyses of *BORIS* subfamily expression were in close agreement with Real-Time PCR data, demonstrating the dominant expression of sf1 in K562 and relatively equivalent expression of *BORIS* subfamilies in testis.

An additional search for *BORIS*-related expressed sequence tags (ESTs) in DNA sequence databases revealed that *BORIS* sf2 is expressed in primary and metastatic Wilms' tumors (Table S5). Moreover, *BORIS* sf1 was found to be expressed in many types of primary cancers, including retinoblastoma, chronic myelogenous leukemia, and mucoepidermoid carcinoma (Table S5). Thus, *BORIS* isoforms were aberrantly activated not only in established cancer cell lines, but also in primary cancers of different origins.

### The 23 alternatively spliced mRNA variants encode 17 alternative nuclear BORIS proteins

**Figure 6:**
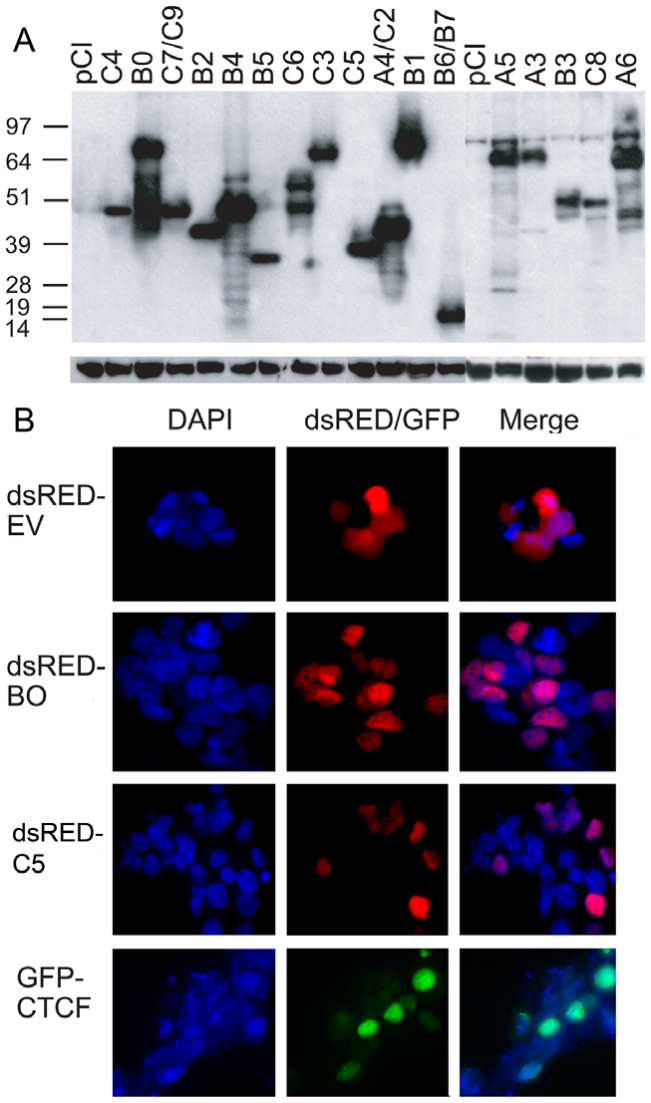
BORIS isoforms are translated into proteins with nuclear localization *in vivo*. (**A**) Immunoblotting detection of ectopically expressed BORIS isoform proteins. Expression vectors with HA-Tag fused BORIS isoforms were transiently transfected into HEK293T cell line. The transfection efficiency, monitored by co-transfection with a GFP-expressing plasmid, was similar for all transfected plasmids. Total protein was extracted, separated on a 12% SDS-PAGE gel, and probed with rat monoclonal anti-HA antibody (Roche). Molecular mass standards are on the left. The sizes (in kDa) of BORIS isoform proteins correspond to thier predicted molecular masses, as shown in Table S3. (**B**) Nuclear localization of CTCF, B0, and C5 isoproteins. The localization of the rest BORIS isoproteins is shown in Fig. S3. HEK293T cells transiently transfected with either *BORIS* or *CTCF* fused at the N-terminus to *dsRFP* or *GFP*, respectively, were analyzed for RFP and GFP fluorescence by microscopy. dsRED protein (Empty Vector (EV)-dsRED) served as a marker for cytoplasmic location. Cells were also stained with DAPI to visualize nuclear DNA. Both BORIS and CTCF were detected in the nuclei.

To determine whether BORIS isoforms are translated and to examine their subcellular localization, 17 ORFs corresponding to 17 alternative BORIS proteins (Table S3) were cloned as amino-terminal HA-tag fusions into mammalian expression constructs based on the pCI vector. Following transient transfection into HEK293T cells, immunoblotting with anti-HA-Tag antibodies showed that all *BORIS* isoforms, when expressed ectopically, were translated into proteins of expected molecular mass (Fig. 6A). Some BORIS isoform proteins, refered to as isoproteins here, such as B0, B1, A6, A5, C3, and B4, were expressed more efficiently than others, indicating enhanced stability of these proteins.

BORIS protein, identified by an antibody that could potentially detect multiple BORIS isoforms, was previously reported to localize to both the nucleus and the cytoplasm of primary spermatocytes [2]. This prompted us to characterize the subcellular localization of individual BORIS isoproteins. HEK293T cells, primary normal human dermal fibroblasts (NHDF), and the colon cancer cell line, HCT-15, were transiently transfected with expression constructs bearing fusions of BORIS isoform ORFs with the coding region of the modified red fluorescent protein, DsRed. The localization of BORIS isoforms was restricted to the nucleus in all three cell types at 24 h and 48 h after transfection (Fig. 6B and Fig. S3). Control transfections with a DsRed-expressing vector demonstrated uniform expression of RFP throughout the cell, while CTCF fused to GFP localized exclusively to the nucleus. Under higher magnification, multiple red fluorescent spots were detectable inside the nucleus for all BORIS isoforms (Fig. 6B and Fig. S3). The patterns of BORIS isoform expression in the nucleus were thus very similar to the punctate pattern described previously [22], [27]. The nuclear localization of BORIS isoproteins is consistent with the idea that they bind DNA and/or regulate some processes in chromatin.

### A subset of BORIS isoform proteins binds the human *H19* ICR in a methylation-sensitive manner

**Figure 7:**
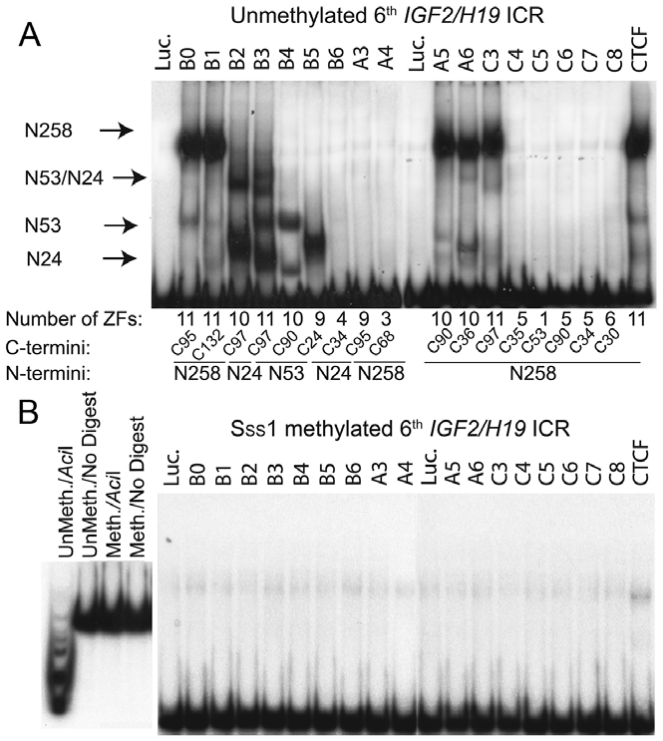
BORIS isoforms bind to the human *IGF2/H19* ICR in a methylation-sensitive manner. (**A**) Binding of *in vitro* translated BORIS isoforms to the sixth CTCF target site in the human *H19* ICR by EMSA. *In vitro* translated CTCF and luciferase proteins were used as positive and negative controls, respectively. The DNA-protein shifts corresponding to the particular N- termini are indicated by arrows. The number of ZFs and alternative N-and C-termini for each isoform are indicated at the bottom of gel. (**B**) DNA binding of BORIS isoforms to the *H19* ICR is methylation-sensitive. EMSA of BORIS isoform binding performed with *Sss*I-methylated *H19* ICR probe shows that none of 17 BORIS isoforms bound to the methylated *H19* ICR. The left panel shows the quality control of the methylated probe by digestion with methylation-sensitive restriction endonuclease *Aci*I of ^32^P-labeled methylated and unmethylated *H19* ICR probes.

Since many BORIS isoproteins possess a ZF DNA binding domain that is highly similar to that of CTCF, their expression in the same cell could interfere with the binding of CTCF to its targets. To investigate the ability of BORIS isoforms to bind to CTCF target sites, we conducted EMSA with the sixth CTS of the *IGF2/H19* ICR, which has been shown to correlate with monoallelic expression of *IGF2* and *H19* [28]. As shown in Figure 7A, nine of seventeen *in vitro* translated BORIS isoproteins (B1, B2, B3, B4, B5, A5, A6, C3) and full length CTCF as a control, bound DNA, evidenced by the specific retardation of the labeled *H19* ICR probe. The negative control, luciferase, as well as the remaining eight BORIS isoforms (B6, A3, A4, C4, C5, C6, C7, C8) were inactive in EMSA with this probe.

These findings indicate that the first nine ZFs of the BORIS DNA binding domain are required for binding to the sixth CTCF target site in the *IGF2/H19* ICR *in vitro.* The alternative amino- and carboxy-termini apparently have less prominent roles in the DNA binding activities of these isoforms. However, the particular gel migration shifts of BORIS-DNA complexes appeared to be specific for the individual amino- and carboxy-termini. For example, isoproteins B0, B1, A5, A6 and C3, all having the N258 terminus, produced one dominant, highly shifted band, comparable to the shift seen with full-length CTCF (Fig. 7A). At the same time, the truncated version of the N258 terminus found in isoproteins B2, B3, B4, and B5 was associated with multiple much lower band-shifts that may also be attributable to different alternative carboxy-termini. Notably, two of the four band-shifts of isoprotein B3, having the N53 terminus, were similar to two of those of B4, while the two higher band-shifts were observed only for B3. This difference between the B3 and B4 isoproteins is likely conferred by two distinct carboxy-termini, C97 and C90, respectively, indicating that C97 is responsible for two additional EMSA bands and probably for a substantially different DNA-BORIS complex. This view is supported by analyses of the binding of isoproteins B2 and B5 to DNA, as B2, which has C97, resulted in a band shift similar to B3 (Fig. 7A). Thus, both amino- and carboxy-termini appear to influence the migration and, putatively, the structure of BORIS-DNA complexes, which may lead to different functions of BORIS isoforms bound to the same target.To evaluate the effect of DNA methylation on the binding activities of BORIS isoforms, we performed EMSAs using a *H19* ICR probe with CpGs methylated *in vitro* by *Sss*I methyltransferase. As shown in Figure 7B, binding of CTCF and all BORIS isoforms to the methylated ICR was completely abrogated by CpG methylation. This finding was highly reproducible and contrasts with an earlier study that reported methylation-independent binding of BORIS to the *IGF2/H19* ICR [29]. Thus, binding of BORIS isoproteins to DNA, at least to the *H19* ICR probe, is methylation-sensitive, as shown previously for CTCF. These results suggest that CTCF and BORIS isoproteins may be functionally relevant competitors for target sites *in vivo* as well as lend support to the proposed role for BORIS in the regulation of imprinting in primary spermatocytes and round spermatids where CTCF is not expressed [2].

### BORIS proteins bind to the testis-specific promoters of human and mouse *CST* genes

**Figure 8:**
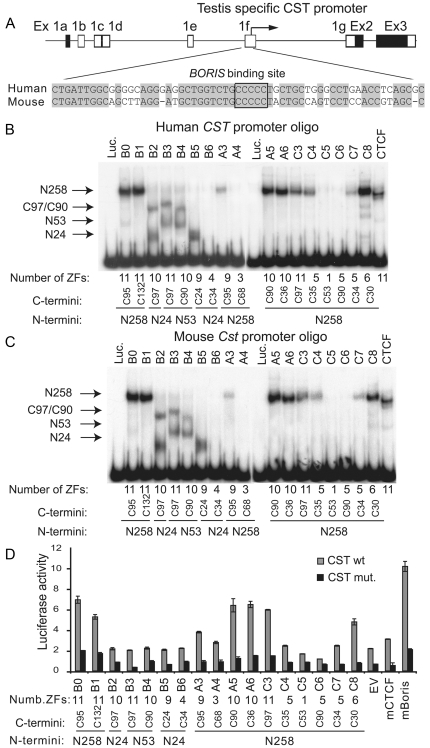
BORIS isoforms bind and activate the transcription of conserved human and mouse *CST* testis-specific promoter regions. (**A**) Schematic representation of multiple mouse *CST* promoters, where exon 1f_s_ has been shown to be testis specific [48] and the BORIS binding site has been mapped to the testis-specific *CST* promoter [9]. The alignment of human and mouse BORIS binding site in *CST* promoter is shown at the bottom. The 5 cytosines boxed in the alignment are contact nucleotides for BORIS protein as it was determined by methylation interference assays [9]. Nucleotides that are 100% identical to the human nucleotide sequence are shaded in grey. Dashes indicate insertions or deletions. (**B**) and (**C**) Most BORIS isoforms bind to the human and mouse testis-specific *CST* promoter in EMSA. The number of ZFs and utilization of alternative N-and C-termini for each BORIS isoform are shown at the bottom of gel. The band shifts specific for particular N- and C-termini are indicated by arrows. Luciferase and CTCF were used as negative and positive controls, respectively. (**D**) The mouse *CST* promoter is transcriptionally activated by BORIS isoforms in HEK293T cells. HEK293T cells were either co-transfected with mouse *Boris,* mouse *Ctcf,* empty pCI vector (EV) or the human *BORIS* isoform constructs, as well as with pGL-3 containing 359 bp of wild type or mutant *CST* promoter. Luciferase assays were done 48 h after transfections. All luciferase activities were normalized for transfection efficiency by measuring the Renilla luciferase activity from the co-transfected pRL-TK vector. Error bars are standard deviations.

We recently identified a unique testis-specific promoter for the mouse *CST* (cerebroside sulfotransferase) gene as a target transcriptionally regulated by BORIS [9]. In EMSA assays, a 120 bp human *CST* promoter probe was specifically shifted by 13 of 17 *BORIS* isoproteins (Fig. 8B,C). Because of the high conservation of this target site between human and mouse *CST* genes (Fig. 8A), BORIS isoforms bound comparably to both the human and mouse *CST* DNA probes (Fig. 8B,C). As for binding to the *H19* ICR, the binding of BORIS isoforms to the *CST* probe was unaffected by alternative amino- and carboxy-termini, whereas the degree of band migration generally correlated with specific protein termini.

Remarkably, the *CST* probe was bound by four more isoform proteins (A3, C4, C7 and C8) than the probe for the *H19* ICR (Fig. 7A and Fig. 8B,C). This difference in target specificity could be ascribed to the number and composition of ZFs in the DNA binding domain of a specific isoprotein. For example, while nine ZFs were required for binding of BORIS isoproteins to the *H19* ICR, only five were required to bind the *CST* probe. BORIS isoproteins bound to the *CST* probe also produced fewer additional band-shifts compared to those seen on binding to the *H19* ICR. The B6, A4, C5 and C6 isoproteins did not bind the *CST* probe (Fig. 8B,C). C6, which has a hybrid ZF4/ZF9 zinc finger (Fig. 2B), did not bind the *CST* promoter, despite having a total of 5 ZFs, as many as isoproteins C4 and C7 (Fig. 2A and Fig. 8B,C). This reinforces the suggestion that properties of individual ZFs are important for DNA binding specificity and provides additional evidence for potential functional distinctions among isoproteins in vivo.

Finally, if binding of the *H19* ICR and *CST* promoter by CTCF and BORIS isoproteins is compared using the same reaction conditions, BORIS isoforms bind the *H19* ICR comparably to CTCF (Fig. 7A). At the same time, binding of CTCF to the *CST* probe was evidently much weaker than that of BORIS isoproteins (Fig. 8B,C). This suggests that target preference for different DNAs is characteristic not only of BORIS isoproteins, but also discriminates between BORIS isoproteins and CTCF.

### BORIS isoproteins are potential transcriptional activators of the testis-specific *CST* promoter

The fact that some BORIS isoproteins can bind the same targets as CTCF (Fig. 7 and Fig. 8) prompted us to determine if the functional consequence of binding to a given target would be comparable for both CTCF and BORIS isoproteins. The potential to regulate transcription is one functional readout of such binding. To this end, we transiently co-transfected a luciferase reporter under the control of either a wild type or a mutant mouse *CST* promoter together with expression constructs for mouse *Boris* and *Ctcf*, and each human *BORIS* isoform (Fig. 8D). The wild type *CST* promoter was activated more than 3-fold by co-transfection of human BORIS isoforms B0, B1, A5, A6 and C3 (Fig. 8D). Isoforms A3 and C8 showed were at least 1.5-fold more active on the *CST* promoter. The most potent activator was the full-length mouse BORIS protein, while mouse CTCF was relatively inactive (Fig. 8D) despite its ability to form a *CST*-CTCF complex *in vitro* (Fig. 8B,C). Thus, the functional outcomes of CTCF and BORIS isoform binding to the same target can differ and may reflect a specific biologic role of BORIS in the regulation of some genes, such as *CST*, that are co-expressed with *BORIS* in testis.

BORIS isoforms that bound both the mouse and human *CST* probes in EMSA (Fig. 8B,C) and had the long N258 amino-terminus in common were able to activate the *CST* promoter *in vitro*, indicating that N258 contains a transcriptional activation domain. Accordingly, isoforms B2, B3, B4 and B5 that lack N258 could bind to *CST* (Fig. 8B,C), but did not induce transcription above background (Fig. 8D). Thus, a ZF domain with a short amino-terminus and alternative carboxy-termini is not sufficient to activate transcription. In parallel with the wild type *CST* promoter, we used a *CST* promoter with mutations of contact nucleotides that abrogate BORIS binding. We observed that the mutant promoter, although driving luciferase expression less efficiently than the wild-type promoter, was nonetheless clearly unresponsive to co-transfection with BORIS isoproteins, solidifying the evidence for DNA target sequence specificity of BORIS isoforms as a requirement for activation of the *CST* promoter.

### DNA methylation, p53 and CTCF are responsible for *BORIS* isoform repression in somatic cells

**Figure 9:**
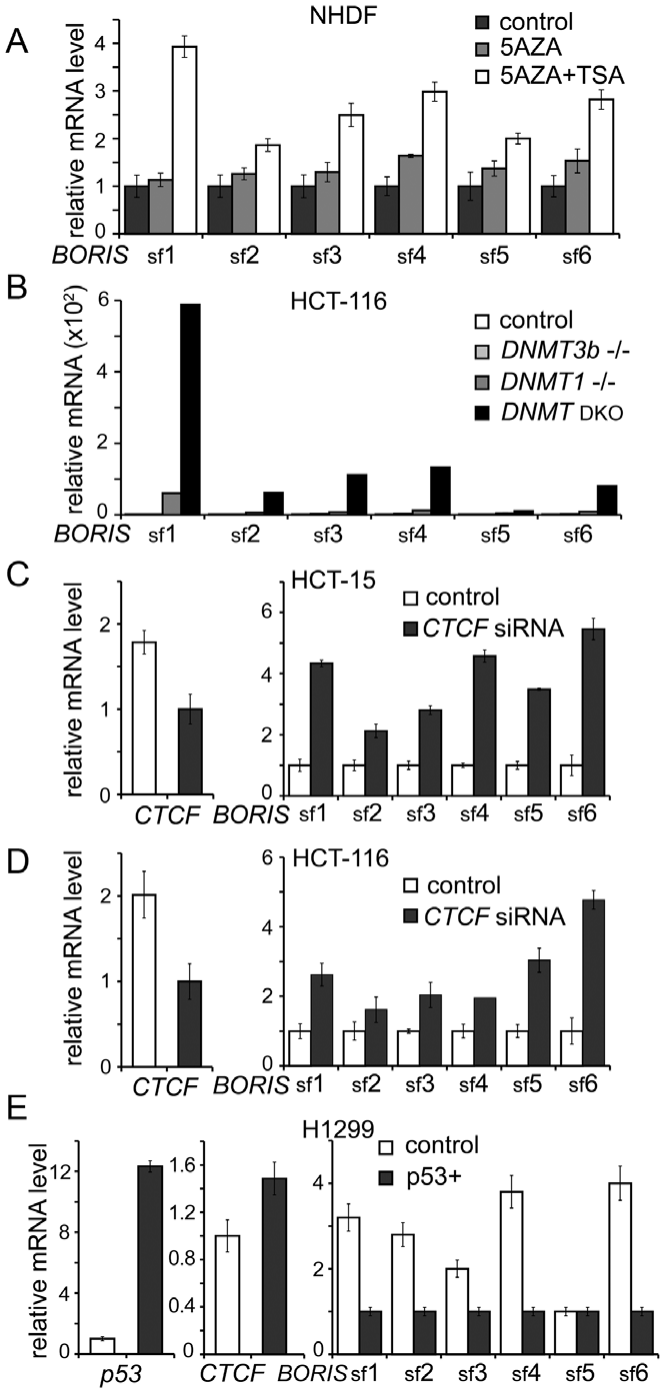
CpG methylation, p53 and CTCF are responsible for *BORIS* isoform repression in somatic cells. (**A**) *BORIS* isoform expression is activated by 5-aza-dC treatment of Normal Human Dermal Fibroblasts (NHDF). qPCR analyses were done on total RNA extracted from NHDF treated with 5-aza-dC alone or in combination with Trichostatin A (TSA). Data were analyzed by comparing Ct values and normalized to the control (untreated cells). (**B**) **A**ctivation of *BORIS* isoforms transcription correlates with genomic DNA demethylation. qPCR was used to quantify *BORIS* subfamily expression in 4 types of HCT116 colon cancer cell lines: the HCT116 parental cell line, two single knockouts for DNMT1–/– and DNMT3B–/– and the double knockout (DNMT1−/−,DNMT3b−/−) (DKO). Data were normalized to *BORIS* isoform levels in the HCT116 parental cell line. (**C**) and (**D**) Knockdown of CTCF in two colon cancer cell lines results in the activation of all *BORIS* subfamilies. qPCR was done on RNA extracted from HCT-15 and HCT-116 parental cells, and from the same cells treated with siRNA against *CTCF* for 72 hours. *CTCF* downregulation was confirmed and *BORIS* upregulation was observed in both cell lines by qRT-PCR. The relative expression levels of *CTCF* and *BORIS* isoforms was determined by the comparative Ct method and normalized to control siRNA. (**E**) Activation of *p53* in H1299 lung cancer cell line results in the suppression of all *BORIS* subfamilies. qPCR on RNA extracted from parental H1299 and from H1299 with exogenously dox-induced p53 wild type shows upregulation of p53 and downregulation of *BORIS* isoforms. Data were analyzed as in (**C**) and normalized to the expression levels of *p53, CTCF* and *BORIS* subfamily expression in the H1299 parental cell line.

The genome-wide pattern of DNA methylation is a key framework of epigenetic phenomena in humans [17]. Recently, CTCF was shown to play key role in maintenance of DNA methylation patterns in normal differentiated cells [30], [31]. Moreover, demethylation of normal human fibroblasts induced by 5aza-dC results in the activation of *BORIS B0* expression [8]. Furthermore, as shown in Figure 4C, *BORIS* isoforms are highly expressed in spermatocytes of adult human testis where the levels of CpG methylation are known to be reduced [2]. Demethylation of the *BORIS* promoter was also linked to *BORIS* expression in cancers [16], [27], [32]. Therefore, we tested whether *BORIS* isoforms could be activated by treating human fibroblasts with 5aza-dC or/and the histone deacetylase inhibitor, TSA. All six *BORIS* subfamilies were effectively activated in cells treated with both agents together (Fig. 9A). sf1 appeared to be more responsive to DNA demethylation than the other subfamilies, an observation in keeping with pattern 1 of *BORIS* isoform expression in cancer cell lines illustrated in Figure 5B.

To independently verify that *BORIS* isoforms could be activated by DNA demethylation, we analyzed their expression in four variants of the HCT116 colon cancer cell line: the parental cell line; the single knockouts (KO) of *DNMT1* and *DNMT3B* in which DNA methylation is reduced 20% and 3%, respectively; and the double knockout of *DNMT1* and *DNMT3B* with genomic methylation reduced by 95% [21]. The transcript levels of *BORIS* isoforms in the parental cell line were used as a baseline to evaluate the effects of DNA demethylation. The modest reduction of DNA methylation characteristic of *DNMT3b* KO cells was not sufficient to activate *BORIS* transcription, while the greater reduction of DNA methylation in *DNMT1* KO cells was associated with low level activation of all *BORIS* subfamilies. The highest levels of *BORIS* isoform expression were observed in the double KO cells, confirming the idea that DNA methylation has a cumulative repressive effect on expression of *BORIS* isoforms (Fig. 9B). sf1 was more sensitive to DNA demethylation than the other subfamilies, similar to results shown in Figure 9A. Thus, the efficiency with which alternative *BORIS* transcripts can be activated correlated with genomic DNA demethylation in a dose-dependent fashion. This dependence likely reflects a direct contribution of DNA methylation to transcriptional repression of *BORIS* in somatic cells.

In addition to DNA methylation, both CTCF and p53 have been shown to repress transcription of *BORIS B0* [8], [16]. Furthermore, multiple methylation-sensitive and methylation-insensitive CTCF target sites were mapped in all three *BORIS* promoters [16]. To characterize the effect of *CTCF* downregulation on expression of all *BORIS* subfamilies, the colon cancer cell lines, HCT-15 and HCT-116, were transiently transfected with *CTCF* siRNAs. A less than 2-fold suppression of *CTCF* resulted in elevated expression of all subfamilies (Fig. 9C,D). The pattern of *BORIS* isoform upregulation was similar in both cancer cell lines, suggesting a common mechanism of *BORIS* isoform repression by *CTCF* at least in some cancer cell lines.

Wild type p53 was previously shown to be an independent repressor of *BORIS* expression with the levels of *BORIS* transcripts in the NCI-60 cancer cell lines being correlated with deletion of *p53* [16]. p53 likely acts by binding to SP1 sites mapped in the *BORIS* promoter [16]. To detail the effect of *p53* on expression of *BORIS* isoforms, we induced ectopic *p53* transcription in the H1299 lung cancer cell line, which is homozygous for deletion of *p53*. While *BORIS* subfamilies were expressed at very high levels in parental H1299 cells, the induction of *p53* for 48 hours resulted in strong suppression of all subfamilies, except sf5 (Fig. 9E). Incidentally, *p53* upregulation also led to a marginal upregulation of *CTCF*. Thus, the suppressive effect of p53 on *BORIS* isoforms may be due, at least in part, to upregulation of *CTCF* (Fig. 9E). In summary, p53 effects, CTCF binding, and DNA methylation provide three interdependent mechanisms for the complete suppression of *BORIS* isoforms expression in somatic cells, as a means of avoiding BORIS competition with CTCF for its functions [16].

## Discussion

### *BORIS* is expressed as multiple isoforms in germ cells

CTAs are expanding family of proteins normally restricted to germ cells that are nevertheless detected in many cancers [26]. As a member of CTA family, BORIS is critical to normal germline development [9] and may also play a role in oncogenic transformation [34]. Therefore, understanding the potential roles of BORIS isoproteins is imperative for deciphering these complex pathways. In the present work, we demonstrate that testis-specific mRNAs originating from the *BORIS* locus comprise at least 23 alternative transcripts that have the potential to encode 17 distinct proteins with varying number of ZFs and different combinations of amino- and carboxy-termini (Fig. 2, Fig. S1, Table S3). Alternative splicing and utilization of alternative transcription initiation sites can greatly expand the coding capacity of a gene, and are known to be utilized more frequently during organogenesis of complex organs, such as brain or testis [35]. All the structural differences among the *BORIS* isoforms could prove necessary for specialized functions in the testis, including differential binding to DNA targets and interactions with alternative partner proteins.

As sequences of most *BORIS* isoforms have considerable overlap and frequently have similar transcript sizes, it was impossible to detect expression of each *BORIS* isoform by conventional methods (Fig. 2, Fig. 5, Fig. S4, File S1 and File S2). The subdivision of the 23 *BORIS* isoforms into 6 subfamilies, sf1 to sf6, according to unique 3′ sequences, enabled the robust detection and quantification of subfamily expression using 6 *BORIS* specific qRT-PCR probes and RPA riboprobes (Fig. 2 and Fig. 5D-J). All six *BORIS* subfamilies were expressed in adult testis with sf1 being the dominant set. Subfamilies notably differ in their promoter usage: sf1 and sf3 express from all three *BORIS* promoters; sf4 expresses from promoters A and C; sf5 expresses only from promoter B; while sf6 utilizes promoters A and C. The nature of the relationship between promoter usage and isoform identity requires further investigation.

*BORIS* isoforms were expressed at low levels in the early stages of spermatogenesis, including spermatogonia, compared to the later stages represented by spermatocytes and spermatids (Fig. 4C). Spermatids were only slightly positive for sf5 and sf6 but were strongly positive for sf2 (Fig. 4C). This may indicate a developmentally controlled shift in the repertoire of isoforms, possibly signifying distinct functions for individual isoforms during male germline development. The characterization of specific functions of BORIS isoforms in testis would, however, require the establishment of an experimental model, such as primates, that would parallel development in humans.

### *BORIS* isoforms may have unique functions in ovaries

It has been reported that, in addition to testis, *BORIS* isoform *B0* is expressed in human oocytes but not in adult ovaries [22]. This is consistent with our results demonstrating that, among 13 fetal tissues tested, only ovary was strongly positive for all six *BORIS* subfamilies, but with varying levels of expression. The inability to detect strong *BORIS* isoform expression in adult ovaries is likely due to the fact that the contribution of oocyte to total ovarian RNA diminishes with age. We presume that the strength of *BORIS* isoform expression in fetal ovary can be attributed to oocytes-specific transcripts. In this regard, *BORIS* isoforms are likely to have some functions in oocytes, as early embryos rapidly become *BORIS*-free [9].

A significant observation from the present work is that the ovary-specific pattern of *BORIS* isoform expression is distinct from that of adult testis (Fig. 4A,B). This may be due to the fact that germline development in males is dramatically different from that in females. With respect to meiosis, however, adult testis and fetal ovaries are approximately at the same stage of maturation. Thus, the differential expression of *BORIS* isoforms in fetal ovaries and adult testis may suggest that isoforms are both functional and specialized in these two germlines.

### *BORIS* isoforms are aberrantly activated in a wide range of cancers

One of the common characteristics of germ cell development and cancer progression is the expression of CTAs, including BORIS (catalogue number CT27) [26]. Studies from our laboratory and others provide evidence that *BORIS* is aberrantly activated in cancer cells of diverse origins [2], [6], [7], [8], [25], [27], [32], [33]. The aberrantly activated BORIS isoproteins may play the role of a “mutant CTCF” in cancers, possibly through the competition of BORIS with CTCF. These features prompted consideration of *BORIS* as both a diagnostic biomarker in breast cancer [33] and as a target for anti-cancer immunotherapy [24], [36]. Furthermore, *BORIS* is included in the priority-ranked list of cancer vaccine target antigens [37].

Proteins encoded by *BORIS* isoforms can be considered as CTAs, based on their expression in spermatocytes and upregulation in a wide range of cancers (Fig. 4 and Fig. 5). Using qPCR, we showed that all six *BORIS* subfamilies were expressed in 70% of the NCI-60 cancer cell lines although most lines had fewer than 1500 total *BORIS* transcripts per 50 ng RNA. This level of expression represents less than one transcript per cell, although this could be an underestimate due to limitations in primer selection for isoform detection (Fig. 2). The low levels of expression determined here are in agreement with prior studies of cancer tissues and cell lines, which showed that expression of the *BORIS B0* isoform was dependent on tumor stage, metastatic status and type of cancer [8], [25], [27]. Similar features have been documented for many other CTAs that showed restricted expression in a small fraction of the tumor cell pool [26], [38], [39]. Elevated expression of CTAs has been shown to characterize subpopulations of cells from melanomas and gliomas [38], [39]. Expression of BORIS in cancer may therefore be limited to a subset of cells or to a particular stage in tumor development. Further work on *BORIS* isoform expression in cancer cell lines and primary tumors is needed to verify this suggestion.

Results of the present work indicate that not only all *BORIS* promoters but also the alternative splicing within the *BORIS* locus are abnormally activated in cancers. The expression of individual subfamilies differs between normal and cancer cells since neither the pattern 1 nor pattern 2 of isoform expression in the NCI-60 panel resembled the expression profiles of adult testis or fetal ovaries (Fig. 4 and Fig. 5). Aberrant promoter demethylation possibly contributes to *BORIS* activation in cancer cells, as indicated by the emergence of pattern 1, described in Figure 5B, with a marked predominance of sf1 isoform expression. Indeed, complete demethylation of promoter B correlated with the highest levels of *BORIS B0* isoform expression among NCI-60 cancer cell lines [16]. Furthermore, while the profile of *BORIS* isoform expression in parental HCT-116 cells matched pattern 2 of *BORIS* isoforms expression in cancers with sf1-4 and sf6 are expressed at about equal levels (Fig. 5C), the double KO cells resembled the first pattern (Fig. 9B). As DNA methylation in double KO HCT-116 cells was reduced by 95% compared to wild type, the pattern 1 of *BORIS* isoform expression is likely associated with extensive hypomethylation of the CpG island in *BORIS* promoter [16].

Apart from CpG methylation, *BORIS* isoform expression is decreased in response to p53 activation (Fig. 9E). This highlights the potential importance of *BORIS* isoform expression as a component of oncogenic cell transformation and agrees with our earlier study on *BORIS* promoters showing negative regulation by p53 [16].

### DNA binding and transcriptional properties of BORIS isoforms compared to CTCF: similarities and distinctions

There are two possible scenarios for BORIS competition with CTCF when both genes are expressed in the same cell. First, BORIS and CTCF may directly regulate expression of each other. Indeed, we previously showed that there are CTCF binding sites in all three *BORIS* promoters and that *BORIS* expression is induced in response to downregulation of *CTCF* [8], [16]. Furthermore, genome-wide ChIP studies indicated that there are multiple sites inside *BORIS* exons and introns that are occupied by CTCF [40], indicating possible additional levels of regulation. Second, CTCF and BORIS compete for binding to sites in regulatory elements of other genes [8], [41].

The transcriptional effects of *CTCF* downregulation on its target genes, all reported to have *CTCF* sites within their promoters, were ascribed to either direct or downstream effects of reduced CTCF occupancy at specific sites [30], [31], [42]. Our results showed that downregulation of *CTCF* can lead to the increased expression of multiple *BORIS* isoforms (Fig. 9C,D), indicating that competitive BORIS binding is another possible contribution to CTCF downregulation.

Characterizing BORIS isoform-specific functions and activities that overlap with those of CTCF in vivo is a daunting task. Nonetheless, characterization of the DNA binding specificities of BORIS isoforms is a first essential step. All 17 BORIS isoproteins contain a ZF DNA binding domain that can mediate recognition of specific DNA targets. CTCF binds DNA with a wide range of sequence specificities [1] making it uniquely multifunctional [5]. The differing number and composition of ZFs in BORIS isoproteins may well expand the established multifunctionality of the 11 ZF domain. Nevertheless, our studies showed that BORIS isoproteins bind DNA in a fashion that is generally similar to that of CTCF, at least for the two sites studied here. This similarity is likely to be based on the high degree of identity (74%) between the ZF regions of the two proteins and the conservation of the major DNA recognition residues at the positions minus one, two, three and six within each ZF [6]. Furthermore, the ZF domains of BORIS isoproteins are both sufficient and necessary for DNA binding (data not shown). In addition to the utilization of various sets of ZFs to bind different sites, as was demonstrated for CTCF [6], BORIS isoproteins include additional forms with different numbers and composition of ZFs. For example, binding of BORIS isoproteins to the *H19* ICR required at least nine ZFs (Fig. 7A), whereas only five ZFs were required for binding the *CST* promoter probe (Fig. 8B,C). Like CTCF, the efficiency of BORIS isoprotein binding to DNA *in vitro* correlates with the number of ZFs in the DNA-binding domain [43], while the ZF flanking domains of the isoproteins likely contribute to complex “architecture”. For example, the five ZFs isoproteins C4 and C7 bound *CST* probes more weakly than isoproteins with more ZFs (Fig. 8B,C). The effects of amino-terminal truncations were also comparable for BORIS isoproteins and CTCF (Fig. 7 and Fig. 8). BORIS isoproteins with a long N-258 terminus produced equally high band-shifts with different DNA targets, essentially regardless of other protein domains. The truncation of N258 in isoproteins B2, B3, B4 and B5 had no impact on DNA binding per se, but resulted in a dramatic increase in EMSA complex migration (Fig. 7 and Fig. 8). This effect is similar to that of the deletion of the amino-terminus of CTCF [44], despite the fact that the amino-terminal domains of CTCF and BORIS have less than 10% sequence homology.

CTCF is recognized as a major regulator of epigenetic pathways in mammals [5] while the evidence of BORIS involvement in imprinting and other epigenetic phenomena remains fairly limited [10]. Here we showed that binding of BORIS isoproteins to *H19/IGF2* ICR DNA is sensitive to methylation (Fig. 7B) as was previously demonstrated for CTCF [13]. These data are in agreement with a recently published study [45] but contrast with findings described in another publication [29]. In general, methylation sensitive CTCF binding sites, when binding BORIS isoproteins, do so also in a methylation-sensitive manner.

In spite of the described similarities between BORIS isoproteins and CTCF, their biology *in vivo* suggests the existence of non-overlapping functions. Functional specialization of BORIS isoforms could be generated by interactions with alternative partner proteins, and by novel DNA binding activities of isoproteins that do not correspond to CTCF-recognized sites. In BORIS isoproteins, sequence divergence between the ZF domains, differing numbers and utilization of ZFs, and the absence of the CTCF AT hook motif may account for such differences [6]. We also demonstrated that not every DNA target exhibited equivalent binding of CTCF and BORIS. For example, CTCF binding to the *CST* probe was weaker than for BORIS isoproteins under the same EMSA conditions (Fig. 8B,C). This could illustrate the potential for BORIS isoproteins, despite their lower abundance, to compete with CTCF for site occupancies genome wide.

We recently reported BORIS functions as a transcription factor to regulate a subset of genes in germ cells [8], [9]. In this case, BORIS functions in gene expression could not be compensated by CTCF in *BORIS* KO mice [9]. EMSA showed that BORIS isoproteins could bind a probe from the testis-specific promoter of *CST* (Fig. 8B,C). Furthermore, isoproteins having the long N258 terminus with at least the first five ZFs could activate this promoter in transient luciferase assays (Fig. 8D). The isoproteins lacking the N258 terminus failed to activate reporter transcription, despite being able to bind the *CST* probe in EMSA (Fig. 8). Thus, the N258 terminus apparently harbors an activator domain. Incidentally, the N267 terminus of CTCF was previously implicated in transcriptional activation of *APP* promoter [43] even though there is only a low degree of similarity between the amino-termini of CTCF and BORIS N258 isoproteins. The correlation between the N258 contribution to the size of the BORIS isoform complexes in EMSA and its transcriptional activity suggests that changed protein conformation and/or stoichiometry in BORIS/DNA complexes contribute to its transcription activation potential. Our data thus support the view that BORIS isoproteins can both partially replace and interfere with CTCF in germline development and cancers, in manners dependent on the nature of the specific target site. The fact that BORIS has multiple isoproteins may enhance the range of potential binding sites over the CTCF target sites. Future studies analyzing the occupancy of CTCF target sites by BORIS isoproteins and expression profiles of potential targets in germ cells and cancers will be needed to decipher the functions of BORIS isoproteins.

### The potential functional significance of multiple BORIS isoforms in the germline

While specialized functions of individual BORIS isoproteins remains to be documented, it is likely that expression of multiple isoforms from the human *BORIS* locus is biologically important. This is indicated first, by the differing isoform repertoires in male and female germ cells (Fig. 4) and the distinct patterns of expression in different cancer cell lines (Fig. 5B,C). Second, BORIS isoforms are evolutionarily conserved in primates. It is generally accepted that alternative splicing, if conserved among different species, is likely to reflect biologically important variations [35]. In this report, we describe the high conservation of splicing sites and alternative coding sequences in *BORIS* loci in primates, including *Homo sapiens*, *Pan troglodytes* and *Macaque mullata* (Fig. 3). Third, an alternative ZF is found in isoform *C6* (Fig. 2B), and such precise “protein engineering” is unlikely to be accidental. Even though this isoform does not bind the probes we tested, it probably has a specific set of targets *in vivo*. Another “naturally engineered” isoform, *A3*, while binding weakly to the *CST* probe (Fig. 8B,C), has shown some transcriptional activation potential on other targets *in vivo* (to be published elsewhere). Finally, the different transcriptional activity of individual isoproteins supports the idea of biologically relevant functional specialization.

What could be the functional advantages of expressing multiple BORIS isoproteins in the germline? Spermatogenesis and oogenesis are multistage processes, involving tightly controlled cell-specific activation and/or repression of a wide spectrum of genes at every stage of germ cell development. The sheer complexity and tight regulation of this multistep process apparently requires engagement of active alternative splicing for multiple genes, especially transcriptional factors in germline. One prominent example is CREM (cAMP Responsive Element Modulator) that regulates expression of many male fertility genes and is expressed through all stages of spermatogenesis. The *CREM* locus produces multiple isoforms with either activation or repressive activities depending on exon configuration in the alternative transcripts [46]. The fact that some *BORIS* isoproteins can bind the *CST* promoter and activate transcription while others can bind the same promoter but do not display transcriptional activity (Fig. 8) suggests that *BORIS* isoforms, similarly to *CREM,* can perform opposing functions with the same DNA target.

Taken together, the data in this study demonstrate that the *BORIS* gene is much more complex than initially appreciated. Our results demonstrate that multiple isoforms expressed from human *BORIS* gene encode isoproteins that can bind DNA and function as transcription factors. These proteins have the potential to compete with CTCF for binding to DNA and can possibly modulate or block CTCF functions at these targets in vivo. Although our data have significantly enhanced the understanding of *BORIS* complexity, additional studies are needed to ascertain the significance and the functional role of each *BORIS* isoform.

Ethics Statement: N/A

## Materials and Methods

### Cell lines and treatment

Cancer cell lines or primary normal cells were either described previously [8], [16] or were obtained from the ATCC (Manassas, VA) and were grown as suggested by distributors. The NCI-60 cancer cell line panel was obtained from the National Cancer Institute. The NCI-60 cell lines were cultured in RPMI with 5% FBS and antibiotics. Wild type, DNMT1−/−, DNMT3b−/− and DKO (DNMT1−/−, 3b−/−) HCT116 colorectal cancer cell lines were provided by Dr. Bert Vogelstein (Johns Hopkins Medical Center, Baltimore MD) and were cultured in McCoy's 5A medium supplemented with 10% FBS and antibiotics. The H1299 lung cancer cell line with a wild type, stably-transfected, doxycycline-inducible p53 expression vector, a gift from Dr. Peter M. Chumakov (Lerner Research Institute, Cleveland, OH), was cultured in DMEM with 10% FBS. HCT-15 and HCT-16 colon cancer cell lines from the NCI-60 panel were treated for 72 h with freshly prepared 5 mM 5-aza-2′-deoxycytidine (5-azadC) (Sigma-Aldrich, St. Louis, MO) and/or with 3 mM histone deacetylase inhibitor, Trichostatin A (TSA) (Sigma), dissolved in ethanol.

### RNA ligase-mediated rapid amplification of cDNA ends (RLM-RACE)

To isolate full length *BORIS* transcripts, we used the 3′ RLM-RACE approach (Fig. 1C). Full-length RLM-RACE libraries were generated using total RNA from adult testis and K562 cells, as suggested by the manufacturer manual (Invitrogen, Catalog number: L1500-01). As promoter utilization may have some influence on splicing regulation, the three forward primers - A, B, and C – were designed to amplify mRNAs expressed from the respective promoters (Fig. 1C, Table S1). As a reverse primer we used 3′ GeneRacer oligo attached to a polyA tail during first-strand cDNA synthesis. To increase the yield and specificity of RACE-PCR products, a second nested round of PCR amplification was performed using 1 µl of the first round reaction. PCR with Platinum Taq polymerase (Invitrogen) was done for 40 cycles of 30 s at 95°C, 30 s at 60°C and 4 min at 72°C with a final extension at 72°C for 7 min. Amplified products were separated by electrophoresis in 1% agarose gels, excised, purified by Gel Extraction Kit (QIAGEN Inc, Chatsworth, CA), cloned into the pCR®2.1-TOPO® vector (Invitrogen), and sequenced using LI-COR sequencing system (LI-COR Biosciences Inc., Lincoln, NE).

### RNA and cDNA samples used in RT-PCR assay

25 mg of total RNA from 13 adult and 11 fetal tissue samples were from Stratagene, (La Jolla, CA). FastClone RACE-Ready cDNA from fetal testis and fetal ovary was from Spring Bioscience (Fremont, CA). Another source of fetal ovary (28 weeks old) was obtained from BioChain (Hayward, CA). Five different sources of total RNA from adult testis were analyzed for *BORIS* isoform expression: 1) the lowest level of *BORIS* transcripts (10-fold lower level of *BORIS* isoforms expression, compared to four followed samples) was detected in testis RNA obtained from OriGene Technologies (Rockville, MD, Catalog Number: HT-1011). 2) testis RNA from 72 year old male (Stratagene). 3, 4, 5) Total RNA was extracted by Trizol from normal adult testis samples from 23, 21 and 29 year old males (NDRI, Philadelphia, PA).

### CTCF knock down by RNAi

HCT-15 and HCT-116 cell lines were transfected with 5 mM of human CTCF ON-TARGETplus SMART pool siRNA (Dharmacon Inc., Lafayette, CO). A siCONTROL Non-Targeting siRNA (Dharmacon Inc.) was used as negative control in all siRNA experiments. Cells were harvested after 48 or 72 h for western and quantitative RNA expression analysis.

### Standard RT-PCR and Quantitative Real-Time RT- PCR (qPCR)

Total RNA was incubated at 70 C for 10 min, cooled on ice, and then cDNA was synthesized using a ThermoScript reverse transcription kit (Invitrogen), using an oligo(dT)18 primer. The reverse transcription reaction was performed as follows: 42°C for 60 min, 85°C for 10 min and 4°C for 10 min. 2 µL of cDNA was amplified in a 50 µL volume containing Platinum Taq DNA polymerase (Invitrogen). The reaction mix was incubated for 35 cycles (95 C for 30 s, 60 C for 30 s, 72 C for 1–3 min). The primers used in RT-PCR are shown in Table S1. To analyze the expression patterns of *BORIS* isoforms in normal and cancer cells, *BORIS* isoforms were divided into six subfamilies, sf1 to sf6, based on their unique 3′ sequences (Fig. 2A). The Taqman probe sf1 was designed against sequences between exon 9 and 10 of the *BORIS B0* and detects *BORIS* isoforms *B0, B1, A1, A2, A3*, and *C1* (Fig. 2, File S1, Table S3). The absolute quantification approach was applied to estimate the actual number of *BORIS* transcripts detected by sf1 per 50 ng of total RNA. *BORIS B1* contains the unique splice site that was used to design the sf5 probe and the total number of *B1* transcripts was subtracted from the total number of transcripts detected by the sf1 probe. The Taqman probe sf2 detects at least two *BORIS* isoforms, *A4* and *C2,* that produce the same protein but are expressed from two alternative promoters, A and C, respectively. The Taqman sf3 probe detects five isoforms: *A5, A6 B4, B5,* and *C6*. The Taqman probe sf4 was designed to detect at least six *BORIS* isoforms: *C3, B2, B3, C4, C5,* and *C8*. The *B1* isoform has a unique C-terminus and 3′ UTR that were used to design the sf5 probe. The sf6 probe detects four *BORIS* isoforms: *B6, B7, C7*, and *C9*.

For absolute and relative quantification of *BORIS* alternative transcripts as well as *CTCF, GAPDH* and *p53* expression, qRT-PCR was done using the ABI Prism 7900 Sequence Detection System (Applied Biosystems, Foster City, CA). 5 µl of each cDNA corresponding to 50 ng of total RNA was amplified in a total volume of 11 µl using the universal PCR Master mix (Applied Biosystems, Foster City, CA) with 250 nM of primers and 200 nM Tagman probe. Amplification conditions were: 50°C for 2 min, 95°C for 10 min, followed by 45 cycles of 95°C for 15 s and 60°C for 1 min. All reactions were prepared in triplicate, and at least three independent runs were performed for each sample. Primers to analyze *BORIS* alternative transcripts were selected using Primer Express Software (Applied Biosystems). The efficiencies of all PCRs were above 95%. All specific primers and Taqman probes are listed in Table S2. Expression of *GAPDH* was used as an endogenous control. The relative quantification of gene expression was done using a comparative 2-ddCt method (Applied Biosystems). The absolute quantification of gene expression was as described previously [16].

### Vector construction and transfections

The open reading frames of 17 alternative *BORIS* transcripts, encoding 17 BORIS isoproteins, were initially amplified by nested RT-PCR with cDNA from K562 cell line and/or from normal testis using primers shown in Table S1. The amplification introduced a HA-Tag at the N-terminus of each *BORIS* isoform as well as 5′-*Nco*I and *EcoR*I restriction sites and 3′-*Not*I and *Nhe*I sites, to facilitate subsequent cloning into different vectors. PCR products after the second round of amplification were cloned into the pCR2.1-TOPO vector (Invitrogen) and sequenced. The pCITE4a vectors (Novagen, Madison, WI) for 17 BORIS isoproteins, which were used for *in vitro* transcription/translation, were generated by re-cloning *EcoR*I- and *Not*I- fragments from TOPO vectors into pCITE4a vector cleaved with the same enzymes. The vectors used for luciferase co-transfection experiments were made by cloning the same fragments into the pCI vector (Promega, Madison, WI). The *BORIS*-DsRed constructs that were used in transient transfections to detect the localization of BORIS isoproteins were generated by making the *EcoR*I- and *Not*I- ORF fragments blunt-ended with Klenow enzyme (New England Biolabs, Inc., Beverly, MA) and cloning them into the pDsRed-Monomer-Hyg-C1 vector (Clontech Laboratories Inc., Mountain View, CA) cleaved with *Xma*I followed by Klenow enzyme treatment. The luciferase reporter constructs contained a luciferase gene driven by either a 359 bp wild-type or mutant testis-specific *CST* promoter based on the pGL3-basic vector (Promega), previously described in detail [9]. All transfections were done with the jetPEI Cationic Polymer Transfection reagent (PolyPlus-transfection, Illkirch, France) according to the manufacturer's instructions, except HEK293T cells, which were transfected using TransIt transfection agent according to manufacturer's protocol (Mirus Bio, Madison, WI). All individual transfections used in quantitative assays were performed at least three times. Transiently transfected cells were harvested either 48 or 72 h after transfection. For stable transfection, an antibiotic was added 3 days post transfection and colonies were selected for three weeks.

### Electrophoretic mobility shift assay (EMSA)

Fragments containing mouse and human testis-specific *CST* promoters or the *H19-IGF2* sixth *CTCF* target site were synthesized by PCR with primers shown in Table S1. EMSA was performed as previously described [4]. Briefly, PCR fragments were labeled using ^32^P-γ-ATP with T4 polynucleotide kinase (New England Biolabs). Protein–DNA complexes were incubated for 90 min at room temperature in binding buffer containing 25 mM Tris pH 7.4, 0.1 mM ZnSO4, 5 mM MgCl2, 5% Nonidet P-40 in PBS, 0.25 mM Mercaptoethanol, 10% glycerol and 0.5 µg of poly dI-dC. Protein–DNA complexes were separated from the unbound probe using 5% native polyacrylamide gels (PAAG). Full-length CTCF and 17 BORIS isoforms that were cloned into the pCITE4a vector were translated *in vitro* using a TnT kit (Promega). The equivalent yield of proteins was confirmed by ^35^S methionine incorporation with subsequent PAAG separation and quantification.

### Luciferase Assay

For luciferase assays, 5×10^5^ HEK293T cells were grown in 6-well plates and co-transfected with 2 µg of plasmid DNA using TransIt transfection agent (Mirus Bio). Each transfection included 0.8 µg of pCI plasmid containing the mouse *Ctcf* ORF or mouse *Boris* or human *BORIS* isoforms, 1 µg of pGL-3 containing 359 bp of wild-type or mutant mouse *CST* promoter [9], and 0.2 µg of pRL-TK renilla luciferase vector. After 48 hours, cells were lysed in 200 µl passive lysis buffer (Promega) and analyzed in a Microbeta luminescence counter 1450 (Perkin Elmer, Boston, MA) using a dual luciferase reporter assay kit (Promega). All luciferase activities were normalized for transfection efficiency by measuring the Renilla luciferase activity of the co-transfected pRL-TK vector.

### Northern and western blotting

Northern blotting was done using NorthernMax formaldehyde-based system (Ambion Inc., Austin, TX) according to the manufacturer's recommendations. Briefly, 30 µg total RNA was loaded per lane, separated in agarose gels, transferred to a positively charged nylon membrane (BrightStar-Plus, Ambion Inc.), cross linked by UV, and hybridized with labeled probes at 42°C. The six probes were labeled internally using Strip-EZ PCR Kit (Ambion Inc.) according to the manufacturer's recommendations (Primers shown in Table S1). For western blotting total cell lysates were prepared in SDS-lysis buffer (0.1M Tris/HCl pH6.8, 7M Urea, 10% mercaptoethanol, 4% SDS & phenol red dye). The lysis buffer was added at a ratio of 20 µl per 1×10^5^ cells. Cell lysates were heated at 95°C for 5 minutes, centrifuged at 12000 g for 5 sec., loaded onto SDS gels, transferred to PVDF membrane (Invitrogen), probed with rat monoclonal Anti-HA antibody (1∶2000) (Roche, Indianapolis, IN, Catalog number 13551000), and then probed with peroxidase-conjugated rabbit anti-rat IgG (1∶5000) (Abcam, Cambridge, MA). Proteins were visualized using enhanced chemiluminescence (Pierce, Rockford, IL).

### RNase Protection Assay

cDNAs representing different subfamilies and *BORIS C6* isoform were cloned by RT-PCR using total testis RNA as template. The sequence of primers could be found in Table S1. The PCR bands were extracted from an agarose gel, cloned into pCRII-TOPO vector by TA-cloning (Invitrogen), and sequenced to verify the insertion and antisense orientation. The vector was linearized by *Bam*HI digestion, and riboprobe was synthesized from T7 promoter in the presence of 50 µCi of [α-32P] CTP (3000 Ci/mmol) by MAXIscript Kit according to the manufacturer's recommendations (Ambion Inc.). The DNA template was removed by incubation with RNase-free DNase (1 U/µg of DNA) and the RNA probes were gel purified to isolate only full-length RNA transcripts. 20 µg of total RNA from kidney, testis or the K562 cell line was incubated with riboprobe (7×10^5^ cpm/sample) in 80% formamide, 40 mM PIPES (pH 6.4), 0.4 M NaCl, and 1 mM EDTA overnight at 54°C. RNase digestion and precipitation was performed with RPA III Kit according to the manufacturer's recommendations (Ambion Inc.). RNase protected fragments were resolved on 5% polyacrylamide/8 M urea gel in the presence of size markers to aid in the identification of bands.

### Immunofluorescence and fluorescent in situ hybridization (FISH)

4×10^4^ HEK293T cells were plated in 8-well chambered coverglass (Nalge-Nunc, Naperville, IL) 24 h before transfection. dsRFP-fusion *BORIS* transcripts (200 ng) were introduced into HEK293T cells using TransIT Transfection Reagent (Mirus Bio). After 24–48 h of transfection, cells were fixed in 4% formaldehyde in PBS for 30 min, permeabilized with 0.5% Triton-X100, and stained with 4′, 6-diamidino-2-phenylindole (DAPI, Vector Laboratories, Inc., Burlingame, CA). RFP fluorescence patterns were observed by fluorescent microscope (Axioscop 2 plus, Zeiss, Germany). For FISH assays, the probes for six *BORIS* subfamilies (primers are shown in Table S1) were labeled with digoxygenin-11-dUTP (Roche, Indianapolis, IN) by PCR and individually hybridized to formaldehyde-fixed adult human testis as previously described [47]. The slides were incubated with anti-DIG antibodies overnight and then visualized with rhodamine-conjugated secondary antibody. For identification of spermatogonia and spermatocytes, the frozen testis slides were fixed and incubated with anti-SCP3 (NB300-231, Novus Biologicals, Inc., Littleton, CO; 1∶300 in 3% BSA) and E-cadherin (ab1416, Abcam, Cambridge, MA; 1∶50 in 3% BSA) for 2 hours, followed by incubation with Alexa Fluor 488/546-conjugated (Invitrogen, Carlsbad CA; 1∶500 in PBS) secondary antibody for 1 h. For staining of DNA, DAPI was used.

## Supporting Information

File S1 The full-length nucleotide and deduced amino acid sequence of the individual *BORIS* isoforms.
(0.40 MB RTF)

File S2 *BORIS* isoforms *B0, C2, A5, C3, B1* and *C7/C9* are the dominant representatives of the six *BORIS* subfamilies.
(0.07 MB RTF)

Figure S1 The 23 alternatively spliced RNAs are predicted 17 protein isoforms with three alternative N-termini and eleven alternative C-termini. Unique alternative C-termini labeled by different colors.
(0.70 MB TIF)

Figure S2 Alignment of alternative C-terminus C97, present in BORIS C3, B2, and B3 isoforms, demonstrated a similarity from 52% to 72% with more than 100 human proteins, some of them are involved in transcription or translation processes. The proteins sharing homology with C97 include: transmembrane protein 50B (TMEM50B) (56%), general transcription factor IIH (GTF2H1) (61% identity), seven transmembrane helix receptor (GPR110) (58%), INO80 complex homolog 1 (INO80) (59%), putative calcium-sensing receptor-like 1 (64%), syntaxin 8 (STX8) (72%), vitamin K epoxide reductase complex (VCORC1) (65%), topoisomerase II alpha-4 (TOP2A) (56%), elongation protein 4 homolog (ELP4) (62%), SSU72 RNA polymerase II CTD phosphatase homolog (SSU72) (58%), sarcosine dehydrogenase (SARDH) (53%), translation initiation factor eIF-2B subunit alpha/beta/delta-like protein (MRI1) (60%) and many more.
(3.88 MB TIF)

Figure S3 All BORIS isoproteins are located in nuclei. HEK293T cells were transiently transfected with either *dsRRED* empty vector (EV-dsRED) or with *BORIS* isoforms and *CTCF* fused to *dsRRED* or *GFP* at N-terminus, respectively. RFP and GFP fluorescence were analyzed by microscopy. dsRED protein (Empty Vector (EV)-dsRED) was served as a marker for cytoplasmic location. Cells were also stained with DAPI to visualize nuclear DNA. At the bottom of right column the high magnification image of *BORIS B0* transfected cells are shown to demonstrate the punctual pattern of BORIS nuclear location.
(2.85 MB TIF)

Figure S4 *BORIS* isoforms subfamilies are comprised by distinctly expressed individual isoforms. (A). A schematic illustration of *BORIS* promoters and exons usage for the expression of 23 mRNA isoforms. The rectangular boxes at the bottom denote the locations and sizes of probes that were used for Northern blotting. Arrows correspond to primers that were used in RT-PCR assays. The colors of boxes and arrows correspond to the unique coding alternative sequences as in Figure 2. (B). Northern blotting analysis of *BORIS* isoforms expression in the K562 cell line, adult testis and kidney. The top 6 gels correspond to six internally ^32^P-labeled probes for 6 *BORIS* subfamilies. The bottom gels are loading controls based on ribosomal RNA. The six labeled probes were designed to detect all isoforms of 6 *BORIS* subfamilies. Sequences of primers used to generate Northern probes are shown in Table S1. The dominant transcripts are indicated by arrows with a corresponding name of *BORIS* isoform. The sizes of RNA transcripts are shown on the right side of the membrane. (C). RT-PCR assay to simultaneously detect multiple isoforms within six *BORIS* subfamilies by using one or two set of primers. Agarose gels are shown for every BORIS subfamily (*BORIS* _sf1-sf6). The PCR products were generated from adult testis and the K562 cDNAs by nested RT-PCR. H2O and RNA extracted from heart were used as negative controls for *BORIS* expression. The primers that were used to amplify single or multiple transcripts within one subfamily are named accordingly to their mapping to BORIS exons and shown at the bottom of each gel. These primers are also shown in panel A; the sequence of primers is shown in Table S1. PCR products with the size of expected alternative transcripts are indicated by arrows with the name of corresponding isoform. Subpanel 1). To simultaneously amplify *BORIS B0* and *A3* transcripts within sf1, we used the forward primer from exon 5 (Ex5) and the reverse primer from exon 10 (Ex10). Subpanel 2). For *BORIS* sf2 two pair of primers were used to analyze the expression of *A4* and *C2* isoforms. RT-PCR with forward primers from promoter A (Exa) or C (Ex1c) and reverse primer from the unique sequence of alternative exon 4a (Ex4a) yielded much stronger signal on agarose gels with a forward primer going from promoter C, then from promoter A, suggesting C2 as a main isoform in sf2. Subpanel 3). To distinguish expression of *A6, A5* and *B5* isoforms within sf3, two pairs of primers with the same reverse primer from alternative exon 9a (Ex9a) were used in RT-PCR. The forward primers from exon 4 (Ex4) or from exon 8 (Ex8) amplified three different PCR products corresponding to three *BORIS* alternative forms, indicated by arrows. Subpanel 4). To compare *C3* isoform expression to *C4* and *C5* within sf4, the forward primer from exon3 (Ex3) and the reverse primer from the alternative exon 10b (Ex10b) were used. The forward and reverse primers from non-coding exon b (Ex1b) and exon 3 (Ex3), respectively, generated three PCR products corresponding to multiple *BORIS* isoforms with alternative splice sites from exon b to exon 1 (*BORIS B0*), exon 2 (*B3/B4*) and exon 3 (*B2/B5/B6/B7*). The splice site from exon b to exon1 is more dominant than the splice sites from exon b to exon 2 and 3. Subpanel 5). *B1* isoform was amplified as single form by primers from exon 9 (Ex9) and exon 10a (Ex10a). Subpanel 6). To compare *C7/C9* and *B6/B7* isoforms expression, RT- PCR was done with two forward primers from exon b (Exb) and exon 1c (Ex1c), and with the reverse primer from alternative exon 5a (Ex5a). The primer from the promoter C (Ex1c, *C7/C9*) yielded more abundant PCR product then the forward primer from promoter B (Exb). (D) and (E). Absolute quantification of individual *BORIS* isoforms within sf3 and sf4. The total number of transcripts, detected by both Tagman probes *BORIS_sf3* and *BORIS_sf4*, in the K562 or adult testis was set as 100%. The contribution of single isoform to the total amount of subfamilies' transcripts was calculated by absolute quantification with the series of Taqman probes either to unique sequences or to unique alternative splice sites. The sequence of primers and Tagman probes are shown in Table S2. (D) The sf3 Taqman probe detects 5 *BORIS* isoforms - *A5, A6, B4, B5*, and *C6* (Fig. 2B). Total combined amount of these transcripts was set at 100%, and the amount of single transcripts for 4 out of 5 sf3 isoforms was calculated by absolute qRT-PCR using Taqman probes designed to quantify isoforms *A6* (Ex8-Ex9a1), *B5* (Ex8- Ex9a2), *B4* (Exb-Ex2), and *C6* (Ex4-Ex8). Together, these isoforms represent less than 31% of the total amount of BORIS transcripts detected by the sf3 probe. The percent of main *BORIS A5* isoform contributing to the total amount of all isoforms detecting by the sf3 Tagman probe is shown on the graph. *BORIS A5* and *BORIS C3* were found to be the main isoforms in sf3 and sf4, respectively. (E) The sf4 Taqman probe detects 6 BORIS isoforms - *C3, B2, B3,C4, C5*, and *C8* (Fig. 2A). Total combined amount of these transcripts was set at 100%, and the amount of single transcripts for 5 out of 6 sf4 isoforms was calculated by absolute qRT-PCR using Taqman probes designed to quantify isoforms *B2* (Exb-Ex3), *B3* (Exb- Ex2), *C4* (Ex5-Ex8), *C5* (Ex3-Ex10b) and *C8* (Ex6-Ex6a). Together, these isoforms represent less than 21% of the total amount of *BORIS* transcripts detected by the sf4 probe. The percent of main *BORIS C3* isoform contributing to the total amount of all isoforms detecting by the sf4 Tagman probe is shown on the graph. (F). The schematic illustration of 6 dominant *BORIS* isoforms showing their belonging to 6 *BORIS* subfamilies. (G). Summary table of the 6 dominant *BORIS* isoforms representing the 6 subfamilies.
(1.83 MB TIF)

Table S1 Table of primers used in RT-PCR and PCR assays.
(0.11 MB DOC)

Table S2 The primers and the Tagman probes used in qRT-PCR.
(0.04 MB DOC)

Table S3 The 23 alternatively spliced *BORIS* mRNAs are predicted 17 isoproteins. Table 3 summarizes the information about *BORIS* alternative forms, including the size of alternative transcripts in nucleotides, the size of isoproteins in kDA, the calculated isoelectric point of each protein, the number of ZF per isoform, the alternative N- and C-termini and the GenBank accession number for each BORIS isoform.
(0.05 MB DOC)

Table S4 Exon-Intron Junctions of the *BORIS* gene. The major spliceosome splices introns containing GU at the 5′ splice site and AG at the 3′ splice site.
(0.05 MB DOC)

Table S5 Table of *BORIS* Expressed Sequence Tags (EST) in Gene Bank.
(0.03 MB DOC)
