# Supplementary material for: The Structural Complexity of the Human BORIS Gene in Gametogenesis and Cancer
Source: PLoS One. 2010 Nov 8;5(11):e13872. doi: 10.1371/journal.pone.0013872 (PMC2975627; doi:10.1371/journal.pone.0013872)
Supplement: File S1 — The full-length nucleotide and deduced amino acid sequence of the individual BORIS isoforms. (0.40 MB RTF) [file pone.0013872.s001.rtf]

File S1. 

Full-length nucleotide and deduced amino acid sequence of BORIS isoforms. The start codon (ATG), stop codon (TGA), polyadenilation signal (AATAAA) and poly-A tail are marked by bold and underlined. The deduced amino acid sequence is shown below the nucleotide sequence. The zinc finger coding sequences are highlighted in yellow. The unique alternative amino acid sequences are shown in bold and colored in agreement with colored boxes in Figure 2. The primers used in qRT-PCR to detect the isoform expression are underlined. The Tagman probe showed in italic. The nuclear localization signal is highlighted with red color.


Full-length nucleotide and deduced amino acid sequence of the BORIS A1 isoform (GenBank: DQ778108). BORIS A1 isoform is expressed from promoter A, contains eleven zinc fingers, N-terminus (N258), C-terminus (C95), and 3'UTR belonging to BORIS subfamily 1 (BORIS_sf1). 


attccacccctccccccagtatctcagtgcctcctgtgggccctcctcccctccttatccattccccctcagatctcccaggccccctgcaggccctcg
tgccctccttacttcccccccgggtctcccagcgccccctgcggggccctcctcccttcctcatccacttcaaccccaaggccaagtcattatggcagc
                                                                                            M  A  A  
cactgagatctctgtcctttctgagcaattcaccaagatcaaagaactcgagttgatgccggaaaaaggcctgaaggaggaggaaaaagacggagtgtg
  T  E  I  S  V  L  S  E  Q  F  T  K  I  K  E  L  E  L  M  P  E  K  G  L  K  E  E  E  K  D  G  V  C  
cagagagaaagaccatcggagccctagtgagttggaggccgagcgtacctctggggccttccaggacagcgtcctggaggaagaagtggagctggtgct
  R  E  K  D  H  R  S  P  S  E  L  E  A  E  R  T  S  G  A  F  Q  D  S  V  L  E  E  E  V  E  L  V  L  
ggccccctcggaggagagcgagaagtacatcctgaccctgcagacggtgcacttcacttctgaagctgtggagttgcaggatatgagcttgctgagcat
  A  P  S  E  E  S  E  K  Y  I  L  T  L  Q  T  V  H  F  T  S  E  A  V  E  L  Q  D  M  S  L  L  S  I  
acagcagcaagaaggggtgcaggtggtggtgcaacagcctggccctgggttgctgtggcttgaggaagggccccggcagagcctgcagcagtgtgtggc
  Q  Q  Q  E  G  V  Q  V  V  V  Q  Q  P  G  P  G  L  L  W  L  E  E  G  P  R  Q  S  L  Q  Q  C  V  A  
cattagtatccagcaagagctgtactccccgcaagagatggaggtgttgcagttccacgctctagaggagaatgtgatggtggccagtgaagacagtaa
  I  S  I  Q  Q  E  L  Y  S  P  Q  E  M  E  V  L  Q  F  H  A  L  E  E  N  V  M  V  A  S  E  D  S  K  
gttagcggtgagcctggctgaaactgctggactgatcaagctcgaggaagagcaggagaagaaccagttattggctgaaagaacaaaggagcagctctt
  L  A  V  S  L  A  E  T  A  G  L  I  K  L  E  E  E  Q  E  K  N  Q  L  L  A  E  R  T  K  E  Q  L  F  
ttttgtggaaacaatgtcaggagatgaaagaagtgacgaaattgttctcacagtttcaaattcaaatgtggaagaacaagaggatcaacctacagctgg
  F  V  E  T  M  S  G  D  E  R  S  D  E  I  V  L  T  V  S  N  S  N  V  E  E  Q  E  D  Q  P  T  A  G  
tcaagcagatgctgaaaaggccaaatctacaaaaaatcaaagaaagacaaagggagcaaaaggaaccttccactgtgatgtctgcatgttcacctcttc
  Q  A  D  A  E  K  A  K  S  T  K  N  Q  R  K  T  K  G  A  K  G  T  F  H  C  D  V  C  M  F  T  S  S  
tagaatgtcaagttttaatcgtcatatgaaaactcacaccagtgagaagcctcacctgtgtcacctctgcctgaaaaccttccgtacggtcactctgct
  R  M  S  S  F  N  R  H  M  K  T  H  T  S  E  K  P  H  L  C  H  L  C  L  K  T  F  R  T  V  T  L  L  
gcggaaccatgttaacacccacacaggaaccaggccctacaagtgtaacgactgcaacatggcatttgtcaccagtggagaactcgtccgacacaggcg
  R  N  H  V  N  T  H  T  G  T  R  P  Y  K  C  N  D  C  N  M  A  F  V  T  S  G  E  L  V  R  H  R  R  
ctataaacatactcatgagaaaccctttaaatgttccatgtgcaagtatgccagtgtggaggcaagtaaattgaagcgccatgtccgatcccacactgg
  Y  K  H  T  H  E  K  P  F  K  C  S  M  C  K  Y  A  S  V  E  A  S  K  L  K  R  H  V  R  S  H  T  G  
ggagcgcccctttcagtgttgccagtgcagctatgccagcagagatacctacaagctgaaacgccacatgagaacgcactcaggtgagaagccttacga
  E  R  P  F  Q  C  C  Q  C  S  Y  A  S  R  D  T  Y  K  L  K  R  H  M  R  T  H  S  G  E  K  P  Y  E  
atgccacatctgccacacccgcttcacccagagcgggaccatgaaaatacatattctgcagaaacacggcgaaaatgtccccaaataccagtgtcccca
  C  H  I  C  H  T  R  F  T  Q  S  G  T  M  K  I  H  I  L  Q  K  H  G  E  N  V  P  K  Y  Q  C  P  H  
ttgtgccaccatcattgcacggaaaagcgacctacgtgtgcatatgcgcaacttgcatgcttacagcgctgcagagctgaaatgccgctactgttctgc
  C  A  T  I  I  A  R  K  S  D  L  R  V  H  M  R  N  L  H  A  Y  S  A  A  E  L  K  C  R  Y  C  S  A  
tgtcttccatgaacgctatgccctcattcagcaccagaaaactcataagaatgagaagaggttcaagtgcaaacactgcagttatgcctgcaagcagga
  V  F  H  E  R  Y  A  L  I  Q  H  Q  K  T  H  K  N  E  K  R  F  K  C  K  H  C  S  Y  A  C  K  Q  E  
acgtcatatgaccgctcacattcgtacccacactggagagaaaccattcacctgcctttcttgcaataaatgtttccgacagaagcaacttctaaacgc
  R  H  M  T  A  H  I  R  T  H  T  G  E  K  P  F  T  C  L  S  C  N  K  C  F  R  Q  K  Q  L  L  N  A  
tcacttcaggaaataccacgatgcaaatttcatcccgactgtttacaaatgctccaagtgtggcaaaggcttttcccgctggattaacctgcacagaca
  H  F  R  K  Y  H  D  A  N  F  I  P  T  V  Y  K  C  S  K  C  G  K  G  F  S  R  W  I  N  L  H  R  H  
ttcggagaagtgtggatcaggggaagcaaagtcggctgcttcaggaaagggaagaagaacaagaaagaggaagcagaccatcctgaaggaagccacaaa
  S  E  K  C  G  S  G  E  A  K  S  A  A  S  G  K  G  R  R  T  R  K  R  K  Q  T  I  L  K  E  A  T  K  
gggtcagaaggaagctgcgaagggatggaaggaagccgcgaacggagacgaagctgctgctgaggaggcttccaccacgaagggagaacagttcccagg
  G  Q  K  E  A  A  K  G  W  K  E  A  A  N  G  D  E  A  A  A  E  E  A  S  T  T  K  G  E  Q  F  P  G  
agagatgtttcctgtcgcctgcagagaaaccacagccagagtcaaagaggaagtggatgaaggcgtgacctgtgaaatgctcctcaacacgatggataa
  E  M  F  P  V  A  C  R  E  T  T  A  R  V  K  E  E  V  D  E  G  V  T  C  E  M  L  L  N  T  M  D  K  
gtgagagggattcgggttgcgtgttcactgcccccaattcctaaagcaagttagaagtttttagcatttaaggtgtgaaatgctcctcaacacgatgga
  *
taagtgagagagagtcaggttgcatgttcactgcccctaattcctaaagcaagttagaaatttttagcattttctttgaaacaattaagttcatgacaatggatgacacaagtttgaggtagtgtctagaattgttctcctgtttgtagctggatatttcaaagaaacattgcaggtattttataaaagttttaaaccttgaatgagagggtaacacctcaaacctatggattcattcacttgatattggcaaggtggcccacaatgagtgagtagtgatttttggatatttcaaaatagtctagaccagctagtgcttccacagtcaaagctggacatttttatgttgcattatatacacccatgatatttctaataatatatggttttaaacattaaagacaaatgtttttatacaaatgaattttctacaaaatttaaagctaccataatgcttttaattagttctaaattcaaccaaaaaatgttttactcttataaaaaggaaaactgagtaggaaatgaaatactagattagactagaaaataaggaataaatcgattttactttggtataggagcaaggttcacctttagatttttgtattctcttttaattatgctccttggcaggtatgaaattgccctggttacattccattattgcttattagtatttcactccataacccttttttctgctaaaactactctttttatatttgtaaaataattggcagagtgagaagaaacataaaatcagataaggcaaatgtgtacctgtaaggaatttgtactttttcataatgcccagtgattagtgagtatttcccttttgccagttgacaagatttttccaccctcgagcagcgtgagagatgcctctttaacacttgaaattcatttctatctggatacagaggcagatttttcttcattgcttagttgagcagtttgttttgctgccaacctgtctccacccctgtatttcaagatcattgataagccctaaattcaaattcttaagatatggaccttttattgaaaatatcacaagttcagaatccctatacaatgtgaatatgtggaaataatttcccagcaggaagagcattatattctctttgtaccagcaaattaatttaactcaactcacatgagatttaaattctgtgggctgtagtatgccatcattgtgactgaatttgtgcaatggtttcttaatttttttactgttatttaaagatgttttacataattcaataaaatgaaatgacttaaaattgcaaaaaaaaaaaaaaaaaaaaaaaaaaaaaaaaaaaaa


Full-length nucleotide and deduced amino acid sequence of the BORIS A2 isoform (GenBank: DQ778109). BORIS A2 isoform is expressed from promoter A, contains eleven zinc fingers, N-terminus (N258), C-terminus (C95), and 3'UTR belonging to BORIS_sf1.

attccacccctccccccagtatctcagtgcctcctgtgggccctcctcccctccttatccattccccctcagatctcccaggccccctgcaggccctcgtgccctccttacttcccccccgggtctcccagcgccccctgcggggccctcctcccttcctcatccacttcaaccccaagcccggcggcggccggctgtgggctgcagcacgcggtgcacgaggcagagcccacaagccaaagacggagtgggccgagcattccggccacgccttccgcggccaagtcattatggcag
                                                                                             M  A     
ccactgagatctctgtcctttctgagcaattcaccaagatcaaagaactcgagttgatgccggaaaaaggcctgaaggaggaggaaaaagacggagtgt
A  T  E  I  S  V  L  S  E  Q  F  T  K  I  K  E  L  E  L  M  P  E  K  G  L  K  E  E  E  K  D  G  V  
Gcagagagaaagaccatcggagccctagtgagttggaggccgagcgtacctctggggccttccaggacagcgtcctggaggaagaagtggagctggtgc
C  R  E  K  D  H  R  S  P  S  E  L  E  A  E  R  T  S  G  A  F  Q  D  S  V  L  E  E  E  V  E  L  V  
Tggccccctcggaggagagcgagaagtacatcctgaccctgcagacggtgcacttcacttctgaagctgtggagttgcaggatatgagcttgctgagca
L  A  P  S  E  E  S  E  K  Y  I  L  T  L  Q  T  V  H  F  T  S  E  A  V  E  L  Q  D  M  S  L  L  S  
Tacagcagcaagaaggggtgcaggtggtggtgcaacagcctggccctgggttgctgtggcttgaggaagggccccggcagagcctgcagcagtgtgtgg
I  Q  Q  Q  E  G  V  Q  V  V  V  Q  Q  P  G  P  G  L  L  W  L  E  E  G  P  R  Q  S  L  Q  Q  C  V  
Ccattagtatccagcaagagctgtactccccgcaagagatggaggtgttgcagttccacgctctagaggagaatgtgatggtggccagtgaagacagta
A  I  S  I  Q  Q  E  L  Y  S  P  Q  E  M  E  V  L  Q  F  H  A  L  E  E  N  V  M  V  A  S  E  D  S  
Agttagcggtgagcctggctgaaactgctggactgatcaagctcgaggaagagcaggagaagaaccagttattggctgaaagaacaaaggagcagctct
K  L  A  V  S  L  A  E  T  A  G  L  I  K  L  E  E  E  Q  E  K  N  Q  L  L  A  E  R  T  K  E  Q  L  
Tttttgtggaaacaatgtcaggagatgaaagaagtgacgaaattgttctcacagtttcaaattcaaatgtggaagaacaagaggatcaacctacagctg
F  F  V  E  T  M  S  G  D  E  R  S  D  E  I  V  L  T  V  S  N  S  N  V  E  E  Q  E  D  Q  P  T  A  
Gtcaagcagatgctgaaaaggccaaatctacaaaaaatcaaagaaagacaaagggagcaaaaggaaccttccactgtgatgtctgcatgttcacctctt
G  Q  A  D  A  E  K  A  K  S  T  K  N  Q  R  K  T  K  G  A  K  G  T  F  H  C  D  V  C  M  F  T  S  
Ctagaatgtcaagttttaatcgtcatatgaaaactcacaccagtgagaagcctcacctgtgtcacctctgcctgaaaaccttccgtacggtcactctgc
S  R  M  S  S  F  N  R  H  M  K  T  H  T  S  E  K  P  H  L  C  H  L  C  L  K  T  F  R  T  V  T  L  
Tgcggaaccatgttaacacccacacaggaaccaggccctacaagtgtaacgactgcaacatggcatttgtcaccagtggagaactcgtccgacacaggc
L  R  N  H  V  N  T  H  T  G  T  R  P  Y  K  C  N  D  C  N  M  A  F  V  T  S  G  E  L  V  R  H  R  
Gctataaacatactcatgagaaaccctttaaatgttccatgtgcaagtatgccagtgtggaggcaagtaaattgaagcgccatgtccgatcccacactg
R  Y  K  H  T  H  E  K  P  F  K  C  S  M  C  K  Y  A  S  V  E  A  S  K  L  K  R  H  V  R  S  H  T  
Gggagcgcccctttcagtgttgccagtgcagctatgccagcagagatacctacaagctgaaacgccacatgagaacgcactcaggtgagaagccttacg
G  E  R  P  F  Q  C  C  Q  C  S  Y  A  S  R  D  T  Y  K  L  K  R  H  M  R  T  H  S  G  E  K  P  Y  
Aatgccacatctgccacacccgcttcacccagagcgggaccatgaaaatacatattctgcagaaacacggcgaaaatgtccccaaataccagtgtcccc
E  C  H  I  C  H  T  R  F  T  Q  S  G  T  M  K  I  H  I  L  Q  K  H  G  E  N  V  P  K  Y  Q  C  P  
Attgtgccaccatcattgcacggaaaagcgacctacgtgtgcatatgcgcaacttgcatgcttacagcgctgcagagctgaaatgccgctactgttctg
H  C  A  T  I  I  A  R  K  S  D  L  R  V  H  M  R  N  L  H  A  Y  S  A  A  E  L  K  C  R  Y  C  S  
Ctgtcttccatgaacgctatgccctcattcagcaccagaaaactcataagaatgagaagaggttcaagtgcaaacactgcagttatgcctgcaagcagg
A  V  F  H  E  R  Y  A  L  I  Q  H  Q  K  T  H  K  N  E  K  R  F  K  C  K  H  C  S  Y  A  C  K  Q  
Aacgtcatatgaccgctcacattcgtacccacactggagagaaaccattcacctgcctttcttgcaataaatgtttccgacagaagcaacttctaaacg
E  R  H  M  T  A  H  I  R  T  H  T  G  E  K  P  F  T  C  L  S  C  N  K  C  F  R  Q  K  Q  L  L  N  
Ctcacttcaggaaataccacgatgcaaatttcatcccgactgtttacaaatgctccaagtgtggcaaaggcttttcccgctggattaacctgcacagac
A  H  F  R  K  Y  H  D  A  N  F  I  P  T  V  Y  K  C  S  K  C  G  K  G  F  S  R  W  I  N  L  H  R  
Attcggagaagtgtggatcaggggaagcaaagtcggctgcttcaggaaagggaagaagaacaagaaagaggaagcagaccatcctgaaggaagccacaa
H  S  E  K  C  G  S  G  E  A  K  S  A  A  S  G  K  G  R  R  T  R  K  R  K  Q  T  I  L  K  E  A  T  
Agggtcagaaggaagctgcgaagggatggaaggaagccgcgaacggagacgaagctgctgctgaggaggcttccaccacgaagggagaacagttcccag
K  G  Q  K  E  A  A  K  G  W  K  E  A  A  N  G  D  E  A  A  A  E  E  A  S  T  T  K  G  E  Q  F  P  
Gagagatgtttcctgtcgcctgcagagaaaccacagccagagtcaaagaggaagtggatgaaggcgtgacctgtgaaatgctcctcaacacgatggata
G  E  M  F  P  V  A  C  R  E  T  T  A  R  V  K  E  E  V  D  E  G  V  T  C  E  M  L  L  N  T  M  D  
Agtgagagggattcgggttgcgtgttcactgcccccaattcctaaagcaagttagaagtttttagcatttaaggtgtgaaatgctcctcaacacgatgg
K  *
Ataagtgagagagagtcaggttgcatgttcactgcccctaattcctaaagcaagttagaaatttttagcattttctttgaaacaattaagttcatgacaatggatgacacaagtttgaggtagtgtctagaattgttctcctgtttgtagctggatatttcaaagaaacattgcaggtattttataaaagttttaaaccttgaatgagagggtaacacctcaaacctatggattcattcacttgatattggcaaggtggcccacaatgagtgagtagtgatttttggatatttcaaaatagtctagaccagctagtgcttccacagtcaaagctggacatttttatgttgcattatatacacccatgatatttctaataatatatggttttaaacattaaagacaaatgtttttatacaaatgaattttctacaaaatttaaagctaccataatgcttttaattagttctaaattcaaccaaaaaatgttttactcttataaaaaggaaaactgagtaggaaatgaaatactagattagactagaaaataaggaataaatcgattttactttggtataggagcaaggttcacctttagatttttgtattctcttttaattatgctccttggcaggtatgaaattgccctggttacattccattattgcttattagtatttcactccataacccttttttctgctaaaactactctttttatatttgtaaaataattggcagagtgagaagaaacataaaatcagataaggcaaatgtgtacctgtaaggaatttgtactttttcataatgcccagtgattagtgagtatttcccttttgccagttgacaagatttttccaccctcgagcagcgtgagagatgcctctttaacacttgaaattcatttctatctggatacagaggcagatttttcttcattgcttagttgagcagtttgttttgctgccaacctgtctccacccctgtatttcaagatcattgataagccctaaattcaaattcttaagatatggaccttttattgaaaatatcacaagttcagaatccctatacaatgtgaatatgtggaaataatttcccagcaggaagagcattatattctctttgtaccagcaaattaatttaactcaactcacatgagatttaaattctgtgggctgtagtatgccatcattgtgactgaatttgtgcaatggtttcttaatttttttactgttatttaaagatgttttacataattcaataaaatgaaatgacttaaaattgcaaaaaaaaaaaaaaaaaaaaaaaaaaaaaaaaaaaaa

 Full-length nucleotide and deduced amino acid sequence of the BORIS C1 isoform (GenBank: DQ778110).  BORIS C1 isoform is expressed from promoter C, contains eleven zinc fingers, N-terminus (N258), C-terminus (C95), and 3'UTR belonging to BORIS_sf1. 

agggtaaagcaggggccctgccaggcctccgagggagtgtgcttggtctggccgagggctgcttggccaagtctgggtgggctcgaggccactaggcccaaagcctgcctggctctgagggtgctaggtctagaaccgtgcacgaggggaatgcctgctcgggcccgaacctcgctgggcgccgggtgtgcactggcccggggcctgcttggacctgaaacttgctaggcccaggatatgcactggccgagagcctgctgggcccaaaccttactaggcccaggatgttcactgactgaaccggctcaggcctaaccttgctaggcccaggatatgcactgggccagagtgtgctcaggcggaaccttgccaggcgcaggatgtgtgctggccctaagcctgctgaggcccaaacctgttcgttctagggttttgtacaaaatcctgctttagcctaaatcctgcttagccttgaccccctcctagacccaagccagatcagcattgttctgaccctactaagtccaaaaccttttgaggccagaccttgtttcaactccaaagcctgctaggttccagcaccccccgcatccctcctcataccacccccttctcccccctatggaaaccgcttgcttatttttcaaacaggccaagtcattatggcagccactgagatctctgtcctttctg
                                                                     M  A  A  T  E  I  S  V  L  S  
agcaattcaccaagatcaaagaactcgagttgatgccggaaaaaggcctgaaggaggaggaaaaagacggagtgtgcagagagaaagaccatcggagcc  
E  Q  F  T  K  I  K  E  L  E  L  M  P  E  K  G  L  K  E  E  E  K  D  G  V  C  R  E  K  D  H  R  S  
ctagtgagttggaggccgagcgtacctctggggccttccaggacagcgtcctggaggaagaagtggagctggtgctggccccctcggaggagagcgaga
P  S  E  L  E  A  E  R  T  S  G  A  F  Q  D  S  V  L  E  E  E  V  E  L  V  L  A  P  S  E  E  S  E  
agtacatcctgaccctgcagacggtgcacttcacttctgaagctgtggagttgcaggatatgagcttgctgagcatacagcagcaagaaggggtgcagg
K  Y  I  L  T  L  Q  T  V  H  F  T  S  E  A  V  E  L  Q  D  M  S  L  L  S  I  Q  Q  Q  E  G  V  Q  
tggtggtgcaacagcctggccctgggttgctgtggcttgaggaagggccccggcagagcctgcagcagtgtgtggccattagtatccagcaagagctgt
V  V  V  Q  Q  P  G  P  G  L  L  W  L  E  E  G  P  R  Q  S  L  Q  Q  C  V  A  I  S  I  Q  Q  E  L  
actccccgcaagagatggaggtgttgcagttccacgctctagaggagaatgtgatggtggccagtgaagacagtaagttagcggtgagcctggctgaaa
Y  S  P  Q  E  M  E  V  L  Q  F  H  A  L  E  E  N  V  M  V  A  S  E  D  S  K  L  A  V  S  L  A  E  
ctgctggactgatcaagctcgaggaagagcaggagaagaaccagttattggctgaaagaacaaaggagcagctcttttttgtggaaacaatgtcaggag
T  A  G  L  I  K  L  E  E  E  Q  E  K  N  Q  L  L  A  E  R  T  K  E  Q  L  F  F  V  E  T  M  S  G  
atgaaagaagtgacgaaattgttctcacagtttcaaattcaaatgtggaagaacaagaggatcaacctacagctggtcaagcagatgctgaaaaggcca
D  E  R  S  D  E  I  V  L  T  V  S  N  S  N  V  E  E  Q  E  D  Q  P  T  A  G  Q  A  D  A  E  K  A  
aatctacaaaaaatcaaagaaagacaaagggagcaaaaggaaccttccactgtgatgtctgcatgttcacctcttctagaatgtcaagttttaatcgtc
K  S  T  K  N  Q  R  K  T  K  G  A  K  G  T  F  H  C  D  V  C  M  F  T  S  S  R  M  S  S  F  N  R  
atatgaaaactcacaccagtgagaagcctcacctgtgtcacctctgcctgaaaaccttccgtacggtcactctgctgcggaaccatgttaacacccaca
H  M  K  T  H  T  S  E  K  P  H  L  C  H  L  C  L  K  T  F  R  T  V  T  L  L  R  N  H  V  N  T  H  
caggaaccaggccctacaagtgtaacgactgcaacatggcatttgtcaccagtggagaactcgtccgacacaggcgctataaacatactcatgagaaac
T  G  T  R  P  Y  K  C  N  D  C  N  M  A  F  V  T  S  G  E  L  V  R  H  R  R  Y  K  H  T  H  E  K  
cctttaaatgttccatgtgcaagtatgccagtgtggaggcaagtaaattgaagcgccatgtccgatcccacactggggagcgcccctttcagtgttgcc
P  F  K  C  S  M  C  K  Y  A  S  V  E  A  S  K  L  K  R  H  V  R  S  H  T  G  E  R  P  F  Q  C  C  
agtgcagctatgccagcagagatacctacaagctgaaacgccacatgagaacgcactcaggtgagaagccttacgaatgccacatctgccacacccgct
Q  C  S  Y  A  S  R  D  T  Y  K  L  K  R  H  M  R  T  H  S  G  E  K  P  Y  E  C  H  I  C  H  T  R  
tcacccagagcgggaccatgaaaatacatattctgcagaaacacggcgaaaatgtccccaaataccagtgtccccattgtgccaccatcattgcacgga
F  T  Q  S  G  T  M  K  I  H  I  L  Q  K  H  G  E  N  V  P  K  Y  Q  C  P  H  C  A  T  I  I  A  R  
aaagcgacctacgtgtgcatatgcgcaacttgcatgcttacagcgctgcagagctgaaatgccgctactgttctgctgtcttccatgaacgctatgccc
K  S  D  L  R  V  H  M  R  N  L  H  A  Y  S  A  A  E  L  K  C  R  Y  C  S  A  V  F  H  E  R  Y  A  
tcattcagcaccagaaaactcataagaatgagaagaggttcaagtgcaaacactgcagttatgcctgcaagcaggaacgtcatatgaccgctcacattc
L  I  Q  H  Q  K  T  H  K  N  E  K  R  F  K  C  K  H  C  S  Y  A  C  K  Q  E  R  H  M  T  A  H  I  
gtacccacactggagagaaaccattcacctgcctttcttgcaataaatgtttccgacagaagcaacttctaaacgctcacttcaggaaataccacgatg
R  T  H  T  G  E  K  P  F  T  C  L  S  C  N  K  C  F  R  Q  K  Q  L  L  N  A  H  F  R  K  Y  H  D  
caaatttcatcccgactgtttacaaatgctccaagtgtggcaaaggcttttcccgctggattaacctgcacagacattcggagaagtgtggatcagggg
A  N  F  I  P  T  V  Y  K  C  S  K  C  G  K  G  F  S  R  W  I  N  L  H  R  H  S  E  K  C  G  S  G  
aagcaaagtcggctgcttcaggaaagggaagaagaacaagaaagaggaagcagaccatcctgaaggaagccacaaagggtcagaaggaagctgcgaagg
E  A  K  S  A  A  S  G  K  G  R  R  T  R  K  R  K  Q  T  I  L  K  E  A  T  K  G  Q  K  E  A  A  K  
gatggaaggaagccgcgaacggagacgaagctgctgctgaggaggcttccaccacgaagggagaacagttcccaggagagatgtttcctgtcgcctgca
G  W  K  E  A  A  N  G  D  E  A  A  A  E  E  A  S  T  T  K  G  E  Q  F  P  G  E  M  F  P  V  A  C  
gagaaaccacagccagagtcaaagaggaagtggatgaaggcgtgacctgtgaaatgctcctcaacacgatggataagtgagagggattcgggttgcgtg
R  E  T  T  A  R  V  K  E  E  V  D  E  G  V  T  C  E  M  L  L  N  T  M  D  K  *
ttcactgcccccaattcctaaagcaagttagaagtttttagcatttaaggtgtgaaatgctcctcaacacgatggataagtgagagagagtcaggttgc
atgttcactgcccctaattcctaaagcaagttagaaatttttagcattttctttgaaacaattaagttcatgacaatggatgacacaagtttgaggtagtgtctagaattgttctcctgtttgtagctggatatttcaaagaaacattgcaggtattttataaaagttttaaaccttgaatgagagggtaacacctcaaacctatggattcattcacttgatattggcaaggtggcccacaatgagtgagtagtgatttttggatatttcaaaatagtctagaccagctagtgcttccacagtcaaagctggacatttttatgttgcattatatacacccatgatatttctaataatatatggttttaaacattaaagacaaatgtttttatacaaatgaattttctacaaaatttaaagctaccataatgcttttaattagttctaaattcaaccaaaaaatgttttactcttataaaaaggaaaactgagtaggaaatgaaatactagattagactagaaaataaggaataaatcgattttactttggtataggagcaaggttcacctttagatttttgtattctcttttaattatgctccttggcaggtatgaaattgccctggttacattccattattgcttattagtatttcactccataacccttttttctgctaaaactactctttttatatttgtaaaataattggcagagtgagaagaaacataaaatcagataaggcaaatgtgtacctgtaaggaatttgtactttttcataatgcccagtgattagtgagtatttcccttttgccagttgacaagatttttccaccctcgagcagcgtgagagatgcctctttaacacttgaaattcatttctatctggatacagaggcagatttttcttcattgcttagttgagcagtttgttttgctgccaacctgtctccacccctgtatttcaagatcattgataagccctaaattcaaattcttaagatatggaccttttattgaaaatatcacaagttcagaatccctatacaatgtgaatatgtggaaataatttcccagcaggaagagcattatattctctttgtaccagcaaattaatttaactcaactcacatgagatttaaattctgtgggctgtagtatgccatcattgtgactgaatttgtgcaatggtttcttaatttttttactgttatttaaagatgttttacataattcaataaaatgaaatgacttaaaattgcaaaaaaaaaaaaaaaaaaaaaaaaaaaaaaaaaaaaa


Full-length nucleotide and deduced amino acid sequence of the BORIS A3 isoform (GenBank: DQ778112).  BORIS A3 isoform is expressed from promoter A, contains nine zinc fingers, N-terminus (N258), C-terminus (C95), and 3'UTR belonging to BORIS_sf1. 

ATTCCACCCCTCCCCCCAGTATCTCAGTGCCTCCTGTGGGCCCTCCTCCCCTCCTTATCCATTCCCCCTCAGATCTCCCAGGCCCCCTGCAGGCCCTCGTGCCCTCCTTACTTCCCCCCCGGGTCTCCCAGCGCCCCCTGCGGGGCCCTCCTCCCTTCCTCATCCACTTCAACCCCAAGGTATTTCCGTGCCCCTGCAGGACCCTCCTCCCCTCCTTAGGCGCTCCCCCACTACCCAGTCTTCCAGTGCCCTGCAGGCTCTCCTCCTCTCCTTATCCACCCCCACCCCAGGTCTCCCAGTGCCCTGTATGGGACCCTCCTCCCCTCCTCATCCACCCCCCCAGGTCCACCAGTGCCCCCTCTGGGGTCCTCCTCATCCGTGCTCCCCCTCCCCCTCCCTACTCCCCTTCCCCCCTGCCCCCACAGTACATCACCCCCTCCCCCAACCCTGCCTGGCTCCGCCCCCTTCACGCCCCCTCTTTTCCGCTCCGCGCCTGCGCACTGCCACCCTCCACTCTCGCGCCAGCCCGGCGGCGGCCGGCTGTGGGCTGCAGCACGCGGTGCACGAGGCAGAGCCCACAAGCCAAAGACGGAGTGGGCCGAGCATTCCGGCCACGCCTTCCGCGGCCAAGTCATTATGGCAGCCACTGAGATCTCTGTCCTTTCTGAGCAATTCACCAAGATCAAAGAACTC
                                            M  A  A  T  E  I  S  V  L  S  E  Q  F  T  K  I  K  E  L
GAGTTGATGCCGGAAAAAGGCCTGAAGGAGGAGGAAAAAGACGGAGTGTGCAGAGAGAAAGACCATCGGAGCCCTAGTGAGTTGGAGGCCGAGCGTACC
E  L  M  P  E  K  G  L  K  E  E  E  K  D  G  V  C  R  E  K  D  H  R  S  P  S  E  L  E  A  E  R  T
TCTGGGGCCTTCCAGGACAGCGTCCTGGAGGAAGAAGTGGAGCTGGTGCTGGCCCCCTCGGAGGAGAGCGAGAAGTACATCCTGACCCTGCAGACGGTG
S  G  A  F  Q  D  S  V  L  E  E  E  V  E  L  V  L  A  P  S  E  E  S  E  K  Y  I  L  T  L  Q  T  V
CACTTCACTTCTGAAGCTGTGGAGTTGCAGGATATGAGCTTGCTGAGCATACAGCAGCAAGAAGGGGTGCAGGTGGTGGTGCAACAGCCTGGCCCTGGG
H  F  T  S  E  A  V  E  L  Q  D  M  S  L  L  S  I  Q  Q  Q  E  G  V  Q  V  V  V  Q  Q  P  G  P  G
TTGCTGTGGCTTGAGGAAGGGCCCCGGCAGAGCCTGCAGCAGTGTGTGGCCATTAGTATCCAGCAAGAGCTGTACTCCCCGCAAGAGATGGAGGTGTTG
L  L  W  L  E  E  G  P  R  Q  S  L  Q  Q  C  V  A  I  S  I  Q  Q  E  L  Y  S  P  Q  E  M  E  V  L
CAGTTCCACGCTCTAGAGGAGAATGTGATGGTGGCCAGTGAAGACAGTAAGTTAGCGGTGAGCCTGGCTGAAACTGCTGGACTGATCAAGCTCGAGGAA
Q  F  H  A  L  E  E  N  V  M  V  A  S  E  D  S  K  L  A  V  S  L  A  E  T  A  G  L  I  K  L  E  E
GAGCAGGAGAAGAACCAGTTATTGGCTGAAAGAACAAAGGAGCAGCTCTTTTTTGTGGAAACAATGTCAGGAGATGAAAGAAGTGACGAAATTGTTCTC
E  Q  E  K  N  Q  L  L  A  E  R  T  K  E  Q  L  F  F  V  E  T  M  S  G  D  E  R  S  D  E  I  V  L 
ACAGTTTCAAATTCAAATGTGGAAGAACAAGAGGATCAACCTACAGCTGGTCAAGCAGATGCTGAAAAGGCCAAATCTACAAAAAATCAAAGAAAGACA
T  V  S  N  S  N  V  E  E  Q  E  D  Q  P  T  A  G  Q  A  D  A  E  K  A  K  S  T  K  N  Q  R  K  T 
AAGGGAGCAAAAGGAACCTTCCACTGTGATGTCTGCATGTTCACCTCTTCTAGAATGTCAAGTTTTAATCGTCATATGAAAACTCACACCAGTGAGAAG
K  G  A  K  G  T  F  H  C  D  V  C  M  F  T  S  S  R  M  S  S  F  N  R  H  M  K  T  H  T  S  E  K 
CCTCACCTGTGTCACCTCTGCCTGAAAACCTTCCGTACGGTCACTCTGCTGCGGAACCATGTTAACACCCACACAGGAACCAGGCCCTACAAGTGTAAC
P  H  L  C  H  L  C  L  K  T  F  R  T  V  T  L  L  R  N  H  V  N  T  H  T  G  T  R  P  Y  K  C  N 
GACTGCAACATGGCATTTGTCACCAGTGGAGAACTCGTCCGACACAGGCGCTATAAACATACTCATGAGAAACCCTTTAAATGTTCCATGTGCAAGTAT
D  C  N  M  A  F  V  T  S  G  E  L  V  R  H  R  R  Y  K  H  T  H  E  K  P  F  K  C  S  M  C  K  Y 
GCCAGTGTGGAGGCAAGTAAATTGAAGCGCCATGTCCGATCCCACACTGGGGAGCGCCCCTTTCAGTGTTGCCAGTGCAGCTATGCCAGCAGAGATACC
A  S  V  E  A  S  K  L  K  R  H  V  R  S  H  T  G  E  R  P  F  Q  C  C  Q  C  S  Y  A  S  R  D  T 
TACAAGCTGAAACGCCACATGAGAACGCACTCAGGTGTGCATATGCGCAACTTGCATGCTTACAGCGCTGCAGAGCTGAAATGCCGCTACTGTTCTGCT
Y  K  L  K  R  H  M  R  T  H  S  G  V  H  M  R  N  L  H  A  Y  S  A  A  E  L  K  C  R  Y  C  S  A
GTCTTCCATGAACGCTATGCCCTCATTCAGCACCAGAAAACTCATAAGAATGAGAAGAGGTTCAAGTGCAAACACTGCAGTTATGCCTGCAAGCAGGAA
V  F  H  E  R  Y  A  L  I  Q  H  Q  K  T  H  K  N  E  K  R  F  K  C  K  H  C  S  Y  A  C  K  Q  E 
CGTCATATGACCGCTCACATTCGTACCCACACTGGAGAGAAACCATTCACCTGCCTTTCTTGCAATAAATGTTTCCGACAGAAGCAACTTCTAAACGCT
R  H  M  T  A  H  I  R  T  H  T  G  E  K  P  F  T  C  L  S  C  N  K  C  F  R  Q  K  Q  L  L  N  A 
CACTTCAGGAAATACCACGATGCAAATTTCATCCCGACTGTTTACAAATGCTCCAAGTGTGGCAAAGGCTTTTCCCGCTGGATTAACCTGCACAGACAT
H  F  R  K  Y  H  D  A  N  F  I  P  T  V  Y  K  C  S  K  C  G  K  G  F  S  R  W  I  N  L  H  R  H  
TCGGAGAAGTGTGGATCAGGGGAAGCAAAGTCGGCTGCTTCAGGAAAGGGAAGAAGAACAAGAAAGAGGAAGCAGACCATCCTGAAGGAAGCCACAAAG
S  E  K  C  G  S  G  E  A  K  S  A  A  S  G  K  G  R  R  T  R  K  R  K  Q  T  I  L  K  E  A  T  K  
GGTCAGAAGGAAGCTGCGAAGGGATGGAAGGAAGCCGCGAACGGAGACGAAGCTGCTGCTGAGGAGGCTTCCACCACGAAGGGAGAACAGTTCCCAGGA
G  Q  K  E  A  A  K  G  W  K  E  A  A  N  G  D  E  A  A  A  E  E  A  S  T  T  K  G  E  Q  F  P  G  
GAGATGTTTCCTGTCGCCTGCAGAGAAACCACAGCCAGAGTCAAAGAGGAAGTGGATGAAGGCGTGACCTGTGAAATGCTCCTCAACACGATGGATAAG
E  M  F  P  V  A  C  R  E  T  T  A  R  V  K  E  E  V  D  E  G  V  T  C  E  M  L  L  N  T  M  D  K  
TGAGAGGGATTCGGGTTGCGTGTTCACTGCCCCCAATTCCTAAAGCAAGTTAGAAGTTTTTAGCATTTAAGGTGTGAAATGCTCCTCAACACGATGGAT
 *
AAGTGAGAGAGAGTCAGGTTGCATGTTCACTGCCCCTAATTCCTAAAGCAAGTTAGAAATTTTTAGCATTTTCTTTGAAACAATTAAGTTCATGACAATGGATGACACAAGTTTGAGGTAGTGTCTAGAATTGTTCTCCTGTTTGTAGCTGGATATTTCAAAGAAACATTGCAGGTATTTTATAAAAGTTTTAAACCTTGAATGAGAGGGTAACACCTCAAACCTATGGATTCATTCACTTGATATTGGCAAGGTGGCCCACAATGAGTGAGTAGTGATTTTTGGATATTTCAAAATAGTCTAGACCAGCTAGTGCTTCCACAGTCAAAGCTGGACATTTTTATGTTGCATTATATACACCCATGATATTTCTAATAATATATGGTTTTAAACATTAAAGACAAATGTTTTTATACAAATGAATTTTCTACAAAATTTAAAGCTACCATAATGCTTTTAATTAGTTCTAAATTCAACCAAAAAATGTTTTACTCTTATAAAAAGGAAAACTGAGTAGGAAATGAAATACTAGATTAGACTAGAAAATAAGGAATAAATCGATTTTACTTTGGTATAGGAGCAAGGTTCACCTTTAGATTTTTGTATTCTCTTTTAATTATGCTCCTTGGCAGGTATGAAATTGCCCTGGTTACATTCCATTATTGCTTATTAGTATTTCACTCCATAACCCTTTTTTCTGCTAAAACTACTCTTTTTATATTTGTAAAATAATTGGCAGAGTGAGAAGAAACATAAAATCAGATAAGGCAAATGTGTACCTGTAAGGAATTTGTACTTTTTCATAATGCCCAGTGATTAGTGAGTATTTCCCTTTTGCCAGTTGACAAGATTTTTCCACCCTCGAGCAGCGTGAGAGATGCCTCTTTAACACTTGAAATTCATTTCTATCTGGATACAGAGGCAGATTTTTCTTCATTGCTTAGTTGAGCAGTTTGTTTTGCTGCCAACCTGTCTCCACCCCTGTATTTCAAGATCATTGATAAGCCCTAAATTCAAATTCTTAAGATATGGACCTTTTATTGAAAATATCACAAGTTCAGAATCCCTATACAATGTGAATATGTGGAAATAATTTCCCAGCAGGAAGAGCATTATATTCTCTTTGTACCAGCAAATTAATTTAACTCAACTCACATGAGATTTAAATTCTGTGGGCTGTAGTATGCCATCATTGTGACTGAATTTGTGCAATGGTTTCTTAATTTTTTTACTGTTATTTAAAGATGTTTTACATAATTCAATAAAATGAAATGACTTAAAATTGCAAAAAAAAAAAAAAAAAAAAAAAAAAAAAAAAAAAAA

 Full-length nucleotide and deduced amino acid sequence of the BORIS B1 isoform (GenBank: DQ778111).  BORIS B1 isoform is expressed from promoter B, contains eleven zinc fingers, N-terminus (N258), C-terminus (C132), and 3'UTR belonging to BORIS subfamily 5 (BORIS_sf5). 

ggcaccagacgcggtgcacgaggcagagccacaagccaaagacggagtgggccgagcattccggccacgccttccgcggccaagtcattatggcagcca
                                                                                          M  A  A  ctgagatctctgtcctttctgagcaattcaccaagatcaaagaactcgagttgatgccggaaaaaggcctgaaggaggaggaaaaagacggagtgtgca
T  E  I  S  V  L  S  E  Q  F  T  K  I  K  E  L  E  L  M  P  E  K  G  L  K  E  E  E  K  D  G  V  C
gagagaaagaccatcggagccctagtgagttggaggccgagcgtacctctggggccttccaggacagcgtcctggaggaagaagtggagctggtgctgg
  R  E  K  D  H  R  S  P  S  E  L  E  A  E  R  T  S  G  A  F  Q  D  S  V  L  E  E  E  V  E  L  V  L  
ccccctcggaggagagcgagaagtacatcctgaccctgcagacggtgcacttcacttctgaagctgtggagttgcaggatatgagcttgctgagcatac
A  P  S  E  E  S  E  K  Y  I  L  T  L  Q  T  V  H  F  T  S  E  A  V  E  L  Q  D  M  S  L  L  S  I  
agcagcaagaaggggtgcaggtggtggtgcaacagcctggccctgggttgctgtggcttgaggaagggccccggcagagcctgcagcagtgtgtggcca
Q  Q  Q  E  G  V  Q  V  V  V  Q  Q  P  G  P  G  L  L  W  L  E  E  G  P  R  Q  S  L  Q  Q  C  V  A   
ttagtatccagcaagagctgtactccccgcaagagatggaggtgttgcagttccacgctctagaggagaatgtgatggtggccagtgaagacagtaagt
I  S  I  Q  Q  E  L  Y  S  P  Q  E  M  E  V  L  Q  F  H  A  L  E  E  N  V  M  V  A  S  E  D  S  K   
tagcggtgagcctggctgaaactactggactgatcaagctcgaggaagagcaggagaagaaccagttattggctgaaagaacaaaggagcagctctttt
L  A  V  S  L  A  E  T  T  G  L  I  K  L  E  E  E  Q  E  K  N  Q  L  L  A  E  R  T  K  E  Q  L  F   
ttgtggaaacaatgtcaggagatgaaagaagtgacgaaattgttctcacagtttcaaattcaaatgtggaagaacaagaggatcaacctacagctggtc
F  V  E  T  M  S  G  D  E  R  S  D  E  I  V  L  T  V  S  N  S  N  V  E  E  Q  E  D  Q  P  T  A  G   
aagcagatgctgaaaaggccaaatctacaaaaaatcaaagaaagacaaagggagcaaaaggaaccttccactgtgatgtctgcatgttcacctcttcta
Q  A  D  A  E  K  A  K  S  T  K  N  Q  R  K  T  K  G  A  K  G  T  F  H  C  D  V  C  M  F  T  S  S    
gaatgtcaagttttaatcgtcatatgaaaactcacaccagtgagaagcctcacctgtgtcacctctgcctgaaaaccttccgtacggtcactctgctgc
R  M  S  S  F  N  R  H  M  K  T  H  T  S  E  K  P  H  L  C  H  L  C  L  K  T  F  R  T  V  T  L  L   
ggaaccatgttaacacccacacaggaaccaggccctacaagtgtaacgactgcaacatggcatttgtcaccagtggagaactcgtccgacacaggcgct
R  N  H  V  N  T  H  T  G  T  R  P  Y  K  C  N  D  C  N  M  A  F  V  T  S  G  E  L  V  R  H  R  R   
ataaacatactcatgagaaaccctttaaatgttccatgtgcaagtatgccagtgtggaggcaagtaaattgaagcgccatgtccgatcccacactgggg
Y  K  H  T  H  E  K  P  F  K  C  S  M  C  K  Y  A  S  V  E  A  S  K  L  K  R  H  V  R  S  H  T  G   
agcgcccctttcagtgttgccagtgcagctatgccagcagagatacctacaagctgaaacgccacatgagaacgcactcaggtgagaagccttacgaat
E  R  P  F  Q  C  C  Q  C  S  Y  A  S  R  D  T  Y  K  L  K  R  H  M  R  T  H  S  G  E  K  P  Y  E  
gccacatctgccacacccgcttcacccagagcgggaccatgaaaatacatattctgcagaaacacggcgaaaatgtccccaaataccagtgtccccatt
C  H  I  C  H  T  R  F  T  Q  S  G  T  M  K  I  H  I  L  Q  K  H  G  E  N  V  P  K  Y  Q  C  P  H   
gtgccaccatcattgcacggaaaagcgacctacgtgtgcatatgcgcaacttgcatgcttacagcgctgcagagctgaaatgccgctactgttctgctg
C  A  T  I  I  A  R  K  S  D  L  R  V  H  M  R  N  L  H  A  Y  S  A  A  E  L  K  C  R  Y  C  S  A   
tcttccatgaacgctatgccctcattcagcaccagaaaactcataagaatgagaagaggttcaagtgcaaacactgcagttatgcctgcaagcaggaac
V  F  H  E  R  Y  A  L  I  Q  H  Q  K  T  H  K  N  E  K  R  F  K  C  K  H  C  S  Y  A  C  K  Q  E    
gtcatatgaccgctcacattcgtacccacactggagagaaaccattcacctgcctttcttgcaataaatgtttccgacagaagcaacttctaaacgctc
R  H  M  T  A  H  I  R  T  H  T  G  E  K  P  F  T  C  L  S  C  N  K  C  F  R  Q  K  Q  L  L  N  A    
acttcaggaaataccacgatgcaaatttcatcccgactgtttacaaatgctccaagtgtggcaaaggcttttcccgctggattaacctgcacagacatt
H  F  R  K  Y  H  D  A  N  F  I  P  T  V  Y  K  C  S  K  C  G  K  G  F  S  R  W  I  N  L  H  R  H    
cggagaagtgtggatcaggggaagcaaagtcggctgcttcaggaaagggaagaagaacaagaaagaggaagcagaccatcctgaaggaagccacaaagg
S  E  K  C  G  S  G  E  A  K  S  A  A  S  G  K  G  R  R  T  R  K  R  K  Q  T  I  L  K  E  A  T  K    
gtcagaaggaagctgcgaagggatggaaggaagccgcgaacggagacgaagctgctgctgaggaggcttccaccacgaagggagaacagttcccaggag
G  Q  K  E  A  A  K  G  W  K  E  A  A  N  G  D  E  A  A  A  E  E  A  S  T  T  K  G  E  Q  F  P  G    
agatgtttcctgtcgcctgcagagaaaccacagccagagtcaaagaggaagtggatgaaggcgtgacctgtgaaatgctcctcaacacgatggataatt
E  M  F  P  V  A  C  R  E  T  T  A  R  V  K  E  E  V  D  E  G  V  T  C  E  M  L  L  N  T  M  D  N    
ccgcaggctgtacaggaaggatgatgttggtatctgcctggcttctggggaggcctcaggaaacttacaatcaaggcagaaggcgaagagggagtaggc
S  A  G  C  T  G  R  M  M  L  V  S  A  W  L  L  G  R  P  Q  E  T  Y  N  Q  G  R  R  R  R  G  S  R    
gcgtcacatggtgaaagcaggagcgagagagggggaggcgccacactcctctgaacgaccagattccttgagaaccgccactgtcatgaggacagcacc
R  V  T  W  *
aagcggatggtgctaaatcattcctaagaaaccgcccccgtgatccagtcacctcctaccaggccccacctccaacactggggattacaattcaacatg
agctttggccagggacaaatatccaaactatatcatgtattttactacaaccaaatgtatgttaatttcaaaaacagataacataaatatgtttgaaaa
cgtgaaaaaaaaaaaaaaaaaaaaaaaaaaa


Full-length nucleotide and deduced amino acid sequence of the BORIS A4 isoform (GenBank: DQ778113).  BORIS A4 isoform is expressed from promoter A, contains three zinc fingers, N-terminus (N258), C-terminus (C69), and 3'UTR belonging to BORIS subfamily 2 (BORIS_sf2). 

attccacccctccccccagtatctcagtgcctcctgtgggccctcctcccctccttatccattccccctcagatctcccaggccccctgcaggccctcg
tgccctccttacttcccccccgggtctcccagcgccccctgcggggccctcctcccttcctcatccacttcaaccccaaggccaagtcattatggcagc
                                                                                            M  A  A  
cactgagatctctgtcctttctgagcaattcaccaagatcaaagaactcgagttgatgccggaaaaaggcctgaaggaggaggaaaaagacggagtgtg
  T  E  I  S  V  L  S  E  Q  F  T  K  I  K  E  L  E  L  M  P  E  K  G  L  K  E  E  E  K  D  G  V  C
cagagagaaagaccatcggagccctagtgagttggaggccgagcgtacctctggggccttccaggacagcgtcctggaggaagaagtggagctggtgct
  R  E  K  D  H  R  S  P  S  E  L  E  A  E  R  T  S  G  A  F  Q  D  S  V  L  E  E  E  V  E  L  V  L
ggccccctcggaggagagcgagaagtacatcctgaccctgcagacggtgcacttcacttctgaagctgtggagttgcaggatatgagcttgctgagcat
  A  P  S  E  E  S  E  K  Y  I  L  T  L  Q  T  V  H  F  T  S  E  A  V  E  L  Q  D  M  S  L  L  S  I 
acagcagcaagaaggggtgcaggtggtggtgcaacagcctggccctgggttgctgtggcttgaggaagggccccggcagagcctgcagcagtgtgtggc
  Q  Q  Q  E  G  V  Q  V  V  V  Q  Q  P  G  P  G  L  L  W  L  E  E  G  P  R  Q  S  L  Q  Q  C  V  A  cattagtatccagcaagagctgtactccccgcaagagatggaggtgttgcagttccacgctctagaggagaatgtgatggtggccagtgaagacagtaa
  I  S  I  Q  Q  E  L  Y  S  P  Q  E  M  E  V  L  Q  F  H  A  L  E  E  N  V  M  V  A  S  E  D  S  K
gttagcggtgagcctggctgaaactgctggactgatcaagctcgaggaagagcaggagaagaaccagttattggctgaaagaacaaaggagcagctctt
  L  A  V  S  L  A  E  T  A  G  L  I  K  L  E  E  E  Q  E  K  N  Q  L  L  A  E  R  T  K  E  Q  L  F
ttttgtggaaacaatgtcaggagatgaaagaagtgacgaaattgttctcacagtttcaaattcaaatgtggaagaacaagaggatcaacctacagctgg
  F  V  E  T  M  S  G  D  E  R  S  D  E  I  V  L  T  V  S  N  S  N  V  E  E  Q  E  D  Q  P  T  A  G 
tcaagcagatgctgaaaaggccaaatctacaaaaaatcaaagaaagacaaagggagcaaaaggaaccttccactgtgatgtctgcatgttcacctcttc
  Q  A  D  A  E  K  A  K  S  T  K  N  Q  R  K  T  K  G  A  K  G  T  F  H  C  D  V  C  M  F  T  S  S 
tagaatgtcaagttttaatcgtcatatgaaaactcacaccagtgagaagcctcacctgtgtcacctctgcctgaaaaccttccgtacggtcactctgct
  R  M  S  S  F  N  R  H  M  K  T  H  T  S  E  K  P  H  L  C  H  L  C  L  K  T  F  R  T  V  T  L  L  
gcggaaccatgttaacacccacacaggaaccaggccctacaagtgtaacgactgcaacatggcatttgtcaccagtggagaactcgtccgacacaggcg
  R  N  H  V  N  T  H  T  G  T  R  P  Y  K  C  N  D  C  N  M  A  F  V  T  S  G  E  L  V  R  H  R  R 
ctataaacatactcatgagaaaccctttaaatgttccatgtgcaagtatgccagtgtggaggtaaagccattcttggacttgaagcttcatggcatctt
  Y  K  H  T  H  E  K  P  F  K  C  S  M  C  K  Y  A  S  V  E  V  K  P  F  L  D  L  K  L  H  G  I  L  
agtagaggctgctgtacaagttactccaagtgtaactaacagtagaatctgttacaaacaggctttttattattcatataaaatttatgcaggaaataa
  V  E  A  A  V  Q  V  T  P  S  V  T  N  S  R  I  C  Y  K  Q  A  F  Y  Y  S  Y  K  I  Y  A  G  N  N  tatgcattctcttttatgaagttaaaatagaaaattggatttctttgtttctttgattactaattagtgaaaataatatgtaaaggtttacaaattaca
  M  H  S  L  L  *
aataaaccatctgtggttaaaaaaaaaaaaaaaaaaaaaaaaaa


Full-length nucleotide and deduced amino acid sequence of the BORIS C2 isoform (GenBank: DQ778114).   BORIS C2 isoform is expressed from promoter C, contains three zinc fingers, N-terminus (N258), C-terminus (C69), and 3'UTR belonging to BORIS subfamily 2 (BORIS_sf2).


agggtaaagcaggggccctgccaggcctccgagggagtgtgcttggtctggccgagggctgcttggccaagtctgggtgggctcgaggccactaggcccaaagcctgcctggctctgagggtgctaggtctagaaccgtgcacgaggggaatgcctgctcgggcccgaacctcgctgggcgccgggtgtgcactggcccggggcctgcttggacctgaaacttgctaggcccaggatatgcactggccgagagcctgctgggcccaaaccttactaggcccaggatgttcactgactgaaccggctcaggcctaaccttgctaggcccaggatatgcactgggccagagtgtgctcaggcggaaccttgccaggcgcaggatgtgtgctggccctaagcctgctgaggcccaaacctgttcgttctagggttttgtacaaaatcctgctttagcctaaatcctgcttagccttgaccccctcctagacccaagccagatcagcattgttctgaccctactaagtccaaaaccttttgaggccagaccttgtttcaactccaaagcctgctaggttccagcaccccccgcatccc

tcctcataccacccccttctcccccctatggaaaccgcttgcttatttttcaaacaggccaagtcattatggcagccactgagatctctgtcctttctg
                                                                     M  A  A  T  E  I  S  V  L  S    
agcaattcaccaagatcaaagaactcgagttgatgccggaaaaaggcctgaaggaggaggaaaaagacggagtgtgcagagagaaagaccatcggagcc
E  Q  F  T  K  I  K  E  L  E  L  M  P  E  K  G  L  K  E  E  E  K  D  G  V  C  R  E  K  D  H  R  S  
ctagtgagttggaggccgagcgtacctctggggccttccaggacagcgtcctggaggaagaagtggagctggtgctggccccctcggaggagagcgaga
P  S  E  L  E  A  E  R  T  S  G  A  F  Q  D  S  V  L  E  E  E  V  E  L  V  L  A  P  S  E  E  S  E  
agtacatcctgaccctgcagacggtgcacttcacttctgaagctgtggagttgcaggatatgagcttgctgagcatacagcagcaagaaggggtgcagg
K  Y  I  L  T  L  Q  T  V  H  F  T  S  E  A  V  E  L  Q  D  M  S  L  L  S  I  Q  Q  Q  E  G  V  Q 
tggtggtgcaacagcctggccctgggttgctgtggcttgaggaagggccccggcagagcctgcagcagtgtgtggccattagtatccagcaagagctgt
V  V  V  Q  Q  P  G  P  G  L  L  W  L  E  E  G  P  R  Q  S  L  Q  Q  C  V  A  I  S  I  Q  Q  E  L 
actccccgcaagagatggaggtgttgcagttccacgctctagaggagaatgtgatggtggccagtgaagacagtaagttagcggtgagcctggctgaaa
Y  S  P  Q  E  M  E  V  L  Q  F  H  A  L  E  E  N  V  M  V  A  S  E  D  S  K  L  A  V  S  L  A  E 
ctgctggactgatcaagctcgaggaagagcaggagaagaaccagttattggctgaaagaacaaaggagcagctcttttttgtggaaacaatgtcaggag
T  A  G  L  I  K  L  E  E  E  Q  E  K  N  Q  L  L  A  E  R  T  K  E  Q  L  F  F  V  E  T  M  S  G 
atgaaagaagtgacgaaattgttctcacagtttcaaattcaaatgtggaagaacaagaggatcaacctacagctggtcaagcagatgctgaaaaggcca
D  E  R  S  D  E  I  V  L  T  V  S  N  S  N  V  E  E  Q  E  D  Q  P  T  A  G  Q  A  D  A  E  K  A 
aatctacaaaaaatcaaagaaagacaaagggagcaaaaggaaccttccactgtgatgtctgcatgttcacctcttctagaatgtcaagttttaatcgtc
K  S  T  K  N  Q  R  K  T  K  G  A  K  G  T  F  H  C  D  V  C  M  F  T  S  S  R  M  S  S  F  N  R 
atatgaaaactcacaccagtgagaagcctcacctgtgtcacctctgcctgaaaaccttccgtacggtcactctgctgcggaaccatgttaacacccaca
H  M  K  T  H  T  S  E  K  P  H  L  C  H  L  C  L  K  T  F  R  T  V  T  L  L  R  N  H  V  N  T  H  
caggaaccaggccctacaagtgtaacgactgcaacatggcatttgtcaccagtggagaactcgtccgacacaggcgctataaacatactcatgagaaac
T  G  T  R  P  Y  K  C  N  D  C  N  M  A  F  V  T  S  G  E  L  V  R  H  R  R  Y  K  H  T  H  E  K 
cctttaaatgttccatgtgcaagtatgccagtgtggaggtaaagccattcttggacttgaagcttcatggcatcttagtagaggctgctgtacaagtta
P  F  K  C  S  M  C  K  Y  A  S  V  E  V  K  P  F  L  D  L  K  L  H  G  I  L  V  E  A  A  V  Q  V 
ctccaagtgtaactaacagtagaatctgttacaaacaggctttttattattcatataaaatttatgcaggaaataatatgcattctcttttatgaagtt
T  P  S  V  T  N  S  R  I  C  Y  K  Q  A  F  Y  Y  S  Y  K  I  Y  A  G  N  N  M  H  S  L  L  *
aaaatagaaaattggatttctttgtttctttgattactaattagtgaaaataatatgtaaaggtttacaaattacaaataaaccatctgtggttaaaaaaaaaaaaaaaaaaaaaaaaaa


 Full-length nucleotide and deduced amino acid sequence of the BORIS C3 isoform (GenBank: DQ778115).  BORIS C3 isoform is expressed from promoter C, contains eleven zinc fingers, N-terminus (N258), C-terminus (C97), and 3'UTR belonging to BORIS subfamily 4 (BORIS_sf4). 


agggtaaagcaggggccctgccaggcctccgagggagtgtgcttggtctggccgagggctgcttggccaagtctgggtgggctcgaggccactaggcccaaagcctgcctggctctgagggtgctaggtctagaaccgtgcacgaggggaatgcctgctcgggcccgaacctcgctgggcgccgggtgtgcactggcccggggcctgcttggacctgaaacttgctaggcccaggatatgcactggccgagagcctgctgggcccaaaccttactaggcccaggatgttcactgactgaaccggctcaggcctaaccttgctaggcccaggatatgcactgggccagagtgtgctcaggcggaaccttgccaggcgcaggatgtgtgctggccctaagcctgctgaggcccaaacctgttcgttctagggttttgtacaaaatcctgctttagcctaaatcctgcttagccttgaccccctcctagacccaagccagatcagcattgttctgaccctactaagtccaaaaccttttgaggccagaccttgtttcaactccaaagcctgctaggttccagcaccccccgcatccctcctcataccacccccttctcccccctatggaaaccgcttgcttatttttcaaacaggccaagtcattatggcagccactgagatctctgtcctttctg
                                                                     M  A  A  T  E  I  S  V  L  S  
agcaattcaccaagatcaaagaactcgagttgatgccggaaaaaggcctgaaggaggaggaaaaagacggagtgtgcagagagaaagaccatcggagcc
E  Q  F  T  K  I  K  E  L  E  L  M  P  E  K  G  L  K  E  E  E  K  D  G  V  C  R  E  K  D  H  R  S 
ctagtgagttggaggccgagcgtacctctggggccttccaggacagcgtcctggaggaagaagtggagctggtgctggccccctcggaggagagcgaga
P  S  E  L  E  A  E  R  T  S  G  A  F  Q  D  S  V  L  E  E  E  V  E  L  V  L  A  P  S  E  E  S  E
agtacatcctgaccctgcagacggtgcacttcacttctgaagctgtggagttgcaggatatgagcttgctgagcatacagcagcaagaaggggtgcagg
K  Y  I  L  T  L  Q  T  V  H  F  T  S  E  A  V  E  L  Q  D  M  S  L  L  S  I  Q  Q  Q  E  G  V  Q  tggtggtgcaacagcctggccctgggttgctgtggcttgaggaagggccccggcagagcctgcagcagtgtgtggccattagtatccagcaagagctgt
V  V  V  Q  Q  P  G  P  G  L  L  W  L  E  E  G  P  R  Q  S  L  Q  Q  C  V  A  I  S  I  Q  Q  E  L 
actccccgcaagagatggaggtgttgcagttccacgctctagaggagaatgtgatggtggccagtgaagacagtaagttagcggtgagcctggctgaaa
Y  S  P  Q  E  M  E  V  L  Q  F  H  A  L  E  E  N  V  M  V  A  S  E  D  S  K  L  A  V  S  L  A  E 
ctactggactgatcaagctcgaggaagagcaggagaagaaccagttattggctgaaagaacaaaggagcagctcttttttgtggaaacaatgtcaggag
T  T  G  L  I  K  L  E  E  E  Q  E  K  N  Q  L  L  A  E  R  T  K  E  Q  L  F  F  V  E  T  M  S  G 
atgaaagaagtgacgaaattgttctcacagtttcaaattcaaatgtggaagaacaagaggatcaacctacagctggtcaagcagatgctgaaaaggcca
D  E  R  S  D  E  I  V  L  T  V  S  N  S  N  V  E  E  Q  E  D  Q  P  T  A  G  Q  A  D  A  E  K  A 
aatctacaaaaaatcaaagaaagacaaagggagcaaaaggaaccttccactgtgatgtctgcatgttcacctcttctagaatgtcaagttttaatcgtc
K  S  T  K  N  Q  R  K  T  K  G  A  K  G  T  F  H  C  D  V  C  M  F  T  S  S  R  M  S  S  F  N  R 
atatgaaaactcacaccagtgagaagcctcacctgtgtcacctctgcctgaaaaccttccgtacggtcactctgctgcggaaccatgttaacacccaca
H  M  K  T  H  T  S  E  K  P  H  L  C  H  L  C  L  K  T  F  R  T  V  T  L  L  R  N  H  V  N  T  H 
caggaaccaggccctacaagtgtaacgactgcaacatggcatttgtcaccagtggagaactcgtccgacacaggcgctataaacatactcatgagaaac
T  G  T  R  P  Y  K  C  N  D  C  N  M  A  F  V  T  S  G  E  L  V  R  H  R  R  Y  K  H  T  H  E  K  
cctttaaatgttccatgtgcaagtatgccagtgtggaggcaagtaaattgaagcgccatgtccgatcccacactggggagcgcccctttcagtgttgcc
P  F  K  C  S  M  C  K  Y  A  S  V  E  A  S  K  L  K  R  H  V  R  S  H  T  G  E  R  P  F  Q  C  C  
agtgcagctatgccagcagagatacctacaagctgaaacgccacatgagaacgcactcaggtgagaagccttacgaatgccacatctgccacacccgct
Q  C  S  Y  A  S  R  D  T  Y  K  L  K  R  H  M  R  T  H  S  G  E  K  P  Y  E  C  H  I  C  H  T  R  
tcacccagagcgggaccatgaaaatacatattctgcagaaacacggcgaaaatgtccccaaataccagtgtccccattgtgccaccatcattgcacgga
F  T  Q  S  G  T  M  K  I  H  I  L  Q  K  H  G  E  N  V  P  K  Y  Q  C  P  H  C  A  T  I  I  A  R  aaagcgacctacgtgtgcatatgcgcaacttgcatgcttacagcgctgcagagctgaaatgccgctactgttctgctgtcttccatgaacgctatgccc
K  S  D  L  R  V  H  M  R  N  L  H  A  Y  S  A  A  E  L  K  C  R  Y  C  S  A  V  F  H  E  R  Y  A  
tcattcagcaccagaaaactcataagaatgagaagaggttcaagtgcaaacactgcagttatgcctgcaagcaggaacgtcatatgaccgctcacattc
L  I  Q  H  Q  K  T  H  K  N  E  K  R  F  K  C  K  H  C  S  Y  A  C  K  Q  E  R  H  M  T  A  H  I 
gtacccacactggagagaaaccattcacctgcctttcttgcaataaatgtttccgacagaagcaacttctaaacgctcacttcaggaaataccacgatg
R  T  H  T  G  E  K  P  F  T  C  L  S  C  N  K  C  F  R  Q  K  Q  L  L  N  A  H  F  R  K  Y  H  D caaatttcatcccgactgtttacaaatgctccaagtgtggcaaaggcttttcccgctggattaacctgcacagacattcggagaagtgtggatcagggg
A  N  F  I  P  T  V  Y  K  C  S  K  C  G  K  G  F  S  R  W  I  N  L  H  R  H  S  E  K  C  G  S  G 
aagcaaagtcggctgcttcaggaaagggaagaagaacaagaaagaggaagcagaccatcctgaaggaagccacaaagggtcagaaggaagctgcgaagg
E  A  K  S  A  A  S  G  K  G  R  R  T  R  K  R  K  Q  T  I  L  K  E  A  T  K  G  Q  K  E  A  A  K 
gatggaaggaagccgcgaacggagacggtgtgatctcagctcaccgcaacctctgcctcctgggttcaagtgattctcatgcctcagtctccggagctg
G  W  K  E  A  A  N  G  D  G  V  I  S  A  H  R  N  L  C  L  L  G  S  S  D  S  H  A  S  V  S  G  A 
ggattacagatgcccgccaccacgcctggctaattgttctattatttttagtagagatggggttttaccatgtctctcactcctgacctcaagtgatct
G  I  T  D  A  R  H  H  A  W  L  I  V  L  L  F  L  V  E  M  G  F  Y  H  V  S  H  S  *
gcccgcctcggcctcccaaagtggtgggattacaggcatgagcccctgtgcctggcctgatggcaccagttttgtggatctcagtgtttcttttcatat
ccaagaactgggtcttcttgtctccctccatccaccaaaaaaaaaaaaaaaaaaatacttcaaggaagcatggacaaaaatatcctcagacaagttaga
acatgagacagtcacgttcttcagtgtggcgctctgtggatgtttgggaaagtaatttggaaaatgagtagagcagggctccccaaccccccagccgtg
gacctgtgggctgttaggaactgggcctcccagcaggaggtgagcagtgggtgagggagtgaagctttgtattgacagccgctccccatcgggcgcatc
accgcctgagctccgcctcctgtcagatcagcggcagcgttagattctcatgagtgtgaaccctgttgtgaactctgcacgtgcaagggatctaggctgcacactccttacgagaatctaatgcccgacgatctgtcactgtctcccatcacccccagatgggactgtctagatgcaggaaaacaagcccagggctcccactgattctacattatggtgagctgtataattatttcattatatatcacaatgtaataataaaaataaagtacataagaaatgtaatgtgcaaaaaaaaaaaaaaaaaaaaaaaaaaaaaaaaaa


Full-length nucleotide and deduced amino acid sequence of the BORIS B2 isoform (GenBank: DQ778124).  BORIS B2 isoform is expressed from promoter B, contains ten zinc fingers, N-terminus (N24), C-terminus (C97), and 3'UTR belonging to BORIS subfamily 4 (BORIS_sf4). Two pair of primers with corresponding Tagman probes are depicted due to the fact that BORIS B2 isoform was detected as a part of  BORIS (sf4) and as an isoform containing Exon b/ Exon 3 splice site (Fig. S4E, Table S2). 

ggcaccagacgcggtgcacgaggcagagcccacaagccaaagacggagtgggccgagcatTccggccacgccttccgcggagcaaaaggaaccttccactgtgatgtctgcatgttcacctcttctagaatgtcaagttttaatcgtcatatgaaaactcacaccagtgagaagcctcacctgtgtcacctctgcctg
             M  F  T  S  S  R  M  S  S  F  N  R  H  M  K  T  H  T  S  E  K  P  H  L  C  H  L  C  L   
aaaaccttccgtacggtcactctgctgcggaaccatgttaacacccacacaggaaccaggccctacaagtgtaacgactgcaacatggcatttgtcacc
 K  T  F  R  T  V  T  L  L  R  N  H  V  N  T  H  T  G  T  R  P  Y  K  C  N  D  C  N  M  A  F  V  T
agtggagaactcgtccgacacaggcgctataaacatactcatgagaaaccctttaaatgttccatgtgcaagtatgccagtgtggaggcaagtaaattg
 S  G  E  L  V  R  H  R  R  Y  K  H  T  H  E  K  P  F  K  C  S  M  C  K  Y  A  S  V  E  A  S  K  L  
aagcgccatgtccgatcccacactggggagcgcccctttcagtgttgccagtgcagctatgccagcagagatacctacaagctgaaacgccacatgaga
 K  R  H  V  R  S  H  T  G  E  R  P  F  Q  C  C  Q  C  S  Y  A  S  R  D  T  Y  K  L  K  R  H  M  R  
acgcactcaggtgagaagccttacgaatgccacatctgccacacccgcttcacccagagcgggaccatgaaaatacatattctgcagaaacacggcgaa
 T  H  S  G  E  K  P  Y  E  C  H  I  C  H  T  R  F  T  Q  S  G  T  M  K  I  H  I  L  Q  K  H  G  E  
aatgtccccaaataccagtgtccccattgtgccaccatcattgcacggaaaagcgacctacgtgtgcatatgcgcaacttgcatgcttacagcgctgca
 N  V  P  K  Y  Q  C  P  H  C  A  T  I  I  A  R  K  S  D  L  R  V  H  M  R  N  L  H  A  Y  S  A  A  
gagctgaaatgccgctactgttctgctgtcttccatgaacgctatgccctcattcagcaccagaaaactcataagaatgagaagaggttcaagtgcaaa
 E  L  K  C  R  Y  C  S  A  V  F  H  E  R  Y  A  L  I  Q  H  Q  K  T  H  K  N  E  K  R  F  K  C  K  
cactgcagttatgcctgcaagcaggaacgtcatatgaccgctcacattcgtacccacactggagagaaaccattcacctgcctttcttgcaataaatgt
 H  C  S  Y  A  C  K  Q  E  R  H  M  T  A  H  I  R  T  H  T  G  E  K  P  F  T  C  L  S  C  N  K  C  
ttccgacagaagcaacttctaaacgctcacttcaggaaataccacgatgcaaatttcatcccgactgtttacaaatgctccaagtgtggcaaaggcttt
 F  R  Q  K  Q  L  L  N  A  H  F  R  K  Y  H  D  A  N  F  I  P  T  V  Y  K  C  S  K  C  G  K  G  F  
tcccgctggattaacctgcacagacattcggagaagtgtggatcaggggaagcaaagtcggctgcttcaggaaagggaagaagaacaagaaagaggaag
 S  R  W  I  N  L  H  R  H  S  E  K  C  G  S  G  E  A  K  S  A  A  S  G  K  G  R  R  T  R  K  R  K  
cagaccatcctgaaggaagccacaaagggtcagaaggaagctgcgaagggatggaaggaagccgcgaacggagacggtgtgatctcagctcaccgcaac
 Q  T  I  L  K  E  A  T  K  G  Q  K  E  A  A  K  G  W  K  E  A  A  N  G  D  G  V  I  S  A  H  R  N  
ctctgcctcctgggttcaagtgattctcatgcctcagtctccggagctgggattacagatgcccgccaccacgcctggctaattgttctattattttta
 L  C  L  L  G  S  S  D  S  H  A  S  V  S  G  A  G  I  T  D  A  R  H  H  A  W  L  I  V  L  L  F  L  
gtagagatggggttttaccatgtctctcactcctgacctcaagtgatctgcccgcctcggcctcccaaagtggtgggattacaggcatgagcccctgtg
 V  E  M  G  F  Y  H  V  S  H  S  *
cctggcctgatggcaccagttttgtggatctcagtgtttcttttcatatccaagaactgggtcttcttgtctccctccatccaccaaaaaaaaaaaaaaaaaaatacttcaaggaagcatggacaaaaatatcctcagacaagttagaacatgagacagtcacgttcttcagtgtggcgctctgtggatgtttgggaaagtaatttggaaaatgagtagagcagggctccccaaccccccagccgtggacctgtgggctgttaggaactgggcctcccagcaggaggtgagcagtgggtgagggagtgaagctttgtattgacagccgctccccatcgggcgcatcaccgcctgagctccgcctcctgtcagatcagcggcagcgttagattctcatgagtgtgaaccctgttgtgaactctgcacgtgcaagggatctaggctgcacactccttacgagaatctaatgcccgacgatctgtcactgtctcccatcacccccagatgggactgtctagatgcaggaaaacaagcccagggctcccactgattctacattatggtgagctgtataattatttcattatatatcacaatgtaataataaaaataaagtacataagaaatgtaatgtgcaaaaaaaaaaaaaaaaaaaaaaaaaaaaaaaaaa


Full-length nucleotide and deduced amino acid sequence of the BORIS B3 isoform (GenBank: DQ778125).  BORIS B3 isoform is expressed from promoter B, contains eleven zinc fingers, N-terminus (N53), C-terminus (C97), and 3'UTR belonging to BORIS subfamily 4 (BORIS_sf4). Two pair of primers with corresponding Tagman probes are depicted due to the fact that BORIS B3 isoform was detected as a part of  BORIS (sf4) and as an isoform containing Exon b/ Exon 2 splice site (Fig. S4E, Table S2). 


ggcaccagacgcggtgcacgaggcagagcccacaagccaaagacggagtgggccgagcattccggccacgccttccgcgctcgaggaagagcaggagaagaaccagttattggctgaaagaAcaaaggagcagctcttttttgtggaaacaatgtcaggagatgaaagaagtgacgaaattgttctcacagtttcaaa
                                                     M  S  G  D  E  R  S  D  E  I  V  L  T  V  S  N  
ttcaaatgtggaagaacaagaggatcaacctacagctggtcaagcagatgctgaaaaggccaaatctacaaaaaatcaaagaaagacaaagggagcaaa
  S  N  V  E  E  Q  E  D  Q  P  T  A  G  Q  A  D  A  E  K  A  K  S  T  K  N  Q  R  K  T  K  G  A  K  
aggaaccttccactgtgatgtctgcatgttcacctcttctagaatgtcaagttttaatcgtcatatgaaaactcacaccagtgagaagcctcacctgtg
  G  T  F  H  C  D  V  C  M  F  T  S  S  R  M  S  S  F  N  R  H  M  K  T  H  T  S  E  K  P  H  L  C  
tcacctctgcctgaaaaccttccgtacggtcactctgctgcggaaccatgttaacacccacacaggaaccaggccctacaagtgtaacgactgcaacat
  H  L  C  L  K  T  F  R  T  V  T  L  L  R  N  H  V  N  T  H  T  G  T  R  P  Y  K  C  N  D  C  N  M  
ggcatttgtcaccagtggagaactcgtccgacacaggcgctataaacatactcatgagaaaccctttaaatgttccatgtgcaagtatgccagtgtgga
  A  F  V  T  S  G  E  L  V  R  H  R  R  Y  K  H  T  H  E  K  P  F  K  C  S  M  C  K  Y  A  S  V  E  
ggcaagtaaattgaagcgccatgtccgatcccacactggggagcgcccctttcagtgttgccagtgcagctatgccagcagagatacctacaagctgaa
  A  S  K  L  K  R  H  V  R  S  H  T  G  E  R  P  F  Q  C  C  Q  C  S  Y  A  S  R  D  T  Y  K  L  K  
acgccacatgagaacgcactcaggtgagaagccttacgaatgccacatctgccacacccgcttcacccagagcgggaccatgaaaatacatattctgca
  R  H  M  R  T  H  S  G  E  K  P  Y  E  C  H  I  C  H  T  R  F  T  Q  S  G  T  M  K  I  H  I  L  Q  
gaaacacggcgaaaatgtccccaaataccagtgtccccattgtgccaccatcattgcacggaaaagcgacctacgtgtgcatatgcgcaacttgcatgc
  K  H  G  E  N  V  P  K  Y  Q  C  P  H  C  A  T  I  I  A  R  K  S  D  L  R  V  H  M  R  N  L  H  A  
ttacagcgctgcagagctgaaatgccgctactgttctgctgtcttccatgaacgctatgccctcattcagcaccagaaaactcataagaatgagaagag
  Y  S  A  A  E  L  K  C  R  Y  C  S  A  V  F  H  E  R  Y  A  L  I  Q  H  Q  K  T  H  K  N  E  K  R  
gttcaagtgcaaacactgcagttatgcctgcaagcaggaacgtcatatgaccgctcacattcgtacccacactggagagaaaccattcacctgcctttc
  F  K  C  K  H  C  S  Y  A  C  K  Q  E  R  H  M  T  A  H  I  R  T  H  T  G  E  K  P  F  T  C  L  S  
ttgcaataaatgtttccgacagaagcaacttctaaacgctcacttcaggaaataccacgatgcaaatttcatcccgactgtttacaaatgctccaagtg
  C  N  K  C  F  R  Q  K  Q  L  L  N  A  H  F  R  K  Y  H  D  A  N  F  I  P  T  V  Y  K  C  S  K  C  
tggcaaaggcttttcccgctggattaacctgcacagacattcggagaagtgtggatcaggggaagcaaagtcggctgcttcaggaaagggaagaagaac
  G  K  G  F  S  R  W  I  N  L  H  R  H  S  E  K  C  G  S  G  E  A  K  S  A  A  S  G  K  G  R  R  T  
aagaaagaggaagcagaccatcctgaaggaagccacaaagggtcagaaggaagctgcgaagggatggaaggaagccgcgaacggagacggtgtgatctc
  R  K  R  K  Q  T  I  L  K  E  A  T  K  G  Q  K  E  A  A  K  G  W  K  E  A  A  N  G  D  G  V  I  S  
agctcaccgcaacctctgcctcctgggttcaagtgattctcatgcctcagtctccggagctgggattacagatgcccgccaccacgcctggctaattgt
  A  H  R  N  L  C  L  L  G  S  S  D  S  H  A  S  V  S  G  A  G  I  T  D  A  R  H  H  A  W  L  I  V  
tctattatttttagtagagatggggttttaccatgtctctcactcctgacctcaagtgatctgcccgcctcggcctcccaaagtggtgggattacaggc
  L  L  F  L  V  E  M  G  F  Y  H  V  S  H  S  *
atgagcccctgtgcctggcctgatggcaccagttttgtggatctcagtgtttcttttcatatccaagaactgggtcttcttgtctccctccatccacca
aaaaaaaaaaaaaaaaaatacttcaaggaagcatggacaaaaatatcctcagacaagttagaacatgagacagtcacgttcttcagtgtggcgctctgtggatgtttgggaaagtaatttggaaaatgagtagagcagggctccccaaccccccagccgtggacctgtgggctgttaggaactgggcctcccagcaggaggtgagcagtgggtgagggagtgaagctttgtattgacagccgctccccatcgggcgcatcaccgcctgagctccgcctcctgtcagatcagcggcagcgttagattctcatgagtgtgaaccctgttgtgaactctgcacgtgcaagggatctaggctgcacactccttacgagaatctaatgcccgacgatctgtcactgtctcccatcacccccagatgggactgtctagatgcaggaaaacaagcccagggctcccactgattctacattatggtgagctgtataattatttcattatatatcacaatgtaataataaaaataaagtacataagaaatgtaatgtgcaaaaaaaaaaaaaaaaaaaaaaaaaaaaaaaaaa


Full-length nucleotide and deduced amino acid sequence of the BORIS C4 isoform (GenBank: DQ778116).  BORIS C4 isoform is expressed from promoter C, contains five zinc fingers, N-terminus (N258), C-terminus (C35), and 3'UTR belonging to BORIS subfamily 4 (BORIS_sf4). Two pair of primers with corresponding Tagman probes are depicted due to the fact that BORIS C4 isoform was detected as a part of  BORIS (sf4) and as a single isoform (Fig. S4, Table S2). 

agggtaaagcaggggccctgccaggcctccgagggagtgtgcttggtctggccgagggctgcttggccaagtctgggtgggctcgaggccactaggcccaaagcctgcctggctctgagggtgctaggtctagaaccgtgcacgaggggaatgcctgctcgggcccgaacctcgctgggcgccgggtgtgcactggcccggggcctgcttggacctgaaacttgctaggcccaggatatgcactggccgagagcctgctgggcccaaaccttactaggcccaggatgttcactgactgaaccggctcaggcctaaccttgctaggcccaggatatgcactgggccagagtgtgctcaggcggaaccttgccaggcgcaggatgtgtgctggccctaagcctgctgaggcccaaacctgttcgttctagggttttgtacaaaatcctgctttagcctaaatcctgcttagccttgaccccctcctagacccaagccagatcagcattgttctgaccctactaagtccaaaaccttttgaggccagaccttgtttcaactccaaagcctgctaggttccagcaccccccgcatccctcctcataccacccccttctcccccctatggaaaccgcttgcttatttttcaaacaggccaagtcattatggcagccactgagatctctgtcctttctg
                                                                     M  A  A  T  E  I  S  V  L  S  
agcaattcaccaagatcaaagaactcgagttgatgccggaaaaaggcctgaaggaggaggaaaaagacggagtgtgcagagagaaagaccatcggagcc
E  Q  F  T  K  I  K  E  L  E  L  M  P  E  K  G  L  K  E  E  E  K  D  G  V  C  R  E  K  D  H  R  S 
ctagtgagttggaggccgagcgtacctctggggccttccaggacagcgtcctggaggaagaagtggagctggtgctggccccctcggaggagagcgaga
P  S  E  L  E  A  E  R  T  S  G  A  F  Q  D  S  V  L  E  E  E  V  E  L  V  L  A  P  S  E  E  S  E
agtacatcctgaccctgcagacggtgcacttcacttctgaagctgtggagttgcaggatatgagcttgctgagcatacagcagcaagaaggggtgcagg
K  Y  I  L  T  L  Q  T  V  H  F  T  S  E  A  V  E  L  Q  D  M  S  L  L  S  I  Q  Q  Q  E  G  V  Q  tggtggtgcaacagcctggccctgggttgctgtggcttgaggaagggccccggcagagcctgcagcagtgtgtggccattagtatccagcaagagctgt
V  V  V  Q  Q  P  G  P  G  L  L  W  L  E  E  G  P  R  Q  S  L  Q  Q  C  V  A  I  S  I  Q  Q  E  L 
actccccgcaagagatggaggtgttgcagttccacgctctagaggagaatgtgatggtggccagtgaagacagtaagttagcggtgagcctggctgaaa
Y  S  P  Q  E  M  E  V  L  Q  F  H  A  L  E  E  N  V  M  V  A  S  E  D  S  K  L  A  V  S  L  A  E 
ctactggactgatcaagctcgaggaagagcaggagaagaaccagttattggctgaaagaacaaaggagcagctcttttttgtggaaacaatgtcaggag
T  T  G  L  I  K  L  E  E  E  Q  E  K  N  Q  L  L  A  E  R  T  K  E  Q  L  F  F  V  E  T  M  S  G 
atgaaagaagtgacgaaattgttctcacagtttcaaattcaaatgtggaagaacaagaggatcaacctacagctggtcaagcagatgctgaaaaggcca
D  E  R  S  D  E  I  V  L  T  V  S  N  S  N  V  E  E  Q  E  D  Q  P  T  A  G  Q  A  D  A  E  K  A 
aatctacaaaaaatcaaagaaagacaaagggagcaaaaggaaccttccactgtgatgtctgcatgttcacctcttctagaatgtcaagttttaatcgtc
K  S  T  K  N  Q  R  K  T  K  G  A  K  G  T  F  H  C  D  V  C  M  F  T  S  S  R  M  S  S  F  N  R 
atatgaaaactcacaccagtgagaagcctcacctgtgtcacctctgcctgaaaaccttccgtacggtcactctgctgcggaaccatgttaacacccaca
H  M  K  T  H  T  S  E  K  P  H  L  C  H  L  C  L  K  T  F  R  T  V  T  L  L  R  N  H  V  N  T  H 
caggaaccaggccctacaagtgtaacgactgcaacatggcatttgtcaccagtggagaactcgtccgacacaggcgctataaacatactcatgagaaac
T  G  T  R  P  Y  K  C  N  D  C  N  M  A  F  V  T  S  G  E  L  V  R  H  R  R  Y  K  H  T  H  E  K  
cctttaaatgttccatgtgcaagtatgccagtgtggaggcaagtaaattgaagcgccatgtccgatcccacactggggagcgcccctttcagtgttgcc
P  F  K  C  S  M  C  K  Y  A  S  V  E  A  S  K  L  K  R  H  V  R  S  H  T  G  E  R  P  F  Q  C  C  
agtgcagctatgccagcagagatacctacaagctgaaacgccacatgagaacgcactcaGaagcaacttctaaacgctcacttcaggaaataccacgat 
Q  C  S  Y  A  S  R  D  T  Y  K  L  K  R  H  M  R  T  H  S  E  A  T  S  K  R  S  L  Q  E  I  P  R    
Gcaaatttcatcccgactgtttacaaatgctccaagtgtggcaaaggcttttcccgctggattaacctgcacagacattcggagaagtgtggatcaggg
C  K  F  H  P  D  C  L  Q  M  L  Q  V  W  Q  R  L  F  P  L  D  *
gaagcaaagtcggctgcttcaggaaagggaagaagaacaagaaagaggaagcagaccatcctgaaggaagccacaaagggtcagaaggaagctgcgaagggatggaaggaagccgcgaacggagacggtgtgatctcagctcaccgcaacctctgcctcctgggttcaagtgattctcatgcctcagtctccggagctgggattacagatgcccgccaccacgcctggctaattgttctattatttttagtagagatggggttttaccatgtctctcactcctgacctcaagtgatctgcccgcctcggcctcccaaagtggtgggattacaggcatgagcccctgtgcctggcctgatggcaccagttttgtggatctcagtgtttcttttcatatccaagaactgggtcttcttgtctccctccatccaccaaaaaaaaaaaaaaaaaaatacttcaaggaagcatggacaaaaatatcctcagacaagttagaacatgagacagtcacgttcttcagtgtggcgctctgtggatgtttgggaaagtaatttggaaaatgagtagagcagggctccccaaccccccagccgtggacctgtgggctgttaggaactgggcctcccagcaggaggtgagcagtgggtgagggagtgaagctttgtattgacagccgctccccatcgggcgcatcaccgcctgagctccgcctcctgtcagatcagcggcagcgttagattctcatgagtgtgaaccctgttgtgaactctgcacgtgcaagggatctaggctgcacactccttacgagaatctaatgcccgacgatctgtcactgtctcccatcacccccagatgggactgtctagatgcaggaaaacaagcccagggctcccactgattctacattatggtgagctgtataattatttcattatatatcacaatgtaataataaaaataaagtacataagaaatgtaatgtgcaaaaaaaaaaaaaaaaaaaaaaaaaaaaaaaaaa


Full-length nucleotide and deduced amino acid sequence of the BORIS C5 isoform (GenBank: DQ778117).  BORIS C5 isoform expresses from promoter C, contains one zinc fingers, N-terminus (N258), C-terminus (C53), and 3'UTR belonging to BORIS subfamily 4 (BORIS_sf4). Two pair of primers with corresponding Tagman probes are depicted due to the fact that BORIS C5 isoform was detected as a part of BORIS (sf4) and as a single isoform (Fig. S4E, Table S2). 


agggtaaagcaggggccctgccaggcctccgagggagtgtgcttggtctggccgagggctgcttggccaagtctgggtgggctcgaggccactaggcccaaagcctgcctggctctgagggtgctaggtctagaaccgtgcacgaggggaatgcctgctcgggcccgaacctcgctgggcgccgggtgtgcactggcccggggcctgcttggacctgaaacttgctaggcccaggatatgcactggccgagagcctgctgggcccaaaccttactaggcccaggatgttcactgactgaaccggctcaggcctaaccttgctaggcccaggatatgcactgggccagagtgtgctcaggcggaaccttgccaggcgcaggatgtgtgctggccctaagcctgctgaggcccaaacctgttcgttctagggttttgtacaaaatcctgctttagcctaaatcctgcttagccttgaccccctcctagacccaagccagatcagcattgttctgaccctactaagtccaaaaccttttgaggccagaccttgtttcaactccaaagcctgctaggttccagcaccccccgcatccctcctcataccacccccttctcccccctatggaaaccgcttgcttatttttcaaacaggccaagtcattatggcagccactgagatctctgtcctttctg
                                                                     M  A  A  T  E  I  S  V  L  S  
agcaattcaccaagatcaaagaactcgagttgatgccggaaaaaggcctgaaggaggaggaaaaagacggagtgtgcagagagaaagaccatcggagcc
E  Q  F  T  K  I  K  E  L  E  L  M  P  E  K  G  L  K  E  E  E  K  D  G  V  C  R  E  K  D  H  R  S 
ctagtgagttggaggccgagcgtacctctggggccttccaggacagcgtcctggaggaagaagtggagctggtgctggccccctcggaggagagcgaga
P  S  E  L  E  A  E  R  T  S  G  A  F  Q  D  S  V  L  E  E  E  V  E  L  V  L  A  P  S  E  E  S  E
agtacatcctgaccctgcagacggtgcacttcacttctgaagctgtggagttgcaggatatgagcttgctgagcatacagcagcaagaaggggtgcagg
K  Y  I  L  T  L  Q  T  V  H  F  T  S  E  A  V  E  L  Q  D  M  S  L  L  S  I  Q  Q  Q  E  G  V  Q  tggtggtgcaacagcctggccctgggttgctgtggcttgaggaagggccccggcagagcctgcagcagtgtgtggccattagtatccagcaagagctgt
V  V  V  Q  Q  P  G  P  G  L  L  W  L  E  E  G  P  R  Q  S  L  Q  Q  C  V  A  I  S  I  Q  Q  E  L 
actccccgcaagagatggaggtgttgcagttccacgctctagaggagaatgtgatggtggccagtgaagacagtaagttagcggtgagcctggctgaaa
Y  S  P  Q  E  M  E  V  L  Q  F  H  A  L  E  E  N  V  M  V  A  S  E  D  S  K  L  A  V  S  L  A  E 
ctactggactgatcaagctcgaggaagagcaggagaagaaccagttattggctgaaagaacaaaggagcagctcttttttgtggaaacaatgtcaggag
T  T  G  L  I  K  L  E  E  E  Q  E  K  N  Q  L  L  A  E  R  T  K  E  Q  L  F  F  V  E  T  M  S  G 
atgaaagaagtgacgaaattgttctcacagtttcaaattcaaatgtggaagaacaagaggatcaacctacagctggtcaagcagatgctgaaaaggcca
D  E  R  S  D  E  I  V  L  T  V  S  N  S  N  V  E  E  Q  E  D  Q  P  T  A  G  Q  A  D  A  E  K  A 
aatctacaaaaaatcaaagaaagacaaagggagcaaaaggaaccttccactgtgatgtctgcatgttcacctcttctagaatgtcaagttttaatcgtc
K  S  T  K  N  Q  R  K  T  K  G  A  K  G  T  F  H  C  D  V  C  M  F  T  S  S  R  M  S  S  F  N  R 
atatgaaaactcacaccagtgagaagcctcacctgtgtcacctctgcctcctgggttcaagtgattctcatgcctcagtctccggagctgggattacag
H  M  K  T  H  T  S  E  K  P  H  L  C  H  L  C  L  L  G  S  S  D  S  H  A  S  V  S  G  A  G  I  T  
Atgcccgccaccacgcctggctaattgttctattatttttagtagagatggggttttaccatgtctctcactcctgacctcaagtgatctgcccgcctc
D  A  R  H  H  A  W  L  I  V  L  L  F  L  V  E  M  G  F  Y  H  V  S  H  S  *
Ggcctcccaaagtggtgggattacaggcatgagcccctgtgcctggcctgatggcaccagttttgtggatctcagtgtttcttttcatatccaagaactgggtcttcttgtctccctccatccaccaaaaaaaaaaaaaaaaaaatacttcaaggaagcatggacaaaaatatcctcagacaagttagaacatgagacagtcacgttcttcagtgtggcgctctgtggatgtttgggaaagtaatttggaaaatgagtagagcagggctccccaaccccccagccgtggacctgtgggctgttaggaactgggcctcccagcaggaggtgagcagtgggtgagggagtgaagctttgtattgacagccgctccccatcgggcgcatcaccgcctgagctccgcctcctgtcagatcagcggcagcgttagattctcatgagtgtgaaccctgttgtgaactctgcacgtgcaagggatctaggctgcacactccttacgagaatctaatgcccgacgatctgtcactgtctcccatcacccccagatgggactgtctagatgcaggaaaacaagcccagggctcccactgattctacattatggtgagctgtataattatttcattatatatcacaatgtaataataaaaataaagtacataagaaatgtaatgtgcaaaaaaaaaaaaaaaaaaaaaaaaaaaaaaaaaa


  


Full-length nucleotide and deduced amino acid sequence of the BORIS C8 isoform (GenBank: DQ778118).  BORIS C8 isoform is expressed from promoter C, contains six ZFs, N-terminus (N258), C-terminus (C30), and 3'UTR belonging to BORIS subfamily 4 (BORIS_sf4). Two pair of primers with corresponding Tagman probes are depicted due to the fact that BORIS C8 isoform was detected as a part of BORIS (sf4) and as a single isoform (Fig. S4E, Table S2). 
agggtaaagcaggggccctgccaggcctccgagggagtgtgcttggtctggccgagggctgcttggccaagtctgggtgggctcgaggccactaggcccaaagcctgcctggctctgagggtgctaggtctagaaccgtgcacgaggggaatgcctgctcgggcccgaacctcgctgggcgccgggtgtgcactggcccggggcctgcttggacctgaaacttgctaggcccaggatatgcactggccgagagcctgctgggcccaaaccttactaggcccaggatgttcactgactgaaccggctcaggcctaaccttgctaggcccaggatatgcactgggccagagtgtgctcaggcggaaccttgccaggcgcaggatgtgtgctggccctaagcctgctgaggcccaaacctgttcgttctagggttttgtacaaaatcctgctttagcctaaatcctgcttagccttgaccccctcctagacccaagccagatcagcattgttctgaccctactaagtccaaaaccttttgaggccagaccttgtttcaactccaaagcctgctaggttccagcaccccccgcatccctcctcataccacccccttctcccccctatggaaaccgcttgcttatttttcaaacaggccaagtcattatggcagccactgagatctctgtcctttctg
                                                                     M  A  A  T  E  I  S  V  L  S  
agcaattcaccaagatcaaagaactcgagttgatgccggaaaaaggcctgaaggaggaggaaaaagacggagtgtgcagagagaaagaccatcggagcc
E  Q  F  T  K  I  K  E  L  E  L  M  P  E  K  G  L  K  E  E  E  K  D  G  V  C  R  E  K  D  H  R  S 
ctagtgagttggaggccgagcgtacctctggggccttccaggacagcgtcctggaggaagaagtggagctggtgctggccccctcggaggagagcgaga
P  S  E  L  E  A  E  R  T  S  G  A  F  Q  D  S  V  L  E  E  E  V  E  L  V  L  A  P  S  E  E  S  E
agtacatcctgaccctgcagacggtgcacttcacttctgaagctgtggagttgcaggatatgagcttgctgagcatacagcagcaagaaggggtgcagg
K  Y  I  L  T  L  Q  T  V  H  F  T  S  E  A  V  E  L  Q  D  M  S  L  L  S  I  Q  Q  Q  E  G  V  Q  tggtggtgcaacagcctggccctgggttgctgtggcttgaggaagggccccggcagagcctgcagcagtgtgtggccattagtatccagcaagagctgt
V  V  V  Q  Q  P  G  P  G  L  L  W  L  E  E  G  P  R  Q  S  L  Q  Q  C  V  A  I  S  I  Q  Q  E  L 
actccccgcaagagatggaggtgttgcagttccacgctctagaggagaatgtgatggtggccagtgaagacagtaagttagcggtgagcctggctgaaa
Y  S  P  Q  E  M  E  V  L  Q  F  H  A  L  E  E  N  V  M  V  A  S  E  D  S  K  L  A  V  S  L  A  E 
ctgctggactgatcaagctcgaggaagagcaggagaagaaccagttattggctgaaagaacaaaggagcagctcttttttgtggaaacaatgtcaggag
T  A  G  L  I  K  L  E  E  E  Q  E  K  N  Q  L  L  A  E  R  T  K  E  Q  L  F  F  V  E  T  M  S  G 
atgaaagaagtgacgaaattgttctcacagtttcaaattcaaatgtggaagaacaagaggatcaacctacagctggtcaagcagatgctgaaaaggcca
D  E  R  S  D  E  I  V  L  T  V  S  N  S  N  V  E  E  Q  E  D  Q  P  T  A  G  Q  A  D  A  E  K  A 
aatctacaaaaaatcaaagaaagacaaagggagcaaaaggaaccttccactgtgatgtctgcatgttcacctcttctagaatgtcaagttttaatcgtc
K  S  T  K  N  Q  R  K  T  K  G  A  K  G  T  F  H  C  D  V  C  M  F  T  S  S  R  M  S  S  F  N  R 
atatgaaaactcacaccagtgagaagcctcacctgtgtcacctctgcctgaaaaccttccgtacggtcactctgctgcggaaccatgttaacacccaca
H  M  K  T  H  T  S  E  K  P  H  L  C  H  L  C  L  K  T  F  R  T  V  T  L  L  R  N  H  V  N  T  H 
caggaaccaggccctacaagtgtaacgactgcaacatggcatttgtcaccagtggagaactcgtccgacacaggcgctataaacatactcatgagaaac
T  G  T  R  P  Y  K  C  N  D  C  N  M  A  F  V  T  S  G  E  L  V  R  H  R  R  Y  K  H  T  H  E  K  
cctttaaatgttccatgtgcaagtatgccagtgtggaggcaagtaaattgaagcgccatgtccgatcccacactggggagcgcccctttcagtgttgcc
P  F  K  C  S  M  C  K  Y  A  S  V  E  A  S  K  L  K  R  H  V  R  S  H  T  G  E  R  P  F  Q  C  C  
agtgcagctatgccagcagagatacctacaagctgaaacgccacatgagaacgcactcaggtgagaagccttacgaatgccacatctgccacacccgct
Q  C  S  Y  A  S  R  D  T  Y  K  L  K  R  H  M  R  T  H  S  G  E  K  P  Y  E  C  H  I  C  H  T  R  
tcacccagagcgggaccatgaaaatacatattctgcagaaacacggcgaaaatgtccccaaataccagtgtccccattgtgccaccatcattgcacgga
F  T  Q  S  G  T  M  K  I  H  I  L  Q  K  H  G  E  N  V  P  K  Y  Q  C  P  H  C  A  T  I  I  A  R    
aaagcgacctacgattcctgggcctcccttttccatgatcttatttctctttccaaaatatatgacactgtgatacacaaaaatatgttagcagaacaa
K  S  D  L  R  F  L  G  L  P  F  P  *
aaaatttgacccttttgcccaaagagattctggagatgagatgaatttttttgaaaacctttctgaaaggcgaaaggtgttccacaaagtcttcacttttatttttcatctggaccattctgtcattgttgccgtagataccagcacatcacaaaacacagtccagtggggcttggtggggcacaagtctagccagttgctagacttttttgacaccatggcaaagtaacatggtttatatccatctcatgccagtgggtgatacactccacatagccctataagatgctttataaacacattaattcttccttcccatgactgtatgagaaatcaagctctctaaatgtctgagtcactgtgggtaaggtggagtagctccatcacacagctgtccattgtcatttggcttcttacacaagtatcacaagaccatcatgctttagacttagatgagcaaacccagccagaccttaccatgtgtctcaggttaatttcaacataggaaataacagggcactgacttctaacaacgagtggattatttaaggtgtgcatatgcgcaacttgcatgcttacagcgctgcagagctgaaatgccgctactgttctgctgtcttccatgaacgctatgccctcattcagcaccagaaaactcataagaatgagaagaggttcaagtgcaaacactgcagttatgcctgcaagcaggaacgtcatatgaccgctcacattcgtacccacactggagagaaaccattcacctgcctttcttgcaataaatgtttccgacagaagcaacttctaaacgctcacttcaggaaataccacgatgcaaatttcatcccgactgtttacaaatgctccaagtgtggcaaaggcttttcccgctggattaacctgcacagacattcggagaagtgtggatcaggggaagcaaagtcggctgcttcaggaaagggaagaagaacaagaaagaggaagcagaccatcctgaaggaagccacaaagggtcagaaggaagctgcgaagggatggaaggaagccgcgaacggagacggtgtgatctcagctcaccgcaacctctgcctcctgggttcaagtgattctcatgcctcagtctccggagctgggattacagatgcccgccaccacgcctggctaattgttctattatttttagtagagatggggttttaccatgtctctcactcctgacctcaagtgatctgcccgcctcggcctcccaaagtggtgggattacaggcatgagcccctgtgcctggcctgatggcaccagttttgtggatctcagtgtttcttttcatatccaagaactgggtcttcttgtctccctccatccaccaaaaaaaaaaaaaaaaaaatacttcaaggaagcatggacaaaaatatcctcagacaagttagaacatgagacagtcacgttcttcagtgtggcgctctgtggatgtttgggaaagtaatttggaaaatgagtagagcagggctccccaaccccccagccgtggacctgtgggctgttaggaactgggcctcccagcaggaggtgagcagtgggtgagggagtgaagctttgtattgacagccgctccccatcgggcgcatcaccgcctgagctccgcctcctgtcagatcagcggcagcgttagattctcatgagtgtgaaccctgttgtgaactctgcacgtgcaagggatctaggctgcacactccttacgagaatctaatgcccgacgatctgtcactgtctcccatcacccccagatgggactgtctagatgcaggaaaacaagcccagggctcccactgattctacattatggtgagctgtataattatttcattatatatcacaatgtaataataaaaataaagtacataagaaatgtaatgtgcaaaaaaaaaaaaaaaaaaaaaaaaaaaaaaaaaa


Full-length nucleotide and deduced amino acid sequence of the BORIS A5 isoform (GenBank: DQ778122). BORIS A5 isoform is expressed from promoter A, contains ten zinc fingers, N-terminus (N258), C-terminus (C90), and 3'UTR belonging to BORIS subfamily 3 (BORIS_sf3). 
     
attccacccctccccccagtatctcagtgcctcctgtgggccctcctcccctccttatccattccccctcagatctcccaggccccctgcaggccctcgtgccctccttacttcccccccgggtctcccagcgccccctgcggggccctcctcccttcctcatccacttcaaccccaaggccaagtcattatggcagc
                                                                                            M  A  A  
cactgagatctctgtcctttctgagcaattcaccaagatcaaagaactcgagttgatgccggaaaaaggcctgaaggaggaggaaaaagacggagtgtg
  T  E  I  S  V  L  S  E  Q  F  T  K  I  K  E  L  E  L  M  P  E  K  G  L  K  E  E  E  K  D  G  V  C  
cagagagaaagaccatcggagccctagtgagttggaggccgagcgtacctctggggccttccaggacagcgtcctggaggaagaagtggagctggtgct
  R  E  K  D  H  R  S  P  S  E  L  E  A  E  R  T  S  G  A  F  Q  D  S  V  L  E  E  E  V  E  L  V  L  
ggccccctcggaggagagcgagaagtacatcctgaccctgcagacggtgcacttcacttctgaagctgtggagttgcaggatatgagcttgctgagcat
  A  P  S  E  E  S  E  K  Y  I  L  T  L  Q  T  V  H  F  T  S  E  A  V  E  L  Q  D  M  S  L  L  S  I  
acagcagcaagaaggggtgcaggtggtggtgcaacagcctggccctgggttgctgtggcttgaggaagggccccggcagagcctgcagcagtgtgtggc
  Q  Q  Q  E  G  V  Q  V  V  V  Q  Q  P  G  P  G  L  L  W  L  E  E  G  P  R  Q  S  L  Q  Q  C  V  A  
cattagtatccagcaagagctgtactccccgcaagagatggaggtgttgcagttccacgctctagaggagaatgtgatggtggccagtgaagacagtaa
  I  S  I  Q  Q  E  L  Y  S  P  Q  E  M  E  V  L  Q  F  H  A  L  E  E  N  V  M  V  A  S  E  D  S  K  
gttagcggtgagcctggctgaaactgctggactgatcaagctcgaggaagagcaggagaagaaccagttattggctgaaagaacaaaggagcagctctt
  L  A  V  S  L  A  E  T  A  G  L  I  K  L  E  E  E  Q  E  K  N  Q  L  L  A  E  R  T  K  E  Q  L  F  
ttttgtggaaacaatgtcaggagatgaaagaagtgacgaaattgttctcacagtttcaaattcaaatgtggaagaacaagaggatcaacctacagctgg
  F  V  E  T  M  S  G  D  E  R  S  D  E  I  V  L  T  V  S  N  S  N  V  E  E  Q  E  D  Q  P  T  A  G  
tcaagcagatgctgaaaaggccaaatctacaaaaaatcaaagaaagacaaagggagcaaaaggaaccttccactgtgatgtctgcatgttcacctcttc
  Q  A  D  A  E  K  A  K  S  T  K  N  Q  R  K  T  K  G  A  K  G  T  F  H  C  D  V  C  M  F  T  S  S  
tagaatgtcaagttttaatcgtcatatgaaaactcacaccagtgagaagcctcacctgtgtcacctctgcctgaaaaccttccgtacggtcactctgct
  R  M  S  S  F  N  R  H  M  K  T  H  T  S  E  K  P  H  L  C  H  L  C  L  K  T  F  R  T  V  T  L  L  
gcggaaccatgttaacacccacacaggaaccaggccctacaagtgtaacgactgcaacatggcatttgtcaccagtggagaactcgtccgacacaggcg
  R  N  H  V  N  T  H  T  G  T  R  P  Y  K  C  N  D  C  N  M  A  F  V  T  S  G  E  L  V  R  H  R  R  
ctataaacatactcatgagaaaccctttaaatgttccatgtgcaagtatgccagtgtggaggcaagtaaattgaagcgccatgtccgatcccacactgg
  Y  K  H  T  H  E  K  P  F  K  C  S  M  C  K  Y  A  S  V  E  A  S  K  L  K  R  H  V  R  S  H  T  G  
ggagcgcccctttcagtgttgccagtgcagctatgccagcagagatacctacaagctgaaacgccacatgagaacgcactcaggtgagaagccttacga
  E  R  P  F  Q  C  C  Q  C  S  Y  A  S  R  D  T  Y  K  L  K  R  H  M  R  T  H  S  G  E  K  P  Y  E  
atgccacatctgccacacccgcttcacccagagcgggaccatgaaaatacatattctgcagaaacacggcgaaaatgtccccaaataccagtgtcccca
  C  H  I  C  H  T  R  F  T  Q  S  G  T  M  K  I  H  I  L  Q  K  H  G  E  N  V  P  K  Y  Q  C  P  H  
ttgtgccaccatcattgcacggaaaagcgacctacgtgtgcatatgcgcaacttgcatgcttacagcgctgcagagctgaaatgccgctactgttctgc
  C  A  T  I  I  A  R  K  S  D  L  R  V  H  M  R  N  L  H  A  Y  S  A  A  E  L  K  C  R  Y  C  S  A  
tgtcttccatgaacgctatgccctcattcagcaccagaaaactcataagaatgagaagaggttcaagtgcaaacactgcagttatgcctgcaagcagga
  V  F  H  E  R  Y  A  L  I  Q  H  Q  K  T  H  K  N  E  K  R  F  K  C  K  H  C  S  Y  A  C  K  Q  E  
acgtcatatgaccgctcacattcgtacccacactggagagaaaccattcacctgcctttcttgcaataaatgtttccgacagaagcaacttctaaacgc
  R  H  M  T  A  H  I  R  T  H  T  G  E  K  P  F  T  C  L  S  C  N  K  C  F  R  Q  K  Q  L  L  N  A  
tcacttcaggaaataccacgatgcaaatttcatcccgactgtttacaaatgctccaagtgtggcaaaggcttttcccgctggattctctgggttgggaa
  H  F  R  K  Y  H  D  A  N  F  I  P  T  V  Y  K  C  S  K  C  G  K  G  F  S  R  W  I  L  W  V  G  N  
ctcggaagtggctgaactgggtggtcctggctcagggccactcctgaggctgcagtcaggatgtccgccagggctgcatcatccgaaggctggactggg
  S  E  V  A  E  L  G  G  P  G  S  G  P  L  L  R  L  Q  S  G  C  P  P  G  L  H  H  P  K  A  G  L  G  
gccagaggatccacttccaggacagctccgccacacaactgctggcaccggcctcagttccttgctacagggacctctctgcagggctgcttgagtgtc
  P  E  D  P  L  P  G  Q  L  R  H  T  T  A  G  T  G  L  S  S  L  L  Q  G  P  L  C  R  A  A  *
ctcctgactcagagcaagtgagagagtgcaagtaggaagccatggtgccttttgcagtctagtcctaaaagcggcacaacagccagctgtgggggctcacacctgtaatcccagtacttcgggaggccaaggcaggtggataacttgaggccaggagctcaagaccagcctggccaacatggtgaaaccctgtctctactttaaaaaaaaaaaaagaaaatagctaggcgtggtagtgcacgcttgtaatcccagctacttgggtggttgggacgggaggatcacttgaacccaggagacagaggttgcagtgagctgagattgtgccactggcctccagcctgggcgacagagcaagactctgtctcaaaaaaaaaaaaaaagtggcacattctagtcacaagaaatgaaccatgaagtcccacccacactcgagtggggaatgaagtttcttttgaaaggaacagcatcaaagaatttcaggacatttaaaaaccactgcacatggctagctagatccttggaccatgtaggcttcagcctaactttataaaagttttataaaagttgaatttcctgaagttgactatttttttaaaaatatttcttcttaaaacagtagtgtagttgtgtttttctgagtagcacagtgggatagttctggaatgtcaaccctattagtttaaaatttcaaataaaagctgtggcagtgctcccaaaacaccattgctcatgcaaaaattgtgaagagttaaaattaacgagtagaattctaaagagaaaaaaactgctctttggtgcaagaaataaaaatgtagagaaaagaacaagtctgcaaaaaaaaaaaaaaaaaaaaaaaaaa 


 Full-length nucleotide and deduced amino acid sequence of the BORIS A6 isoform (GenBank: DQ778123). BORIS A6 isoform is expressed from promoter A, contains ten zinc fingers, N-terminus (N258), C-terminus (C36), and 3'UTR belonging to BORIS subfamily 3 (BORIS_sf3). Two pair of primers with corresponding Tagman probes are depicted due to the fact that BORIS A6 isoform was detected as a part of  BORIS (sf3) and as a single isoform (Fig. S4D, Table S2). 
 
attccacccctccccccagtatctcagtgcctcctgtgggccctcctcccctccttatccattccccctcagatctcccaggccccctgcaggccctcgtgccctccttacttcccccccgggtctcccagcgccccctgcggggccctcctcccttcctcatccacttcaaccccaaggccaagtcattatggcagc
                                                                                            M  A  A  
cactgagatctctgtcctttctgagcaattcaccaagatcaaagaactcgagttgatgccggaaaaaggcctgaaggaggaggaaaaagacggagtgtg
  T  E  I  S  V  L  S  E  Q  F  T  K  I  K  E  L  E  L  M  P  E  K  G  L  K  E  E  E  K  D  G  V  C  
cagagagaaagaccatcggagccctagtgagttggaggccgagcgtacctctggggccttccaggacagcgtcctggaggaagaagtggagctggtgct
  R  E  K  D  H  R  S  P  S  E  L  E  A  E  R  T  S  G  A  F  Q  D  S  V  L  E  E  E  V  E  L  V  L  
ggccccctcggaggagagcgagaagtacatcctgaccctgcagacggtgcacttcacttctgaagctgtggagttgcaggatatgagcttgctgagcat
  A  P  S  E  E  S  E  K  Y  I  L  T  L  Q  T  V  H  F  T  S  E  A  V  E  L  Q  D  M  S  L  L  S  I  
acagcagcaagaaggggtgcaggtggtggtgcaacagcctggccctgggttgctgtggcttgaggaagggccccggcagagcctgcagcagtgtgtggc
  Q  Q  Q  E  G  V  Q  V  V  V  Q  Q  P  G  P  G  L  L  W  L  E  E  G  P  R  Q  S  L  Q  Q  C  V  A  
cattagtatccagcaagagctgtactccccgcaagagatggaggtgttgcagttccacgctctagaggagaatgtgatggtggccagtgaagacagtaa
  I  S  I  Q  Q  E  L  Y  S  P  Q  E  M  E  V  L  Q  F  H  A  L  E  E  N  V  M  V  A  S  E  D  S  K  
gttagcggtgagcctggctgaaactgctggactgatcaagctcgaggaagagcaggagaagaaccagttattggctgaaagaacaaaggagcagctctt
  L  A  V  S  L  A  E  T  A  G  L  I  K  L  E  E  E  Q  E  K  N  Q  L  L  A  E  R  T  K  E  Q  L  F  
ttttgtggaaacaatgtcaggagatgaaagaagtgacgaaattgttctcacagtttcaaattcaaatgtggaagaacaagaggatcaacctacagctgg
  F  V  E  T  M  S  G  D  E  R  S  D  E  I  V  L  T  V  S  N  S  N  V  E  E  Q  E  D  Q  P  T  A  G  
tcaagcagatgctgaaaaggccaaatctacaaaaaatcaaagaaagacaaagggagcaaaaggaaccttccactgtgatgtctgcatgttcacctcttc
  Q  A  D  A  E  K  A  K  S  T  K  N  Q  R  K  T  K  G  A  K  G  T  F  H  C  D  V  C  M  F  T  S  S  
tagaatgtcaagttttaatcgtcatatgaaaactcacaccagtgagaagcctcacctgtgtcacctctgcctgaaaaccttccgtacggtcactctgct
  R  M  S  S  F  N  R  H  M  K  T  H  T  S  E  K  P  H  L  C  H  L  C  L  K  T  F  R  T  V  T  L  L  
gcggaaccatgttaacacccacacaggaaccaggccctacaagtgtaacgactgcaacatggcatttgtcaccagtggagaactcgtccgacacaggcg
  R  N  H  V  N  T  H  T  G  T  R  P  Y  K  C  N  D  C  N  M  A  F  V  T  S  G  E  L  V  R  H  R  R  
ctataaacatactcatgagaaaccctttaaatgttccatgtgcaagtatgccagtgtggaggcaagtaaattgaagcgccatgtccgatcccacactgg
  Y  K  H  T  H  E  K  P  F  K  C  S  M  C  K  Y  A  S  V  E  A  S  K  L  K  R  H  V  R  S  H  T  G  
ggagcgcccctttcagtgttgccagtgcagctatgccagcagagatacctacaagctgaaacgccacatgagaacgcactcaggtgagaagccttacga
E  R  P  F  Q  C  C  Q  C  S  Y  A  S  R  D  T  Y  K  L  K  R  H  M  R  T  H  S  G  E  K  P  Y  E  
atgccacatctgccacacccgcttcacccagagcgggaccatgaaaatacatattctgcagaaacacggcgaaaatgtccccaaataccagtgtcccca
  C  H  I  C  H  T  R  F  T  Q  S  G  T  M  K  I  H  I  L  Q  K  H  G  E  N  V  P  K  Y  Q  C  P  H  
ttgtgccaccatcattgcacggaaaagcgacctacgtgtgcatatgcgcaacttgcatgcttacagcgctgcagagctgaaatgccgctactgttctgc
  C  A  T  I  I  A  R  K  S  D  L  R  V  H  M  R  N  L  H  A  Y  S  A  A  E  L  K  C  R  Y  C  S  A  
tgtcttccatgaacgctatgccctcattcagcaccagaaaactcataagaatgagaagaggttcaagtgcaaacactgcagttatgcctgcaagcagga
  V  F  H  E  R  Y  A  L  I  Q  H  Q  K  T  H  K  N  E  K  R  F  K  C  K  H  C  S  Y  A  C  K  Q  E  
acgtcatatgaccgctcacattcgtacccacactggagagaaaccattcacctgcctttcttgcaataaatgtttccgacagaagcaacttctaaacgc
  R  H  M  T  A  H  I  R  T  H  T  G  E  K  P  F  T  C  L  S  C  N  K  C  F  R  Q  K  Q  L  L  N  A  
tcacttcaggaaataccacgatgcaaatttcatcccgactgtttacaaatgctccaagtgtggcaaaggcttttcccgctggattACCTCAAAATGGAG
H  F  R  K  Y  H  D  A  N  F  I  P  T  V  Y  K  C  S  K  C  G  K  G  F  S  R  W  I  T  S  K  W  S  TGGCTTAAAACCACAAACATTTATCACCTGACAGATTCTCTGGGTTGGGAACTCGGAAGTGGCTGAACTGGGTGGTCCTGGCTCAGGGCCACTCCTGAG
  G  L  K  P  Q  T  F  I  T  *  
GCTGCAGTCAGGATGTCCGCCAGGGCTGCATCATCCGAAGGCTGGACTGGGGCCAGAGGATCCACTTCCAGGACAGCTCCGCCACACAACTGCTGGCACCGGCCTCAGTTCCTTGCTACAGGGACCTCTCTGCAGGGCTGCTTGAGTGTCCTCCTGACTCAGAGCAAGTGAGAGAGTGCAAGTAGGAAGCCATGGTGCCTTTTGCAGTCTAGTCCTAAAAGCGGCACAACAGCCAGCTGTGGGGGCTCACACCTGTAATCCCAGTACTTCGGGAGGCCAAGGCAGGTGGATAACTTGAGGCCAGGAGCTCAAGACCAGCCTGGCCAACATGGTGAAACCctgtctctactttaaaaaaaaaaaaagaaaatagctaggcgtggtagtgcacgcttgtaatcccagctacttgggtggttgggacgggaggatcacttgaacccaggagacagaggttgcagtgagctgagattgtgccactggcctccagcctgggcgacagagcaagactctgtctcaaaaaaaaaaaaaaagtggcacattctagtcacaagaaatgaaccatgaagtcccacccacactcgagtggggaatgaagtttcttttgaaaggaacagcatcaaagaatttcaggacatttaaaaaccactgcacatggctagctagatccttggaccatgtaggcttcagcctaactttataaaagttttataaaagttgaatttcctgaagttgactatttttttaaaaatatttcttcttaaaacagtagtgtagttgtgtttttctgagtagcacagtgggatagttctggaatgtcaaccctattagtttaaaatttcaaataaaagctgtggcagtgctcccaaaacaccattgctcatgcaaaaattgtgaagagttaaaattaacgagtagaattctaaagagaaaaaaactgctctttggtgcaagaaataaaaatgtagagaaaagaacaagtctgcaaAAAAAAAAAAAAAAAAAAAAAAAAAAAAAAAAaA


  

Full-length nucleotide and deduced amino acid sequence of the BORIS B4 isoform (GenBank: DQ778126). BORIS B4 isoform expresses from promoter B, contains ten zinc fingers, N-terminus (N53), C-terminus (C97), and 3'UTR belonging to BORIS subfamily 3 (BORIS_sf3). Two pair of primers with corresponding Tagman probes are depicted due to the fact that BORIS B4 isoform was detected as a part of  BORIS (sf3) and as an isoform containing Exon b/ Exon 2 splice site (Fig. S4D, Table S2). 
 
ggcaccagacgcggtgcacgaggcagagccacaagccaaagacggagtgggccgagcattccggccacgccttccgcgctcgaggaagagcaggagaag
aaccagttattggctgaaagaacaaaggagcagctcttttttgtggaaacaatgtcaggagatgaaagaagtgacgaaattgttctcacagtttcaaat
                                                    M  S  G  D  E  R  S  D  E  I  V  L  T  V  S  N  
tcaaatgtggaagaacaagaggatcaacctacagctggtcaagcagatgctgaaaaggccaaatctacaaaaaatcaaagaaagacaaagggagcaaaa
 S  N  V  E  E  Q  E  D  Q  P  T  A  G  Q  A  D  A  E  K  A  K  S  T  K  N  Q  R  K  T  K  G  A  K  
ggaaccttccactgtgatgtctgcatgttcacctcttctagaatgtcaagttttaatcgtcatatgaaaactcacaccagtgagaagcctcacctgtgt
 G  T  F  H  C  D  V  C  M  F  T  S  S  R  M  S  S  F  N  R  H  M  K  T  H  T  S  E  K  P  H  L  C  
cacctctgcctgaaaaccttccgtacggtcactctgctgcggaaccatgttaacacccacacaggaaccaggccctacaagtgtaacgactgcaacatg
 H  L  C  L  K  T  F  R  T  V  T  L  L  R  N  H  V  N  T  H  T  G  T  R  P  Y  K  C  N  D  C  N  M  
gcatttgtcaccagtggagaactcgtccgacacaggcgctataaacatactcatgagaaaccctttaaatgttccatgtgcaagtatgccagtgtggag
 A  F  V  T  S  G  E  L  V  R  H  R  R  Y  K  H  T  H  E  K  P  F  K  C  S  M  C  K  Y  A  S  V  E  
gcaagtaaattgaagcgccatgtccgatcccacactggggagcgcccctttcagtgttgccagtgcagctatgccagcagagatacctacaagctgaaa
 A  S  K  L  K  R  H  V  R  S  H  T  G  E  R  P  F  Q  C  C  Q  C  S  Y  A  S  R  D  T  Y  K  L  K  
cgccacatgagaacgcactcaggtgagaagccttacgaatgccacatctgccacacccgcttcacccagagcgggaccatgaaaatacatattctgcag
 R  H  M  R  T  H  S  G  E  K  P  Y  E  C  H  I  C  H  T  R  F  T  Q  S  G  T  M  K  I  H  I  L  Q  
aaacacggcgaaaatgtccccaaataccagtgtccccattgtgccaccatcattgcacggaaaagcgacctacgtgtgcatatgcgcaacttgcatgct
 K  H  G  E  N  V  P  K  Y  Q  C  P  H  C  A  T  I  I  A  R  K  S  D  L  R  V  H  M  R  N  L  H  A  
tacagcgctgcagagctgaaatgccgctactgttctgctgtcttccatgaacgctatgccctcattcagcaccagaaaactcataagaatgagaagagg
 Y  S  A  A  E  L  K  C  R  Y  C  S  A  V  F  H  E  R  Y  A  L  I  Q  H  Q  K  T  H  K  N  E  K  R  
ttcaagtgcaaacactgcagttatgcctgcaagcaggaacgtcatatgaccgctcacattcgtacccacactggagagaaaccattcacctgcctttct
 F  K  C  K  H  C  S  Y  A  C  K  Q  E  R  H  M  T  A  H  I  R  T  H  T  G  E  K  P  F  T  C  L  S  
tgcaataaatgtttccgacagaagcaacttctaaacgctcacttcaggaaataccacgatgcaaatttcatcccgactgtttacaaatgctccaagtgt
 C  N  K  C  F  R  Q  K  Q  L  L  N  A  H  F  R  K  Y  H  D  A  N  F  I  P  T  V  Y  K  C  S  K  C  
ggcaaaggcttttcccgctggattctctgggttgggaactcggaagtggctgaactgggtggtcctggctcagggccactcctgaggctgcagtcagga
 G  K  G  F  S  R  W  I  L  W  V  G  N  S  E  V  A  E  L  G  G  P  G  S  G  P  L  L  R  L  Q  S  G  
tgtccgccagggctgcatcatccgaaggctggactggggccagaggatccacttccaggacagctccgccacacaactgctggcaccggcctcagttcc
 C  P  P  G  L  H  H  P  K  A  G  L  G  P  E  D  P  L  P  G  Q  L  R  H  T  T  A  G  T  G  L  S  S  
ttgctacagggacctctctgcagggctgcttgagtgtcctcctgactcagagcaagtgagagagtgcaagtaggaagccatggtgccttttgcagtcta
 L  L  Q  G  P  L  C  R  A  A  *
gtcctaaaagcggcacaacagccagctgtgggggctcacacctgtaatcccagtacttcgggaggccaaggcaggtggataacttgaggccaggagctcaagaccagcctggccaacatggtgaaaccctgtctctactttaaaaaaaaaaaaagaaaatagctaggcgtggtagtgcacgcttgtaatcccagctacttgggtggttgggacgggaggatcacttgaacccaggagacagaggttgcagtgagctgagattgtgccactggcctccagcctgggcgacagagcaagactctgtctcaaaaaaaaaaaaaaagtggcacattctagtcacaagaaatgaaccatgaagtcccacccacactcgagtggggaatgaagtttcttttgaaaggaacagcatcaaagaatttcaggacatttaaaaaccactgcacatggctagctagatccttggaccatgtaggcttcagcctaactttataaaagttttataaaagttgaatttcctgaagttgactatttttttaaaaatatttcttcttaaaacagtagtgtagttgtgtttttctgagtagcacagtgggatagttctggaatgtcaaccctattagtttaaaatttcaaataaaagctgtggcagtgctcccaaaacaccattgctcatgcaaaaattgtgaagagttaaaattaacgagtagaattctaaagagaaaaaaactgctctttggtgcaagaaataaaaatgtagagaaaagaacaagtctgcaaaaaaaaaaaaaaaaaaaaaaaaaa 


Full-length nucleotide and deduced amino acid sequence of the BORIS B5 isoform (GenBank: DQ778127).  BORIS B5 isoform is expressed from promoter B, contains nine zinc fingers, N-terminus (N24), C-terminus (C24), and 3'UTR belonging to BORIS subfamily 3 (BORIS_sf3. Two pair of primers with corresponding Tagman probes are depicted due to the fact that BORIS B5 isoform was detected as a part of BORIS (sf3) and as an isoform containing Exon b/ Exon 3 splice site (Fig. S4D, Table S2). 


ggcaccagacgcggtgcacgaggcagagccacaagccaaagacggagtgggccgagcattccggccacgccttccgcggagcaaaaggaaccttccactgtgatgtctgcatgttcacctcttctagaatgtcaagttttaatcgtcatatgaaaactcacaccagtgagaagcctcacctgtgtcacctctgcctga
            M  F  T  S  S  R  M  S  S  F  N  R  H  M  K  T  H  T  S  E  K  P  H  L  C  H  L  C  L  
aaaccttccgtacggtcactctgctgcggaaccatgttaacacccacacaggaaccaggccctacaagtgtaacgactgcaacatggcatttgtcacca
K  T  F  R  T  V  T  L  L  R  N  H  V  N  T  H  T  G  T  R  P  Y  K  C  N  D  C  N  M  A  F  V  T  
gtggagaactcgtccgacacaggcgctataaacatactcatgagaaaccctttaaatgttccatgtgcaagtatgccagtgtggaggcaagtaaattga
S  G  E  L  V  R  H  R  R  Y  K  H  T  H  E  K  P  F  K  C  S  M  C  K  Y  A  S  V  E  A  S  K  L  
agcgccatgtccgatcccacactggggagcgcccctttcagtgttgccagtgcagctatgccagcagagatacctacaagctgaaacgccacatgagaa
K  R  H  V  R  S  H  T  G  E  R  P  F  Q  C  C  Q  C  S  Y  A  S  R  D  T  Y  K  L  K  R  H  M  R  
cgcactcaggtgagaagccttacgaatgccacatctgccacacccgcttcacccagagcgggaccatgaaaatacatattctgcagaaacacggcgaaa
T  H  S  G  E  K  P  Y  E  C  H  I  C  H  T  R  F  T  Q  S  G  T  M  K  I  H  I  L  Q  K  H  G  E  
atgtccccaaataccagtgtccccattgtgccaccatcattgcacggaaaagcgacctacgtgtgcatatgcgcaacttgcatgcttacagcgctgcag
N  V  P  K  Y  Q  C  P  H  C  A  T  I  I  A  R  K  S  D  L  R  V  H  M  R  N  L  H  A  Y  S  A  A  
agctgaaatgccgctactgttctgctgtcttccatgaacgctatgccctcattcagcaccagaaaactcataagaatgagaagaggttcaagtgcaaac
E  L  K  C  R  Y  C  S  A  V  F  H  E  R  Y  A  L  I  Q  H  Q  K  T  H  K  N  E  K  R  F  K  C  K  
actgcagttatgcctgcaagcaggaacgtcatatgaccgctcacattcgtacccacactggagagaaaccattcacctgcctttcttgcaataaatgtt
H  C  S  Y  A  C  K  Q  E  R  H  M  T  A  H  I  R  T  H  T  G  E  K  P  F  T  C  L  S  C  N  K  C  
tccgacagaagcaacttctaaacgctcacttcaggaaataccacgatgcaaatttcatcccgactgtttacaaatgctccaagtgtggcaaaggctttt
F  R  Q  K  Q  L  L  N  A  H  F  R  K  Y  H  D  A  N  F  I  P  T  V  Y  K  C  S  K  C  G  K  G  F  
cccgctgggtgttgtattagttatctatttctgtatgacagattacctcaaaatggagtggcttaaaaccacaaacatttatcacctgacagattctct
S  R  W  V  L  Y  *
gggttgggaactcggaagtggctgaactgggtggtcctggctcagggccactcctgaggctgcagtcaggatgtccgccagggctgcatcatccgaagg
ctggactggggccagaggatccacttccaggacagctccgccacacaactgctggcaccggcctcagttccttgctacagggacctctctgcagggctg
cttgagtgtcctcctgactcagagcaagtgagagagtgcaagtaggaagccatggtgccttttgcagtctagtcctaaaagcggcacaacagccagctgtgggggctcacacctgtaatcccagtacttcgggaggccaaggcaggtggataacttgaggccaggagctcaagaccagcctggccaacatggtgaaaccctgtctctactttaaaaaaaaaaaaagaaaatagctaggcgtggtagtgcacgcttgtaatcccagctacttgggtggttgggacgggaggatcacttgaacccaggagacagaggttgcagtgagctgagattgtgccactggcctccagcctgggcgacagagcaagactctgtctcaaaaaaaaaaaaaaagtggcacattctagtcacaagaaatgaaccatgaagtcccacccacactcgagtggggaatgaagtttcttttgaaaggaacagcatcaaagaatttcaggacatttaaaaaccactgcacatggctagctagatccttggaccatgtaggcttcagcctaactttataaaagttttataaaagttgaatttcctgaagttgactatttttttaaaaatatttcttcttaaaacagtagtgtagttgtgtttttctgagtagcacagtgggatagttctggaatgtcaaccctattagtttaaaatttcaaataaaagctgtggcagtgctcccaaaacaccattgctcatgcaaaaattgtgaagagttaaaattaacgagtagaattctaaagagaaaaaaactgctctttggtgcaagaaataaaaatgtagagaaaagaacaagtctgcaaaaaaaaaaaaaaaaaaaaaaaaaa 

     


 Full-length nucleotide and deduced amino acid sequence of the BORIS C6 isoform (GenBank: DQ778121).  BORIS C6 isoform is expressed from promoter C contains five zinc fingers, N-terminus (N258), C-terminus (C90), and 3'UTR belonging to BORIS subfamily 3 (BORIS_sf3). Two pair of primers with corresponding Tagman probes are depicted due to the fact that BORIS C6 isoform was detected as a part of  BORIS (sf3) and as a single isoform (Fig. S4, Table S2). 
    agggtaaagcaggggccctgccaggcctccgagggagtgtgcttggtctggccgagggctgcttggccaagtctgggtgggctcgaggccactaggcccaaagcctgcctggctctgagggtgctaggtctagaaccgtgcacgaggggaatgcctgctcgggcccgaacctcgctgggcgccgggtgtgcactggcccggggcctgcttggacctgaaacttgctaggcccaggatatgcactggccgagagcctgctgggcccaaaccttactaggcccaggatgttcactgactgaaccggctcaggcctaaccttgctaggcccaggatatgcactgggccagagtgtgctcaggcggaaccttgccaggcgcaggatgtgtgctggccctaagcctgctgaggcccaaacctgttcgttctagggttttgtacaaaatcctgctttagcctaaatcctgcttagccttgaccccctcctagacccaagccagatcagcattgttctgaccctactaagtccaaaaccttttgaggccagaccttgtttcaactccaaagcctgctaggttccagcaccccccgcatccctcctcataccacccccttctcccccctatggaaaccgcttgcttatttttcaaacaggccaagtcattatggcagccactgagatctctgtcctttctg
                                                                     M  A  A  T  E  I  S  V  L  S  
agcaattcaccaagatcaaagaactcgagttgatgccggaaaaaggcctgaaggaggaggaaaaagacggagtgtgcagagagaaagaccatcggagcc
E  Q  F  T  K  I  K  E  L  E  L  M  P  E  K  G  L  K  E  E  E  K  D  G  V  C  R  E  K  D  H  R  S  
ctagtgagttggaggccgagcgtacctctggggccttccaggacagcgtcctggaggaagaagtggagctggtgctggccccctcggaggagagcgaga
P  S  E  L  E  A  E  R  T  S  G  A  F  Q  D  S  V  L  E  E  E  V  E  L  V  L  A  P  S  E  E  S  E  
agtacatcctgaccctgcagacggtgcacttcacttctgaagctgtggagttgcaggatatgagcttgctgagcatacagcagcaagaaggggtgcagg
K  Y  I  L  T  L  Q  T  V  H  F  T  S  E  A  V  E  L  Q  D  M  S  L  L  S  I  Q  Q  Q  E  G  V  Q  
tggtggtgcaacagcctggccctgggttgctgtggcttgaggaagggccccggcagagcctgcagcagtgtgtggccattagtatccagcaagagctgt
V  V  V  Q  Q  P  G  P  G  L  L  W  L  E  E  G  P  R  Q  S  L  Q  Q  C  V  A  I  S  I  Q  Q  E  L  
actccccgcaagagatggaggtgttgcagttccacgctctagaggagaatgtgatggtggccagtgaagacagtaagttagcggtgagcctggctgaaa
Y  S  P  Q  E  M  E  V  L  Q  F  H  A  L  E  E  N  V  M  V  A  S  E  D  S  K  L  A  V  S  L  A  E  
ctactggactgatcaagctcgaggaagagcaggagaagaaccagttattggctgaaagaacaaaggagcagctcttttttgtggaaacaatgtcaggag
T  T  G  L  I  K  L  E  E  E  Q  E  K  N  Q  L  L  A  E  R  T  K  E  Q  L  F  F  V  E  T  M  S  G  
atgaaagaagtgacgaaattgttctcacagtttcaaattcaaatgtggaagaacaagaggatcaacctacagctggtcaagcagatgctgaaaaggcca
D  E  R  S  D  E  I  V  L  T  V  S  N  S  N  V  E  E  Q  E  D  Q  P  T  A  G  Q  A  D  A  E  K  A  
aatctacaaaaaatcaaagaaagacaaagggagcaaaaggaaccttccactgtgatgtctgcatgttcacctcttctagaatgtcaagttttaatcgtc
K  S  T  K  N  Q  R  K  T  K  G  A  K  G  T  F  H  C  D  V  C  M  F  T  S  S  R  M  S  S  F  N  R  
atatgaaaactcacaccagtgagaagcctcacctgtgtcacctctgcctgaaaaccttccgtacggtcactctgctgcggaaccatgttaacacccaca
H  M  K  T  H  T  S  E  K  P  H  L  C  H  L  C  L  K  T  F  R  T  V  T  L  L  R  N  H  V  N  T  H  
caggaaccaggccctacaagtgtaacgactgcaacatggcatttgtcaccagtggagaactcgtccgacacaggcgctataaacatactcatgagaaac
T  G  T  R  P  Y  K  C  N  D  C  N  M  A  F  V  T  S  G  E  L  V  R  H  R  R  Y  K  H  T  H  E  K  
cctttaaatgttccatgtgcaagtatgccagtgtggaggaacgtcatatgaccgctcacattcgtacccacactggagagaaaccattcacctgccttt
P  F  K  C  S  M  C  K  Y  A  S  V  E  E  R  H  M  T  A  H  I  R  T  H  T  G  E  K  P  F  T  C  L  
cttgcaataaatgtttccgacagaagcaacttctaaacgctcacttcaggaaataccacgatgcaaatttcatcccgactgtttacaaatgctccaagt
S  C  N  K  C  F  R  Q  K  Q  L  L  N  A  H  F  R  K  Y  H  D  A  N  F  I  P  T  V  Y  K  C  S  K  
gtggcaaaggcttttcccgctggattctctgggttgggaactcggaagtggctgaactgggtggtcctggctcagggccactcctgaggctgcagtcag
C  G  K  G  F  S  R  W  I  L  W  V  G  N  S  E  V  A  E  L  G  G  P  G  S  G  P  L  L  R  L  Q  S  
gatgtccgccagggctgcatcatccgaaggctggactggggccagaggatccacttccaggacagctccgccacacaactgctggcaccggcctcagtt
G  C  P  P  G  L  H  H  P  K  A  G  L  G  P  E  D  P  L  P  G  Q  L  R  H  T  T  A  G  T  G  L  S  
ccttgctacagggacctctctgcagggctgcttgagtgtcctcctgactcagagcaagtgagagagtgcaagtaggaagccatggtgccttttgcagtc
S  L  L  Q  G  P  L  C  R  A  A  *
tagtcctaaaagcggcacaacagccagctgtgggggctcacacctgtaatcccagtacttcgggaggccaaggcaggtggataacttgaggccaggagctcaagaccagcctggccaacatggtgaaaccctgtctctactttaaaaaaaaaaaaagaaaatagctaggcgtggtagtgcacgcttgtaatcccagctacttgggtggttgggacgggaggatcacttgaacccaggagacagaggttgcagtgagctgagattgtgccactggcctccagcctgggcgacagagcaagactctgtctcaaaaaaaaaaaaaaagtggcacattctagtcacaagaaatgaaccatgaagtcccacccacactcgagtggggaatgaagtttcttttgaaaggaacagcatcaaagaatttcaggacatttaaaaaccactgcacatggctagctagatccttggaccatgtaggcttcagcctaactttataaaagttttataaaagttgaatttcctgaagttgactatttttttaaaaatatttcttcttaaaacagtagtgtagttgtgtttttctgagtagcacagtgggatagttctggaatgtcaaccctattagtttaaaatttcaaataaaagctgtggcagtgctcccaaaacaccattgctcatgcaaaaattgtgaagagttaaaattaacgagtagaattctaaagagaaaaaaactgctctttggtgcaagaaataaaaatgtagagaaaagaacaagtctgcaaaaaaaaaaaaaaaaaaaaaaaaaa 


Full-length nucleotide and deduced amino acid sequence of the BORIS B6 isoform (GenBank: DQ778128). BORIS B6 isoform is expressed from promoter B, contains four zinc fingers, N-terminus (N24), C-terminus (C34), and 3'UTR belonging to BORIS subfamily 6 (BORIS_sf6). Two pair of primers with corresponding Tagman probes are depicted due to the fact that BORIS B6 isoform was detected as a part of BORIS (sf6) and as an isoform containing Exon b/ Exon 3 splice site (Fig. S4, Table S2). 


ggcaccagacgcggtgcacgaggcagagcccacaagccaaagacggagtgggccgagcattccggccacgccttccgcggagcaaaaggaaccttccactgtgatgtctgcatgttcacctcttctagaatgtcaagttttaatcgtcatatgaaaactcacaccagtgagaagcctcacctgtgtcacctctgccTG
             M  F  T  S  S  R  M  S  S  F  N  R  H  M  K  T  H  T  S  E  K  P  H  L  C  H  L  C  L aaaaccttccgtacggtcactctgctgcggaaccatgttaacacccacacaggaaccaggccctacaagtgtaacgactgcaacatggcatttgtcACc
 K  T  F  R  T  V  T  L  L  R  N  H  V  N  T  H  T  G  T  R  P  Y  K  C  N  D  C  N  M  A  F  V  T
agtggagaactcgtccgacacaggcgctataaacatactcatgagaaaccctttaaatgttccatgtgcaagtatgccagtgtggaggcaagtaaAttg
 S  G  E  L  V  R  H  R  R  Y  K  H  T  H  E  K  P  F  K  C  S  M  C  K  Y  A  S  V  E  A  S  K  L
aagcgccatgtccgatcccacactggggagcgcccctttcagtgttgccagtgcagctatgccagcagagatacctacaagctgaaacgccacaTGaga
 K  R  H  V  R  S  H  T  G  E  R  P  F  Q  C  C  Q  C  S  Y  A  S  R  D  T  Y  K  L  K  R  H  M  R
acgcactcaggtaagggctctggtgctgaaggcctgatacctacagtgttaactcttaaagcaagctttaaaaaattactttttattggcacaATtaaa
 T  H  S  G  K  G  S  G  A  E  G  L  I  P  T  V  L  T  L  K  A  S  F  K  K  L  L  F  I  G  T  I  K  
gttcaaaggtaaaagtggatttttgcgtgccttcatgataaaagaatcttgatctgtacttttacctttatttagcagtaagagagtctgcaTAgatac
 V  Q  R  *  
tgtgccacaaccccactgtgtggagtaaaacacaaagtatttgcttccgtagatttttcaggtgagaagccttacgaatgccacatctgccacacccgcaaacctgtaatcccagcactttgggaggctgaggcaggaggatcccttgagtccaggagtttgagaccagcctggtcaacatagggagaccctgtctctacgagtaatttaaaaattagctgggcctggtggtgcacacctgtggtcccagctacttgggaggctgaagcaggagaatcacttgaacccaggaggttgaggctgcagtgagccgggattgcgccactacactccagcctgggtgacagagtgagaacctgtctcaaaaaaaaagaaaaagtaaataaaaataaataaaagtcaactccttaattcattcttcaactttaaggcaaaacataaagtgtgctgcttttgtaacagaggtacttgatgtcttgtgttaagaatacatttatgtgtacttcttggttattcgtacagccccatggatgtgaaccaccttgaactcttgcgtagccaccagatgcggggaagtcatgtctctggtccatcatggacacagctgtacttgacataagctgtctgggcttgatttgggagtctcatactaattgggggttgtccggtgagaagggggttgataaaggaggcttggggcaaaaaaaaaaaacactttcagcacaggtggcctttggcaaggatcaggcctggagggggaatcactttgttgtctgcatctcaggtgagaagccttacgaatgccacatctgccacacccgcttcacccagagcgggaccatgaaaatacatattctgcagaaacacggcgaaaatgtccccaaataccagtgtccccattgtgccaccatcattgcacggaaaagcgacctac 


Full-length nucleotide and deduced amino acid sequence of the BORIS B7 isoform (GenBank: DQ778129).  BORIS B7 isoform is expressed from promoter B, contains four zinc fingers, N-terminus (N24), C-terminus (C34), and 3'UTR belonging to BORIS subfamily 6(BORIS_sf6). Two pair of primers with corresponding Tagman probes are depicted due to the fact that BORIS B7 isoform was detected as a part of BORIS (sf6) and as an isoform containing Exon b/ Exon 3 splice site (Fig. S4, Table S2). 


ggcaccagacgcggtgcacgaggcagagcccacaagccaaagacggagtgggccgagcattccggccacgccttccgcggagcaaaaggaaccttccactgtgatgtctgcatgttcacctcttctagaatgtcaagttttaatcgtcatatgaaaactcacaccagtgagaagcctcacctgtgtcacctctgccTG
             M  F  T  S  S  R  M  S  S  F  N  R  H  M  K  T  H  T  S  E  K  P  H  L  C  H  L  C  L aaaaccttccgtacggtcactctgctgcggaaccatgttaacacccacacaggaaccaggccctacaagtgtaacgactgcaacatggcatttgtcACc
 K  T  F  R  T  V  T  L  L  R  N  H  V  N  T  H  T  G  T  R  P  Y  K  C  N  D  C  N  M  A  F  V  T
agtggagaactcgtccgacacaggcgctataaacatactcatgagaaaccctttaaatgttccatgtgcaagtatgccagtgtggaggcaagtaaAttg
 S  G  E  L  V  R  H  R  R  Y  K  H  T  H  E  K  P  F  K  C  S  M  C  K  Y  A  S  V  E  A  S  K  L
aagcgccatgtccgatcccacactggggagcgcccctttcagtgttgccagtgcagctatgccagcagagatacctacaagctgaaacgccacaTGaga
 K  R  H  V  R  S  H  T  G  E  R  P  F  Q  C  C  Q  C  S  Y  A  S  R  D  T  Y  K  L  K  R  H  M  R
acgcactcaggtaagggctctggtgctgaaggcctgatacctacagtgttaactcttaaagcaagctttaaaaaattactttttattggcacaATtaaa
 T  H  S  G  K  G  S  G  A  E  G  L  I  P  T  V  L  T  L  K  A  S  F  K  K  L  L  F  I  G  T  I  K
gttcaaaggtaaaagtggatttttgcgtgccttcatgataaaagaatcttgatctgtacttttacctttatttagcagtaagagagtctgcaTAgatac
 V  Q  R  *  
tgtgccacaaccccactgtgtggagtaaaacacaaagtatttgcttccgtagatttttcaggtgagaagccttacgaatgccacatctgccacacccgcttcacccagagcgggaccatgaaaatacatattctgcagaaacacggcgaaaatgtccccaaataccagtgtccccattgtgccaccatcattgcacggaaaagcgacctac 


 Full-length nucleotide and deduced amino acid sequence of the BORIS C7 isoform (GenBank: DQ778119).  BORIS C7 isoform is expressed from promoter C contains five zinc fingers, N-terminus (N258), C-terminus (C34), and 3'UTR belonging to BORIS subfamily 6 (BORIS_sf6). 

agggtaaagcaggggccctgccaggcctccgagggagtgtgcttggtctggccgagggctgcttggccaagtctgggtgggctcgaggccactaggcccaaagcctgcctggctctgagggtgctaggtctagaaccgtgcacgaggggaatgcctgctcgggcccgaacctcgctgggcgccgggtgtgcactggcccggggcctgcttggacctgaaacttgctaggcccaggatatgcactggccgagagcctgctgggcccaaaccttactaggcccaggatgttcactgactgaaccggctcaggcctaaccttgctaggcccaggatatgcactgggccagagtgtgctcaggcggaaccttgccaggcgcaggatgtgtgctggccctaagcctgctgaggcccaaacctgttcgttctagggttttgtacaaaatcctgctttagcctaaatcctgcttagccttgaccccctcctagacccaagccagatcagcattgttctgaccctactaagtccaaaaccttttgaggccagaccttgtttcaactccaaagcctgctaggttccagcaccccccgcatccctcctcataccacccccttctcccccctatggaaaccgcttgcttatttttcaaacaggccaagtcattatggcagccactgagatctctgtcctttctg
                                                                     M  A  A  T  E  I  S  V  L  S  
agcaattcaccaagatcaaagaactcgagttgatgccggaaaaaggcctgaaggaggaggaaaaagacggagtgtgcagagagaaagaccatcggagcc  
E  Q  F  T  K  I  K  E  L  E  L  M  P  E  K  G  L  K  E  E  E  K  D  G  V  C  R  E  K  D  H  R  S  
ctagtgagttggaggccgagcgtacctctggggccttccaggacagcgtcctggaggaagaagtggagctggtgctggccccctcggaggagagcgaga
P  S  E  L  E  A  E  R  T  S  G  A  F  Q  D  S  V  L  E  E  E  V  E  L  V  L  A  P  S  E  E  S  E  
agtacatcctgaccctgcagacggtgcacttcacttctgaagctgtggagttgcaggatatgagcttgctgagcatacagcagcaagaaggggtgcagg
K  Y  I  L  T  L  Q  T  V  H  F  T  S  E  A  V  E  L  Q  D  M  S  L  L  S  I  Q  Q  Q  E  G  V  Q  
tggtggtgcaacagcctggccctgggttgctgtggcttgaggaagggccccggcagagcctgcagcagtgtgtggccattagtatccagcaagagctgt
V  V  V  Q  Q  P  G  P  G  L  L  W  L  E  E  G  P  R  Q  S  L  Q  Q  C  V  A  I  S  I  Q  Q  E  L  
actccccgcaagagatggaggtgttgcagttccacgctctagaggagaatgtgatggtggccagtgaagacagtaagttagcggtgagcctggctgaaa
Y  S  P  Q  E  M  E  V  L  Q  F  H  A  L  E  E  N  V  M  V  A  S  E  D  S  K  L  A  V  S  L  A  E  
ctGctggactgatcaagctcgaggaagagcaggagaagaaccagttattggctgaaagaacaaaggagcagctcttttttgtggaaacaatgtcaggag
T  A  G  L  I  K  L  E  E  E  Q  E  K  N  Q  L  L  A  E  R  T  K  E  Q  L  F  F  V  E  T  M  S  G  
atgaaagaagtgacgaaattgttctcacagtttcaaattcaaatgtggaagaacaagaggatcaacctacagctggtcaagcagatgctgaaaaggcca
D  E  R  S  D  E  I  V  L  T  V  S  N  S  N  V  E  E  Q  E  D  Q  P  T  A  G  Q  A  D  A  E  K  A  
aatctacaaaaaatcaaagaaagacaaagggagcaaaaggaaccttccactgtgatgtctgcatgttcacctcttctagaatgtcaagttttaatcgtc
K  S  T  K  N  Q  R  K  T  K  G  A  K  G  T  F  H  C  D  V  C  M  F  T  S  S  R  M  S  S  F  N  R  
atatgaaaactcacaccagtgagaagcctcacctgtgtcacctctgcctgaaaaccttccgtacggtcactctgctgcggaaccatgttaacacccaca
H  M  K  T  H  T  S  E  K  P  H  L  C  H  L  C  L  K  T  F  R  T  V  T  L  L  R  N  H  V  N  T  H  
caggaaccaggccctacaagtgtaacgactgcaacatggcatttgtcaccagtggagaactcgtccgacacaggcgctataaacatactcatgagaaac
T  G  T  R  P  Y  K  C  N  D  C  N  M  A  F  V  T  S  G  E  L  V  R  H  R  R  Y  K  H  T  H  E  K  
cctttaaatgttccatgtgcaagtatgccagtgtggaggcaagtaaattgaagcgccatgtccgatcccacactggggagcgcccctttcagtgttgcc
P  F  K  C  S  M  C  K  Y  A  S  V  E  A  S  K  L  K  R  H  V  R  S  H  T  G  E  R  P  F  Q  C  C  
agtgcagctatgccagcagagatacctacaagctgaaacgccacatgagaacgcactcaggtaagggctctggtgctgaaggcctgatacctacagtgt
Q  C  S  Y  A  S  R  D  T  Y  K  L  K  R  H  M  R  T  H  S  G  K  G  S  G  A  E  G  L  I  P  T  V  
taactcttaaagcaagctttaaaaaattactttttattggcacaATtaaagttcaaaggtaaaagtggatttttgcgtgccttcatgataaaagaatct
L  T  L  K  A  S  F  K  K  L  L  F  I  G  T  I  K  V  Q  R  *  
tgatctgtacttttacctttatttagcagtaagagagtctgcaTAgatactgtgccacaaccccactgtgtggagtaaaacacaaagtatttgcttccgtagatttttcaggtatttaaaactcaactcctggccaggcatggtggctcaaacctgtaatcccagcactttgggaggctgaggcaggaggatcccttgagtccaggagtttgagaccagcctggtcaacatagggagaccctgtctctacgagtaatttaaaaattagctgggcctggtggtgcacacctgtggtcccagctacttgggaggctgaagcaggagaatcacttgaacccaggaggttgaggctgcagtgagccgggattgcgccactacactccagcctgggtgacagagtgagaacctgtctcaaaaaaaaagaaaaagtaaataaaaataaataaaagtcaactccttaattcattcttcaactttaaggcaaaacataaagtgtgctgcttttgtaacagaggtacttgatgtcttgtgttaagaatacatttatgtgtacttcttggttattcgtacagccccatggatgtgaaccaccttgaactcttgcgtagccaccagatgcggggaagtcatgtctctggtccatcatggacacagctgtacttgacataagctgtctgggcttgatttgggagtctcatactaattgggggttgtccggtgagaagggggttgataaaggaggcttggggcaaaaaaaaaaaacactttcagcacaggtggcctttggcaaggatcaggcctggagggggaatcactttgttgtctgcatctcaggtgagaagccttacgaatgccacatctgccacacccgcttcacccagagcgggaccatgaaaatacatattctgcagaaacacggcgaaaatgtccccaaataccagtgtccccattgtgccaccatcattgcacggaaaagcgacctac 


 Full-length nucleotide and deduced amino acid sequence of the BORIS C9 isoform (GenBank: DQ778120).   BORIS C9 isoform is expressed from promoter C, contains five zinc fingers, N-terminus (N258), C-terminus (C34), and 3'UTR belonging to BORIS subfamily 6 (BORIS_sf6). 


agggtaaagcaggggccctgccaggcctccgagggagtgtgcttggtctggccgagggctgcttggccaagtctgggtgggctcgaggccactaggcccaaagcctgcctggctctgagggtgctaggtctagaaccgtgcacgaggggaatgcctgctcgggcccgaacctcgctgggcgccgggtgtgcactggcccggggcctgcttggacctgaaacttgctaggcccaggatatgcactggccgagagcctgctgggcccaaaccttactaggcccaggatgttcactgactgaaccggctcaggcctaaccttgctaggcccaggatatgcactgggccagagtgtgctcaggcggaaccttgccaggcgcaggatgtgtgctggccctaagcctgctgaggcccaaacctgttcgttctagggttttgtacaaaatcctgctttagcctaaatcctgcttagccttgaccccctcctagacccaagccagatcagcattgttctgaccctactaagtccaaaaccttttgaggccagaccttgtttcaactccaaagcctgctaggttccagcaccccccgcatccctcctcataccacccccttctcccccctatggaaaccgcttgcttatttttcaaacaggccaagtcattatggcagccactgagatctctgtcctttctg
                                                                     M  A  A  T  E  I  S  V  L  S  
agcaattcaccaagatcaaagaactcgagttgatgccggaaaaaggcctgaaggaggaggaaaaagacggagtgtgcagagagaaagaccatcggagcc  
E  Q  F  T  K  I  K  E  L  E  L  M  P  E  K  G  L  K  E  E  E  K  D  G  V  C  R  E  K  D  H  R  S  
ctagtgagttggaggccgagcgtacctctggggccttccaggacagcgtcctggaggaagaagtggagctggtgctggccccctcggaggagagcgaga
P  S  E  L  E  A  E  R  T  S  G  A  F  Q  D  S  V  L  E  E  E  V  E  L  V  L  A  P  S  E  E  S  E  
agtacatcctgaccctgcagacggtgcacttcacttctgaagctgtggagttgcaggatatgagcttgctgagcatacagcagcaagaaggggtgcagg
K  Y  I  L  T  L  Q  T  V  H  F  T  S  E  A  V  E  L  Q  D  M  S  L  L  S  I  Q  Q  Q  E  G  V  Q  
tggtggtgcaacagcctggccctgggttgctgtggcttgaggaagggccccggcagagcctgcagcagtgtgtggccattagtatccagcaagagctgt
V  V  V  Q  Q  P  G  P  G  L  L  W  L  E  E  G  P  R  Q  S  L  Q  Q  C  V  A  I  S  I  Q  Q  E  L  
actccccgcaagagatggaggtgttgcagttccacgctctagaggagaatgtgatggtggccagtgaagacagtaagttagcggtgagcctggctgaaa
Y  S  P  Q  E  M  E  V  L  Q  F  H  A  L  E  E  N  V  M  V  A  S  E  D  S  K  L  A  V  S  L  A  E  
ctgctggactgatcaagctcgaggaagagcaggagaagaaccagttattggctgaaagaacaaaggagcagctcttttttgtggaaacaatgtcaggag
T  A  G  L  I  K  L  E  E  E  Q  E  K  N  Q  L  L  A  E  R  T  K  E  Q  L  F  F  V  E  T  M  S  G  
atgaaagaagtgacgaaattgttctcacagtttcaaattcaaatgtggaagaacaagaggatcaacctacagctggtcaagcagatgctgaaaaggcca
D  E  R  S  D  E  I  V  L  T  V  S  N  S  N  V  E  E  Q  E  D  Q  P  T  A  G  Q  A  D  A  E  K  A  
aatctacaaaaaatcaaagaaagacaaagggagcaaaaggaaccttccactgtgatgtctgcatgttcacctcttctagaatgtcaagttttaatcgtc
K  S  T  K  N  Q  R  K  T  K  G  A  K  G  T  F  H  C  D  V  C  M  F  T  S  S  R  M  S  S  F  N  R  
atatgaaaactcacaccagtgagaagcctcacctgtgtcacctctgcctgaaaaccttccgtacggtcactctgctgcggaaccatgttaacacccaca
H  M  K  T  H  T  S  E  K  P  H  L  C  H  L  C  L  K  T  F  R  T  V  T  L  L  R  N  H  V  N  T  H  
caggaaccaggccctacaagtgtaacgactgcaacatggcatttgtcaccagtggagaactcgtccgacacaggcgctataaacatactcatgagaaac
T  G  T  R  P  Y  K  C  N  D  C  N  M  A  F  V  T  S  G  E  L  V  R  H  R  R  Y  K  H  T  H  E  K  
cctttaaatgttccatgtgcaagtatgccagtgtggaggcaagtaaattgaagcgccatgtccgatcccacactggggagcgcccctttcagtgttgcc
P  F  K  C  S  M  C  K  Y  A  S  V  E  A  S  K  L  K  R  H  V  R  S  H  T  G  E  R  P  F  Q  C  C  
agtgcagctatgccagcagagatacctacaagctgaaacgccacatgagaacgcactcaggtaagggctctggtgctgaaggcctgatacctacagtgt
Q  C  S  Y  A  S  R  D  T  Y  K  L  K  R  H  M  R  T  H  S  G  K  G  S  G  A  E  G  L  I  P  T  V  
taactcttaaagcaagctttaaaaaattactttttattggcacaATtaaagttcaaaggtaaaagtggatttttgcgtgccttcatgataaaagaatct
L  T  L  K  A  S  F  K  K  L  L  F  I  G  T  I  K  V  Q  R  *  
tgatctgtacttttacctttatttagcagtaagagagtctgcaTAgatactgtgccacaaccccactgtgtggagtaaaacacaaagtatttgcttccgtagatttttcaggtatagatttttcaggtgagaagccttacgaatgccacatctgccacacccgcttcacccagagcgggaccatgaaaatacatattctgcagaaacacggcgaaaatgtccccaaataccagtgtccccattgtgccaccatcattgcacggaaaagcgacctac 
