# Supplementary material for: The Structural Complexity of the Human BORIS Gene in Gametogenesis and Cancer
Source: PLoS One. 2010 Nov 8;5(11):e13872. doi: 10.1371/journal.pone.0013872 (PMC2975627; doi:10.1371/journal.pone.0013872)
Supplement: File S2 — BORIS isoforms B0, C2, A5, C3, B1 and C7/C9 are the dominant representatives of the six BORIS subfamilies. (0.07 MB RTF) [file pone.0013872.s002.rtf]

File S2.
BORIS isoforms B0, C2, A5, C3, B1 and C7/C9 are the dominant representatives of the six BORIS subfamilies. 
Analyzing BORIS isoform expression by RT-PCR and Northern blotting in testes and the K562 cell line (Fig. S4B,C), it was shown that not all isoforms of the six BORIS subfamilies were expressed equally within the subfamily. Each BORIS subfamily had at least one isoform that was expressed at higher levels than the others, while some were expressed at barely detectable levels. To identify the dominant isoform(s) for each subfamily, we performed a series of experiments by combining qRT-PCR, Northern blotting and RT-PCR analyses of alternative transcripts in RNA samples from either adult testis or the K562 cell line. 
The BORIS subfamily one (BORIS sf1) consists of 5 members – BORIS (B0), A1, A2, A3, and C1. A3 has a unique splice site that was used to design a Taqman probe and primers to measure its contribution to sf1 expression. According to RT-PCR and qRT-PCR assays with absolute quantification approach, A3 represents about 10% and 20% of sf1 expression in testes and K562 cells, respectively (Fig. S4,C(1), data not shown). The remaining four transcripts of sf1 are expressed from the three alternative promoters of BORIS and encode the same protein. Thus, we concluded that BORIS (B0) itself, expressed from three alternative promoters, would be the dominant protein isoform of BORIS sf1 (Fig. S4F). 
Two methods, qRT-PCR and Northern assays, showed that BORIS C2 is the dominant form of sf2 (Fig.S4B,C(2)), because RT-PCR yielded much stronger PCR product with a forward primer from the promoter C, then from promoter A, in accordance with that, Northern blotting detected one strong RNA band of 2000 b in size, which corresponds to BORIS C2 isoform. 
The sf3 Taqman probe detects 5 BORIS isoforms (Fig. 2A). The expression levels of four of them (A6, B4, B5, and C6) were quantified by absolute qRT-PCR, showing that less than 31% of the total amount of BORIS transcripts are detected by BORIS sf3 probe in the RNA isolated from K562 cell line (Fig.S4D). It is likely that the remaining 69% are contributed by A5 that does not contain any unique sequences or splice sites to be specifically detected by qRT-PCR. Indeed, upon combining qRT-PCR with RT-PCR and Northern blot data (Fig. S4B,D), where single dominant band was detected, BORIS isoform A5 emerges as the dominant transcript detected by the sf3 Taqman probe in both testis and K562 cell line. 
Analysis of BORIS sf4, which consists of at least six members (C3, B2, B3, C4, C5, C8), showed one dominant band by Northern blotting and several RT-PCR bands with the dominant upper transcript corresponding to the C3 isoform (Fig. S4B,C(4)). In parallel, qRT-PCR assays with 5 Taqman probes was used to estimate that the total amount of single transcripts produced by B2, B3, C4, C5 and C8 comprised about 21% of the total contribution of these isoform transcripts to sf4. Therefore, the remaining 79% are contributed by the C3 isoform, which makes C3 as a dominant form of sf4 (Fig.S4E).    
BORIS sf5 is represented by the single B1 isoform, as confirmed by RT-PCR and Northern blotting. Aside from the single expected 2kb band corresponding to BORIS B1 form, there were multiple transcripts over 4k in length on Northern blotting, detected by sf5 probe (Fig.S4B). They are likely to be nonspecific cross-hybridization RNAs, as they were also present in BORIS-negative kidney samples.
BORIS sf6 consists of four members: B6, B7, C7 and C9. B6 and B7 have alternative 3'UTRs, but encoded single BORIS isoproteins as well as C7 and C9 (Fig. 2A, File S1). The Taqman probe designed to detect all expressed sf6 transcripts yielded 2000 and 1300 transcripts per 50 ng of total RNA extracted from testis and K562 cells, respectively (Fig. 4A and Fig. 5B). Relative amounts of B6 and B7 isoforms, that have Exb – Ex3 splice site, were quantified by qRT-PCR with a Taqman probe specific for this splice site. The total amount of transcripts containing the Exb-Ex3 splice site (B2, B5) is less than 140 and 80 transcripts per 50 ng of total RNA extracted from testis and K562 cells, respectively (Fig. S4D,E, data not shown). Taking into account that Northern blotting detected a single band over 2kb with the sf6 probe, we suggested that C7 and C9 transcripts, which encode one protein are the dominant isoforms of sf6.  
Thus, based on the expression analysis of the individual BORIS isoforms within their own subfamilies, we were able to identify the dominant subspecies of the six BORIS mRNA subfamilies (Fig. S4F,G). Most BORIS isoforms were undetectable by Northern blots and were close to the limits of detection by regular RT-PCR. These low abundance isoforms could be targets of Nonsense-Mediated mRNA decay (NMD), an endogenous cell surveillance mechanism. Transcripts become subject to NMD, if the stop codon is located less than 50 bp upstream from the last exon-exon junction. According to this 50-nucleotide rule, three out of 23 BORIS isoforms - C8, A6 and B5 - are likely targets of the NMD pathway and should be under-represented in total RNA, due to RNA degradation by the exosome complex of NMD pathway. Indeed, according to qRT-PCR analyses, these isoforms were expressed at low levels (Fig. S4D, E). The lower abundance of NMD-inducing transcripts compared to that of full-length stable RNA products could be another indication of their targeting by the NMD pathway. Thus, ten more isoforms (B2, B3, B4, B6, B7, C4, C5, C6, A3, A4) could be targeted by NMD based on their low expression within particular subfamilies (Fig. S4). The remaining 10 isoforms encode six BORIS isoproteins, which we identified as the main representatives of the six BORIS subfamilies (Fig. S4F, G): BORIS B1 encoded isoBORIS protein 1 (sf1) (Table S3); C3 encoded isoBORIS protein 2 (sf2); BORIS B0, A1, A2, C1 encoded isoBORIS protein 3 (sf1); A5 encoded isoBORIS protein 4 (sf3); C2 encoded isoBORIS protein 13 (sf2); C7 and C9 encoded isoBORIS protein 11 (sf6) (Table S3, Fig. S4F, G). All six main BORIS isoform proteins have long N-258 termini, 5 of 6 isoproteins possess more than 5 ZFs in ZF domain (Fig S4G), suggesting that they are capable to bind some DNA targets, such as the CST promoter, and activate their transcription. 
Although BORIS isoforms with low abundance could be a result of stochastic errors by splicing machinery and thus have no biological roles, we believe this is unlikely. All 23 BORIS isoforms have features of canonically spliced transcripts: the classic exon–intron junctions are utilized to produce multiple isoforms (Table S4), and the 3'UTRs are relatively long and contain the typical polyA signal, classically located 20-30 nucleotides from the beginning of the polyA tail, a feature of mRNAs that do mature (File S1). This suggests that even while some BORIS isoforms expressed at very low levels they might still play important roles in spermatogenesis, oogenesis and cancer progression. 
