# Supplementary figures and images for: The Structural Complexity of the Human BORIS Gene in Gametogenesis and Cancer
Source: PLoS One. 2010 Nov 8;5(11):e13872. doi: 10.1371/journal.pone.0013872 (PMC2975627; doi:10.1371/journal.pone.0013872)

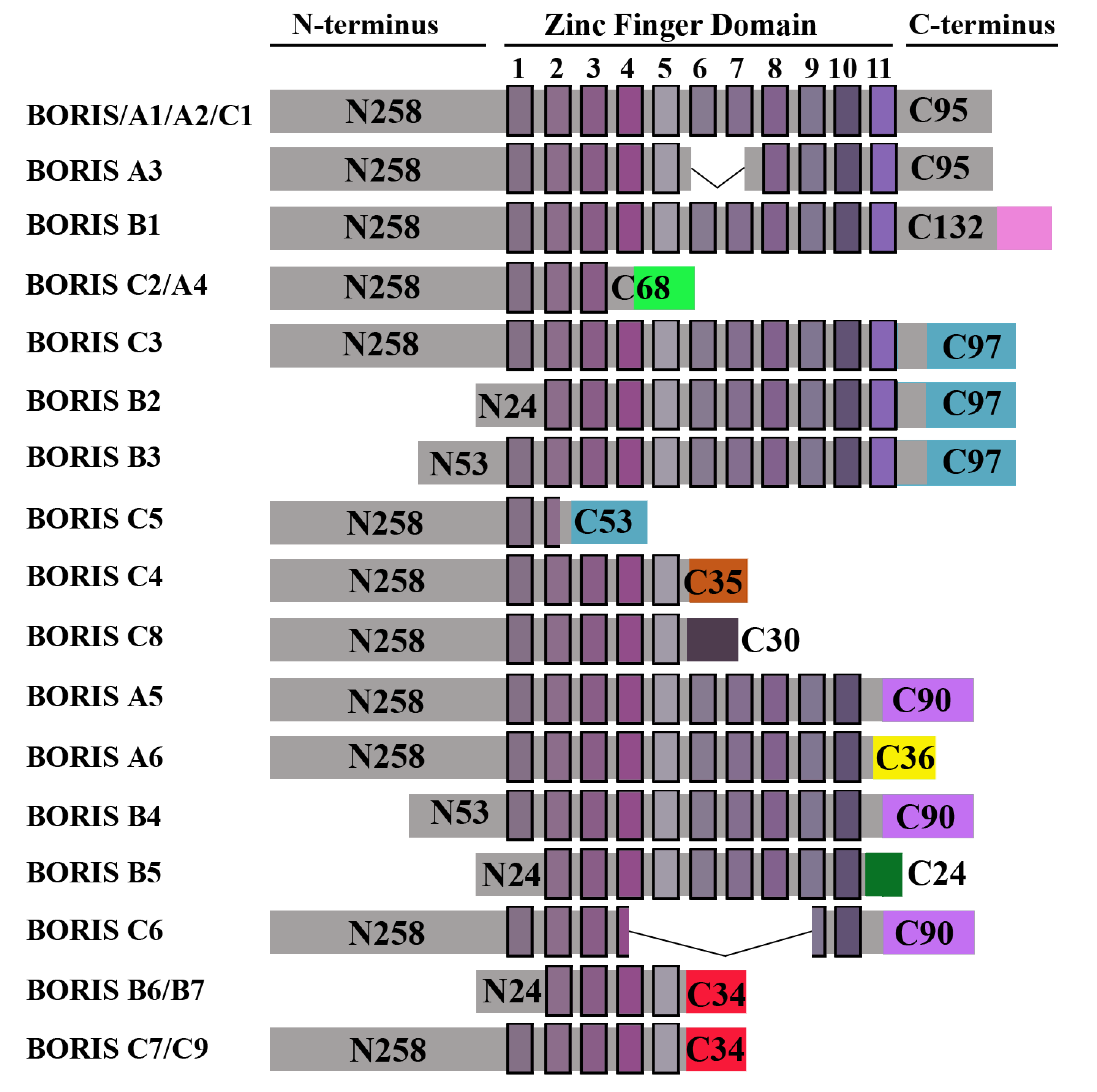

Supplement: Figure S1 — The 23 alternatively spliced RNAs are predicted 17 protein isoforms with three alternative N-termini and eleven alternative C-termini. Unique alternative C-termini labeled by different colors. (0.70 MB TIF) [file pone.0013872.s003.tif]

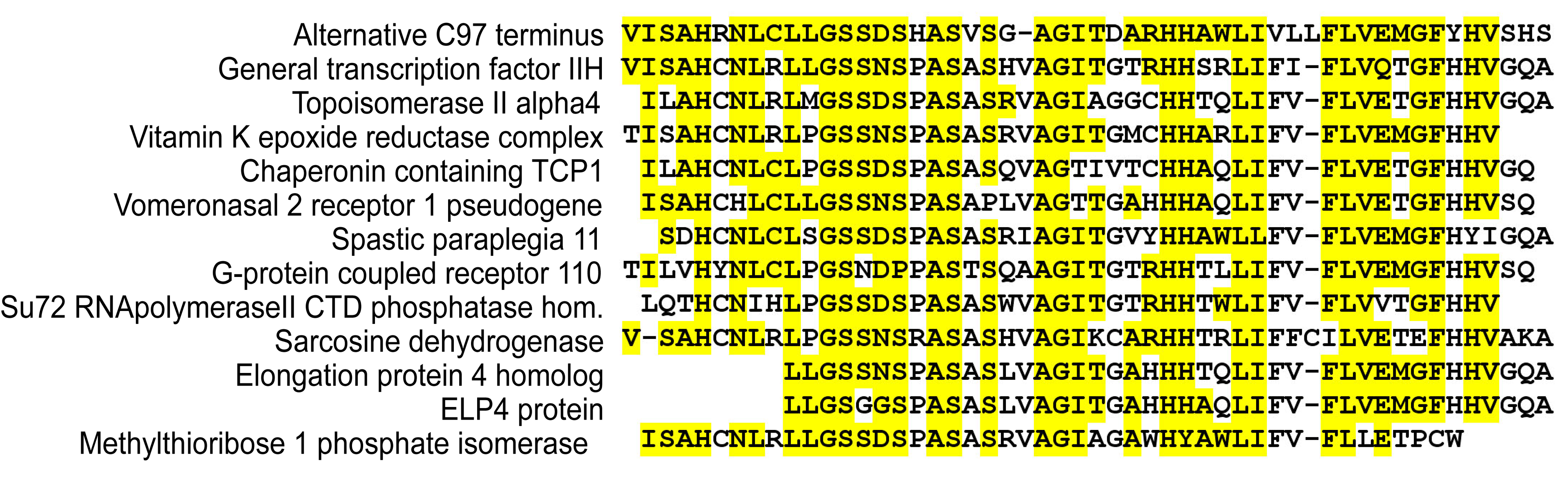

Supplement: Figure S2 — Alignment of alternative C-terminus C97, present in BORIS C3, B2, and B3 isoforms, demonstrated a similarity from 52% to 72% with more than 100 human proteins, some of them are involved in transcription or translation processes. The proteins sharing homology with C97 include: transmembrane protein 50B (TMEM50B) (56%), general transcription factor IIH (GTF2H1) (61% identity), seven transmembrane helix receptor (GPR110) (58%), INO80 complex homolog 1 (INO80) (59%), putative calcium-sensing receptor-like 1 (64%), syntaxin 8 (STX8) (72%), vitamin K epoxide reductase complex (VCORC1) (65%), topoisomerase II alpha-4 (TOP2A) (56%), elongation protein 4 homolog (ELP4) (62%), SSU72 RNA polymerase II CTD phosphatase homolog (SSU72) (58%), sarcosine dehydrogenase (SARDH) (53%), translation initiation factor eIF-2B subunit alpha/beta/delta-like protein (MRI1) (60%) and many more. (3.88 MB TIF) [file pone.0013872.s004.tif]

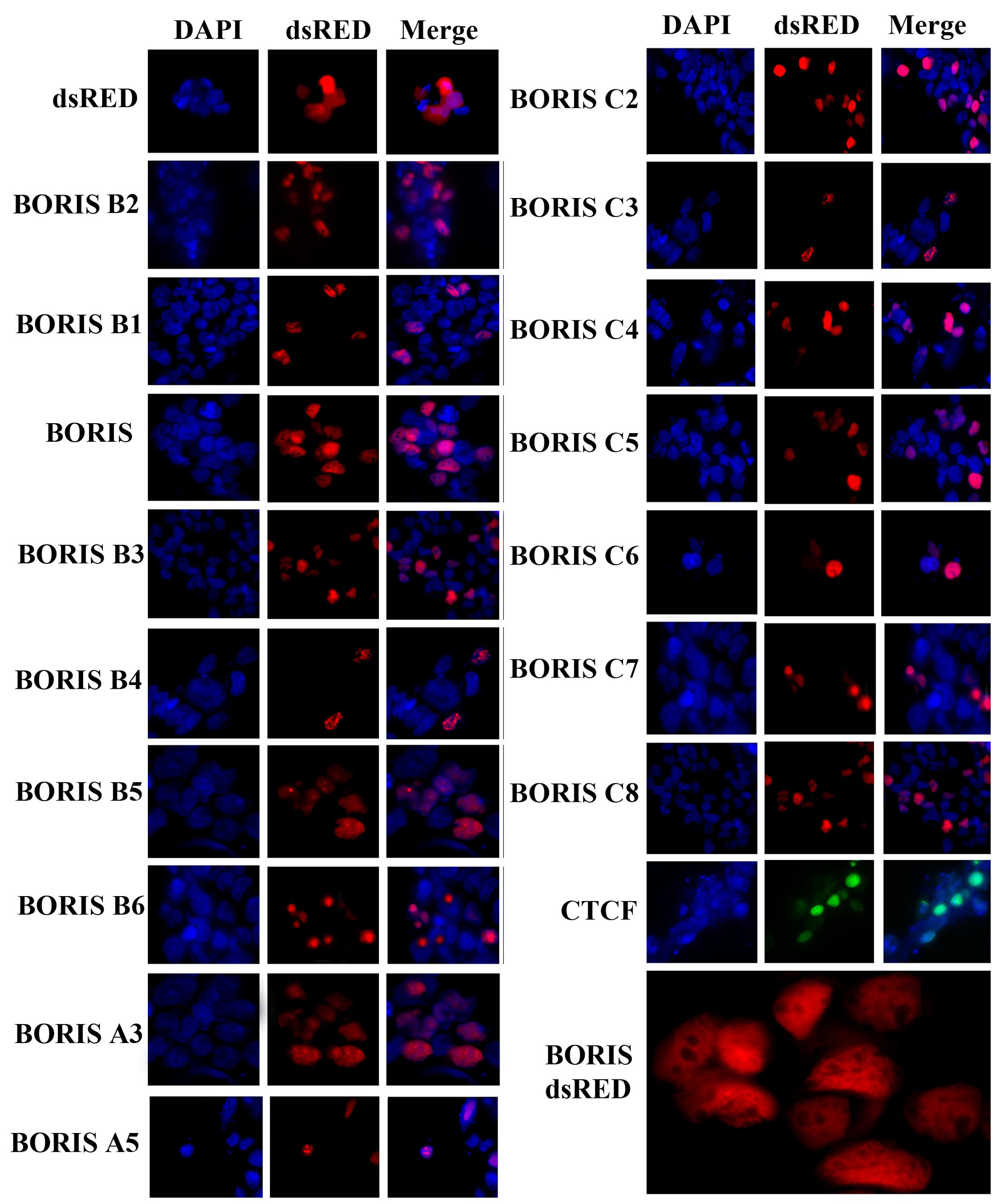

Supplement: Figure S3 — All BORIS isoproteins are located in nuclei. HEK293T cells were transiently transfected with either dsRRED empty vector (EV-dsRED) or with BORIS isoforms and CTCF fused to dsRRED or GFP at N-terminus, respectively. RFP and GFP fluorescence were analyzed by microscopy. dsRED protein (Empty Vector (EV)-dsRED) was served as a marker for cytoplasmic location. Cells were also stained with DAPI to visualize nuclear DNA. At the bottom of right column the high magnification image of BORIS B0 transfected cells are shown to demonstrate the punctual pattern of BORIS nuclear location. (2.85 MB TIF) [file pone.0013872.s005.tif]

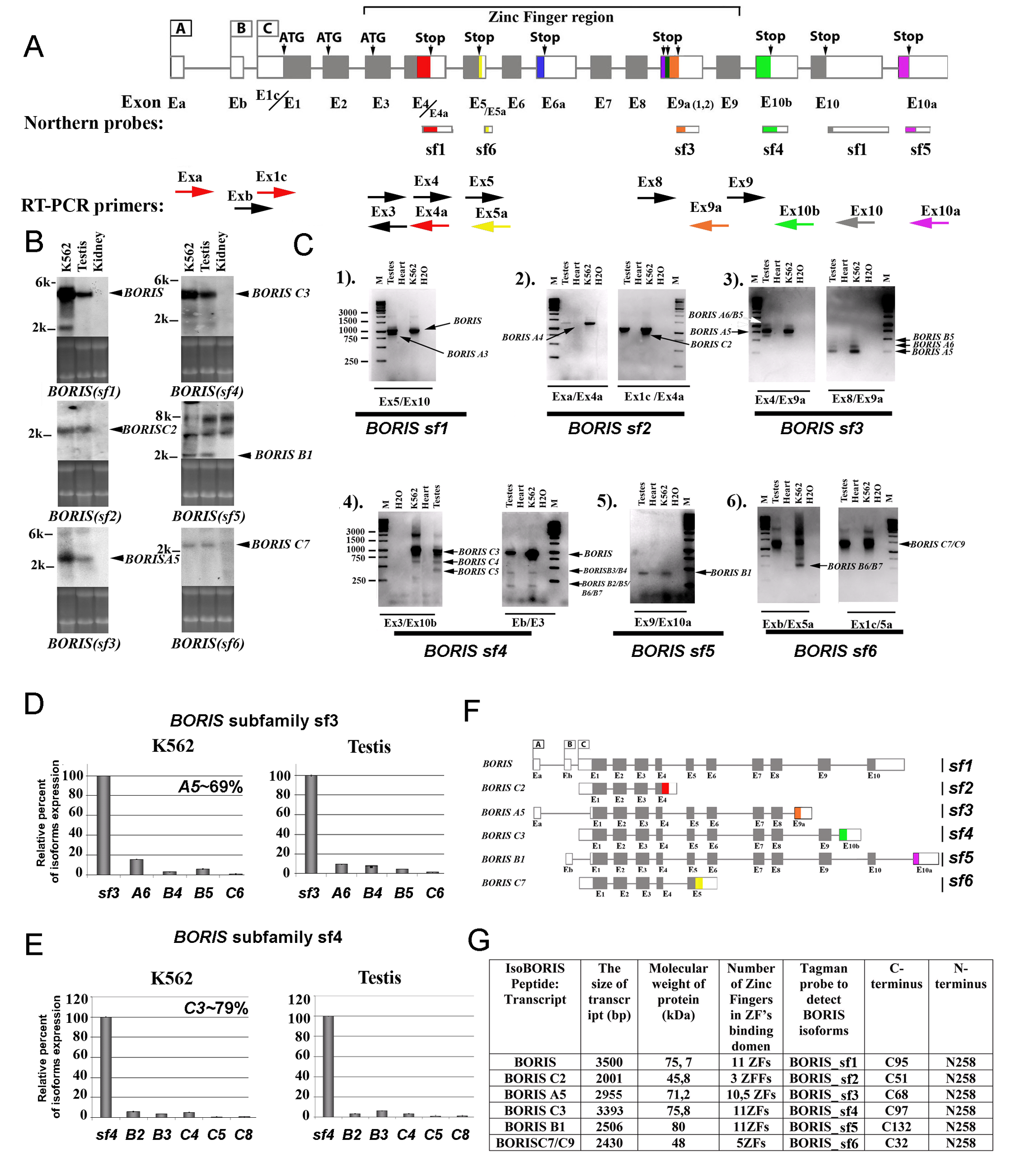

Supplement: Figure S4 — BORIS isoforms subfamilies are comprised by distinctly expressed individual isoforms. (A). A schematic illustration of BORIS promoters and exons usage for the expression of 23 mRNA isoforms. The rectangular boxes at the bottom denote the locations and sizes of probes that were used for Northern blotting. Arrows correspond to primers that were used in RT-PCR assays. The colors of boxes and arrows correspond to the unique coding alternative sequences as in Figure 2. (B). Northern blotting analysis of BORIS isoforms expression in the K562 cell line, adult testis and kidney. The top 6 gels correspond to six internally 32P-labeled probes for 6 BORIS subfamilies. The bottom gels are loading controls based on ribosomal RNA. The six labeled probes were designed to detect all isoforms of 6 BORIS subfamilies. Sequences of primers used to generate Northern probes are shown in Table S1. The dominant transcripts are indicated by arrows with a corresponding name of BORIS isoform. The sizes of RNA transcripts are shown on the right side of the membrane. (C). RT-PCR assay to simultaneously detect multiple isoforms within six BORIS subfamilies by using one or two set of primers. Agarose gels are shown for every BORIS subfamily (BORIS _sf1-sf6). The PCR products were generated from adult testis and the K562 cDNAs by nested RT-PCR. H2O and RNA extracted from heart were used as negative controls for BORIS expression. The primers that were used to amplify single or multiple transcripts within one subfamily are named accordingly to their mapping to BORIS exons and shown at the bottom of each gel. These primers are also shown in panel A; the sequence of primers is shown in Table S1. PCR products with the size of expected alternative transcripts are indicated by arrows with the name of corresponding isoform. Subpanel 1). To simultaneously amplify BORIS B0 and A3 transcripts within sf1, we used the forward primer from exon 5 (Ex5) and the reverse primer from exon 10 (Ex10). Subpanel 2). For [file pone.0013872.s006.tif]
