# Supplementary material for: The Structural Complexity of the Human BORIS Gene in Gametogenesis and Cancer
Source: PLoS One. 2010 Nov 8;5(11):e13872. doi: 10.1371/journal.pone.0013872 (PMC2975627; doi:10.1371/journal.pone.0013872)
Supplement: Table S1 — Table of primers used in RT-PCR and PCR assays. (0.11 MB DOC) [file pone.0013872.s007.doc]

| **Gene/Isoform/Sequence** | **Forward primer 5’-3’** | **Reverse primer 5’-3’** | |
| --- | --- | --- | --- |
| **Primers to amplify BORIS alternative transcripts by using RLM-RACE approach** | | | |
| **a). A first round of PCR with total RNA from testis and K562, processed with GeneRacer PCR Kit, as template (3’RACE**) | | | |
| Transcripts from Promoter A | gggtctcccagcgccccctgcgggg | | GeneRacer3’: gctgtcaacgatacgctacgtaacg |
| Transcripts from Promoter B | gtgcacgaggcagagcccacaagc | | GeneRacer3’: gctgtcaacgatacgctacgtaacg |
| Transcripts from Promoter C | gcgcaggatgtgtgctggccctaagcctgctg | | GeneRacer3’: gctgtcaacgatacgctacgtaacg |
| **b). A second round of PCR with one mkl of the first round of PCR as template (3’RACE)** | | | |
| Transcripts from Promoter A | ctcctcccttcctcatccacttcaacc | | GeneRacerNest.3’: cgctacgtaacggcatgacagtg |
| Transcripts from Promoter B | gacggagtgggccgagcattccgg | | GeneRacerNest.3’: cgctacgtaacggcatgacagtg |
| Transcripts from Promoter C | ctgcttagccttgaccccctcctagacccaagcc | | GeneRacerNest.3’: cgctacgtaacggcatgacagtg |
| **Primers to amplify the entire Zinc Finger region of *BORIS* and *CTCF*** | | | |
| CTCF ZF 1^st^ PCR round | gtggttggtaatatgaagcctccaaag | | gtttgggctggttggttctgccagg |
| CTCF ZF 2^nd^ PCR round | gaagacattccagtgtgagctttgcag | | gtcgtcctctccgcttcttggcgggtg |
| BORIS ZF 1^st^ PCR round | gaggatcaacctacagctggtcaag | | gcttccttccatcccttcgcagcttccttc |
| BORIS ZF 2^nd^ PCR round | gaaaaggccaaatctacaaaaaatcaaag | | ctgaccctttgtggcttccttcaggatg |
| **Primers to amplify the ORFs for 17 BORIS isoproteins** | | | |
| ORF_BORISB1(isoprotein1)  1^st^ PCR round | 5’-cgatgttccagattacgctgcagccac  tgagatctctgtcctttctgag-3’ | | 5’-Cgcctactccctcttcgccttctg  ccttgattgtaagtttc-3’ |
| ORF_BORISB1(isoprotein1)  2^nd^ PCR round | cccatgggAattcAtgtacccatacgat  gttccagattacgctgcagccactg | | Ggctagcgcggccgctcaccatgtgacgc  gcctactccctcttcgccttctgc |
| ORF_BORISC3 (isoprotein2) 1^st^ PCR round | cgatgttccagattacgctgcagccac  tgagatctctgtcctttctgag | | Ctactaaaaataatagaacaattagc  caggcgtggtggcgggcatc |
| ORF_BORIS C3(isoprotein2)  2^nd^ PCR round | cccatgggAattcAtgtacccatacgat  gttccagattacgctgcagccactg | | Ggctagcgcggccgctcaggagtgagagac  atggtaaaaccccatctctactaaaaataa |
| ORF_BORIS (isoprotein3)  1^st^ PCR round | cgatgttccagattacgctgcagccac  tgagatctctgtcctttctgag | | Cgtgttgaggagcatttcacaggtcacg  ccttcatcc |
| ORF_BORIS (isoprotein3)  2^nd^ PCR round | cccatgggAattcAtgtacccatacgat  gttccagattacgctgcagccactg | | Ggctagcgcggccgctcacttatccatc  gtgttgaggagcatttcacag |
| ORF_BORISA5 (isoprotein4) 1^st^ PCR round | cgatgttccagattacgctgcagccac  tgagatctctgtcctttctgag | | Gagaggtccctgtagcaaggaactgag  gccggtgccagcagttgtgtggc |
| ORF_BORISA5(isoprotein4)  2^nd^ PCR round | cccatgggAattcAtgtacccatacgat  gttccagattacgctgcagccactg | | Ggctagcgcggccgctcaagcagccctg  cagagaggtccctgtagcaaggaac |
| ORF_BORISA3 (isoprotein5) 1^st^ PCR round | cgatgttccagattacgctgcagccac  tgagatctctgtcctttctgag | | Cgtgttgaggagcatttcacaggtcacg  ccttcatcc |
| ORF_BORISA3(isoprotein5)  2^nd^ PCR round | cccatgggAattcAtgtacccatacgat  gttccagattacgctgcagccactg | | Ggctagcgcggccgctcacttatccatc  gtgttgaggagcatttcacag |
| ORF_BORISA6 (isoprotein6) 1^st^ PCR round | cgatgttccagattacgctgcagccac  tgagatctctgtcctttctgag | | Cctgcagagaggtccctgtagcaag  gaactgaggccggtgccagc |
| ORF_BORISA6(isoprotein6)  2^nd^ PCR round | cccatgggAattcAtgtacccatacgat  gttccagattacgctgcagccactg | | Ggctagcgcggccgctcaagcagcc  ctgcagagaggtcc |
| ORF_BORISB3 (isoprotein7) 1^st^ PCR round | gttccagattacgctTCAGGAGATGAA  AGAAGTGACGaAATTG | | Ctactaaaaataatagaacaattagc  caggcgtggtggcgggcatc |
| ORF_BORISB3(isoprotein7)  2^nd^ PCR round | cccatgggAattcAtgtacccatacgat  gttccagattacgctTCAGGAG | | Ggctagcgcggccgctcaggagtgagagac  atggtaaaaccccatctctactaaaaataa |
| ORF_BORISC8 (isoprotein8) 1^st^ PCR round | cgatgttccagattacgctgcagccac  tgagatctctgtcctttctgag | | Caggaatcgtaggtcgcttttccgtg  caatgatggtggc |
| ORF_BORISC8(isoprotein8)  2^nd^ PCR round | cccatgggAattcAtgtacccatacgat  gttccagattacgctgcagccactg | | Ggctagcgcggccgctcatggaaaagg  gaggcccaggaatcgtaggtcgcttttc |
| ORF_BORISC4 (isoprotein9) 1^st^ PCR round | cgatgttccagattacgctgcagccac  tgagatctctgtcctttctgag | | Gagtgagagacatggtaaaacccc  atctctactaaaaataatagaac |
| ORF_BORISC4(isoprotein9)  2^nd^ PCR round | cccatgggAattcAtgtacccatacgat  gttccagattacgctgcagccactg | | Ggctagcgcggccgctcaggagtgag  agacatggtaaaaccccatc |
| ORF_BORISB4(isoprotein10) 1^st^ PCR round | gttccagattacgctTCAGGAGATGAA  AGAAGTGACGaAATTG | | Gagaggtccctgtagcaaggaactgag  gccggtgccagcagttgtgtggc |
| ORF_BORISB4(isoprotein10)  2^nd^ PCR round | cccatgggAattcAtgtacccatacgat  gttccagattacgctTCAGGAG | | Ggctagcgcggccgctcaagcagccctg  cagagaggtccctgtagcaaggaac |
| ORF_BORISC7(isoprotein11) 1^st^ PCR round | cgatgttccagattacgctgcagccac  tgagatctctgtcctttctgag | | Gtgccaataaaaagtaattttttaaag  cttgctttaagag |
| ORF_BORISC7(isoprotein11)  2^nd^ PCR round | cccatgggAattcAtgtacccatacgat  gttccagattacgctgcagccactg | | GgctagcgcggccgcttacctttgaacttTA  attgtgccaataaaaagtaattttttaaaG |
| ORF_BORISB2(isoprotein12) 1^st^ PCR round | cagattacgctttcacctcttctagaat  gtcaagttttaatc | | Ctactaaaaataatagaacaattagc  caggcgtggtggcgggcatc |
| ORF_BORISB2(isoprotein12)  2^nd^ PCR round | cccatgggAattcAtgtacccatacga  tgttccagattacgctttcac | | Ggctagcgcggccgctcaggagtgagagac  atggtaaaaccccatctctactaaaaataa |
| ORF_BORISC2(isoprotein13) 1^st^ PCR round | cgatgttccagattacgctgcagccac  tgagatctctgtcctttctgag | | Catattatttcctgcataaattttatatg  aataataaaaagcctg |
| ORF_BORISC2(isoprotein13)  2^nd^ PCR round | cccatgggAattcAtgtacccatacgat  gttccagattacgctgcagccactg | | Ggctagcgcggccgctcataaaagagaatgcatattatttcctgcataaattttatatg |
| ORF_BORISC5(isoprotein14) 1^st^ PCR round | cgatgttccagattacgctgcagccac  tgagatctctgtcctttctgag | | Gagagacatggtaaaaccccatctctactaaaaataatagaacaattag |
| ORF_BORISC5(isoprotein14)  2^nd^ PCR round | cccatgggAattcAtgtacccatacgat  gttccagattacgctgcagccactg | | Ggctagcgcggccgctcaggagtgaga  gacatggtaaaaccccatc |
| ORF_BORISB5(isoprotein15) 1^st^ PCR round | cagattacgctttcacctcttctagaat  gtcaagttttaatc | | Ctgcagagaggtccctgtagcaaggaac  tgaggccggtgccagcag |
| ORF_BORISB5(isoprotein15)  2^nd^ PCR round | cccatgggAattcAtgtacccatacga  tgttccagattacgctttcac | | Ggctagcgcggccgctcaagcagccctg  cagagaggtccc |
| ORF_BORISC6(isoprotein16) 1^st^ PCR round | cgatgttccagattacgctgcagccac  tgagatctctgtcctttctgag | | Gagaggtccctgtagcaaggaactgag  gccggtgccagcagttgtgtggc |
| ORF_BORISC6(isoprotein16)  2^nd^ PCR round | cccatgggAattcAtgtacccatacgat  gttccagattacgctgcagccactg | | Ggctagcgcggccgctcaagcagccctg  cagagaggtccctgtagcaaggaac |
| ORF_BORISB6(isoprotein17) 1^st^ PCR round | cagattacgctttcacctcttctagaat  gtcaagttttaatc | | Ctttaattgtgccaataaaaagtaatttttta  aagcttgctttaagagttaacac |
| ORF_BORISB6(isoprotein17)  2^nd^ PCR round | cccatgggAattcAtgtacccatacga  tgttccagattacgctttcac | | Ggctagcgcggccgcttacctttgaactttaatt  gtgccaataaaaagtaattttttaaagc |
| **Primers to identify the dominant isoform(s) for each BORIS subfamilies (Figure S26)** | | | |
| Ex1a 1^st^ PCR round | ggtctcccagcgccccctgcggggc | |  |
| Ex1a 2^nd^ PCR round | cctcccttcctcatccacttcaacc | |  |
| Ex1c 1^st^ PCR round | gaggccagaccttgtttcaactccaaag | |  |
| Ex1c 2^nd^ PCR round | cctgctaggttccagcaccccccgcatc | |  |
| Exb 1^st^ PCR round | ggctgcagcacgcggtgcacgaggc | |  |
| Exb 2^nd^ PCR round | cccacaagccaaagacggagtgggc | |  |
| Ex3 1^st^ PCR round | ctgtgtcacctctgcctgaaaaccttcc | |  |
| Ex3 2^nd^ PCR round | gtacggtcactctgctgcggaaccatg | |  |
| Ex4 1^st^ PCR round | cgacacaggcgctataaacatactcatg | |  |
| Ex4 2^nd^ PCR round | gttccatgtgcaagtatgccagtgtgg | |  |
| Ex5 1^st^ PCR round | catgtccgatcccacactggggagcgccc | |  |
| Ex5 2^nd^ PCR round | gcagctatgccagcagagatacctacaag | |  |
| Ex8 1^st^ PCR round | ctcacattcgtacccacactggag | |  |
| Ex8 2^nd^ PCR round | gctcacttcaggaaataccacgatg | |  |
| Ex9c 1^st^ PCR round | ctgaaggaagccacaaagggtcagaag | |  |
| Ex9c 2^nd^ PCR round | gctgcgaagggatggaaggaagccgcgaac | |  |
| Ex3 1^st^ PCR round |  | | gcttctcactggtgtgagttttcatatg |
| Ex3 2^nd^ PCR round |  | | gattaaaacttgacattctagaagag |
| Ex4a 1^st^ PCR round |  | | cagcctctactaagatgccatgaag |
| Ex4a 2^nd^ PCR round |  | | gttagttacacttggagtaacttgtac |
| Ex5a 1^st^ PCR round |  | | ggttgtggcacagtatctatgcagactctc |
| Ex5a 2^nd^ PCR round |  | | ctgtaggtatcaggccttcagcaccagag |
| Ex9a 1^st^ PCR round |  | | gcagccctggcggacatcctgactgcagc |
| Ex9a 2^nd^ PCR round |  | | caggagtggccctgagccaggaccacc |
| Ex10b 1^st^ PCR round |  | | gtggcgggcatctgtaatcccagctcc |
| Ex10b 2^nd^ PCR round |  | | gactgaggcatgagaatcacttgaac |
| Ex10 1^st^ PCR round |  | | ggcagtgaacatgcaacctgactctctctc |
| Ex10 2^nd^ PCR round |  | | catcgtgttgaggagcatttcacac |
| Ex10a 1^st^ PCR round |  | | gcctactccctcttcgccttctgccttg |
| Ex10a 2^nd^ PCR round |  | | gtttcctgaggcctccccagaagccag |
| **Primers for amplification of probes used in Northern blot and in situ Hybridization** | | | |
| BORIS (sf1) | CGATGGATAAGTGAGAGGGATTCGG | | GCCTCTGTATCCAGATAGAAATG |
| BORIS (sf2) | CAAGTATGCCAGTGTGGAGGTAAAG | | AACCACAGATGGTTTATTTGTAATTTG |
| BORIS (sf3) | GCAAAGGCTTTTCCCGCTGGATTCTC | | GAAGTACTGGGATTACAGGTGTGAGC |
| BORIS (sf4) | GAAGGGATGGAAGGAAGCCGCGAAC | | GGTGGATGGAGGGAGACAAGAAGAC |
| BORIS (sf5) | GGCGTGACCTGTGAAATGCTCCTCAACAC | | CAAACATATTTATGTTATCTGTTTTTG |
| BORIS (sf6) | caagctgaaacgccacatgagaacg | | ccaccatgcctggccaggagttgag |
| **Primers for amplification of probes used for EMSA** | | | |
| Mouse *CST*_promoter F | cgggtcggggaggacgctgtcctgg | | cagagtactttccggaactccgag |
| Human *CST_*promoter F | GAACTGTCCTGGGCGGGGCCAGGTG | | CCAGCAGCCCCACTCACCAAGGTAC |
| *H19-IGF2* 6^th^CTCFsite | gggggctcttgcatagcacatggg | | gtgacccgggacgtttccacgggc |
| **Primers for amplification of probes used in RNase Protection Assay** | | | |
| BORIS (sf1) | gtggatgaaggcgtgacctgtgaaatg | | CTCACTTATCCATCGTGTTGAGGAG |
| BORIS (sf2) | catgagaaaccctttaaatgttc | | GCCTGTTTGTAACAGATTCTACTG |
| BORIS (sf3) | caaatgctccaagtgtggcaaag | | CCCAGTCCAGCCTTCGGATGATGC |
| BORIS (sf4) | gagcccctgtgcctggcctgatggcac | | CATGTTCTAACTTGTCTGAG |
| BORIS (sf6) | cagagatacctacaagctgaaacgccac | | GCACGCAAAAATCCACTTTTACCTTTGAAC |
| BORIS C6 | ggagaactcgtccgacacaggcg | | CATTTATTGCAAGAAAGGCAGGTGAATG |
| GAPDH | ccacgacgtactcagcgccag | | gtttacatgttccaatatgattccac |
