# Supplementary material for: The Structural Complexity of the Human BORIS Gene in Gametogenesis and Cancer
Source: PLoS One. 2010 Nov 8;5(11):e13872. doi: 10.1371/journal.pone.0013872 (PMC2975627; doi:10.1371/journal.pone.0013872)
Supplement: Table S2 — The primers and the Tagman probes used in qRT-PCR. (0.04 MB DOC) [file pone.0013872.s008.doc]

| Probe | Transcript | Primer sequence  Forward(For):5’-3’; Reverse (Rev):5’-3’ | Tagman probe sequence  *6FAM- MGBNFQ* |
| --- | --- | --- | --- |
| **Probe BORIS (sf1)** | ***BORIS, BORIS A1, A2, A3, C1*** | **For: GCCACAAAGGGTCAGAAGGA**  **Rev: TCCTCAGCAGCAGCTTCGT** | **TGCGAAGGGATGGAA** |
| **Probe BORIS (sf2)** | ***BORIS A4, C2*** | **For: CAAGTATGCCAGTGTGGAGGTAAA**  **Rev: CAGCCTCTACTAAGATGCCATGAA** | **CCATTCTTGGACTTGAA*G*** |
| **Probe BORIS (sf3)** | ***BORIS A5, A6, B4, B5, C6*** | **For: AGTGCAAGTAGGAAGCCATGGT**  **Rev: GGCTGTTGTGCCGCTTTT** | **CCTTTTGCAGTCTAGTCC** |
| **Probe BORIS (sf4)** | ***BORIS C3, B2, B3, C4, C5, C8*** | **For: CCTGTGCCTGGCCTGATG**  **Rev: GGGAGACAAGAAGACCCAGTTC** | **GTTTTGTGGATCTCAGTGTT** |
| **Probe BORIS (sf5)** | ***BORIS B1*** | **For: GAAGGCGTGACCTGTGAAATG**  **Rev: TCCTGTACAGCCTGCGGAAT** | **TCCTCAACACGATGGAT** |
| **Probe BORIS (sf6)** | ***BORIS B6, B7, C7, C9*** | **For: TTTATTTAGCAGTAAGAGAGTCTGCATAGAT**  **Rev: CTACGGAAGCAAATACTTTGTGTTTT** | **CTGTGCCACAACCCCACTGTGTGG** |
| **BORIS A3** | ***BORIS A3*** | **For: AGCTGAAACGCCACATGAGA**  **Rev: AAGCATGCAAGTTGCGCATA** | **CGCACTCAGGTGTGC -** |
| **BORIS C8** | ***BORIS C8*** | **For: CCCATTGTGCCACCATCAT**  **Rev: AAAGGGAGGCCCAGGAATC** | **CACGGAAAAGCGACCTA** |
| **Exb/Ex2** | ***BORIS B3, B4*** | **For: AGTGGGCCGAGCATTCC**  **Rev: CTTTCAGCCAATAACTGGTTCTTCT** | **CCACGCCTTCCGC** |
| **Exb/Ex3** | ***BORIS B2, B5, B6, B7*** | **For: GGAGTGGGCCGAGCATT**  **Rev: AGACATCACAGTGGAAGGTTCCTT** | **CCACGCCTTCCGC** |
| **BORIS C6** | ***BORIS C6*** | **For: CCTTTAAATGTTCCATGTGCAAGT**  **Rev: CGAATGTGAGCGGTCATATGA** | **TGCCAGTGTGGAGGAA** |
| **Ex8/Ex9a(2)** | ***BORIS B5*** | **For: CCAAGTGTGGCAAAGGCTTT**  **Rev:** **CTCCATTTTGAGGTAATCTGTCATACA** | **CCCGCTGGGTGTTG** |
| **Ex8/Ex9a(1)** | ***BORIS A6*** | **For: CGCTGGATTACCTCAAAATGG**  **Rev:** **CCCAGAGAATCTGTCAGGTGATAA** | **TGGCTTAAAACCACAAAC** |
| **BORIS C4** | ***BORIS C4*** | **For: AGCTGAAACGCCACATGAGA**  **Rev:** **ATTTCCTGAAGTGAGCGTTTAGAAG** | **CGCACTCAGAAGCA** |
| **BORIS C5** | ***BORIS C5*** | **For: CTCACACCAGTGAGAAGCCTCAC**  **Rev:** **CGGAGACTGAGGCATGAGAATC** | **CACCTCTGCCTCCTG** |
| **hMAGE A1** | ***MAGE A1*** | **For: TGAGGGACGGCGTAGAGTTC**  **Rev: TGAAAACCTTGCCTCCTCACA** | **CCGAAGGAACCTGACC** |
| **p53** | ***p53*** | **From Applied Biosystem, Foster City, CA (Assay ID Details Hs00153349_m1)** | |
| **hGAPDH** | ***GAPDH*** | **From Applied Biosystem, Foster City, CA (Assay ID Details Hs99999905_m1)** | |
| **hCTCF** | ***CTCF*** | **From Applied Biosystem, Foster City, CA (Assay ID Details:** [**Hs00902011_g1**](https://products.appliedbiosystems.com:443/ab/en/US/adirect/ab?cmd=ABAssayDetailDisplay&assayID=Hs00902011_g1&Fs=y&adv_phrase3=EXACT&adv_phrase2=EXACT&adv_phrase1=EXACT&assayType=GE&catID=601267&SearchRequest.Common.SortSpec=score+desc&searchValue=ctcf&searchBy=all&adv_kw_filter3=ALL&srchType=keyword&adv_kw_filter2=ALL&adv_kw_filter1=ALL&inventoried=*&adv_query_text3=&searchType=keyword&adv_query_text2=&adv_query_text1=&adv_boolean3=AND&displayAdvSearchResults=null&SearchRequest.Common.ResultsPerPage=25&adv_boolean2=AND&adv_boolean1=AND&chkBatchQueryText=false&kwfilter=ALL&SearchRequest.Common.PageNumber=2&isSL=null&msgType=ABGEKeywordResults)**)** | |
