# Supplementary material for: The Structural Complexity of the Human BORIS Gene in Gametogenesis and Cancer
Source: PLoS One. 2010 Nov 8;5(11):e13872. doi: 10.1371/journal.pone.0013872 (PMC2975627; doi:10.1371/journal.pone.0013872)
Supplement: Table S3 — The 23 alternatively spliced BORIS mRNAs are predicted 17 isoproteins. Table 3 summarizes the information about BORIS alternative forms, including the size of alternative transcripts in nucleotides, the size of isoproteins in kDA, the calculated isoelectric point of each protein, the number of ZF per isoform, the alternative N- and C-termini and the GenBank accession number for each BORIS isoform. (0.05 MB DOC) [file pone.0013872.s009.doc]

| **BORIS isopeptide: Transcript** | **The size of transcript (bp)** | **Molecular weight of protein (kDa)** | **Isoelectric point (*pI*)** | **Number of Zinc Fingers in binding domen** | **N-termini** | **C-termini** | **GenBank accession number** |
| --- | --- | --- | --- | --- | --- | --- | --- |
| **BORIS isoprotein 1:**  ***BORIS B1*** | **2506** | **80** | **8.82** | **11 ZFs** | **N258** | **C132** | **DQ778111** |
| **BORIS isoprotein 2:**  ***BORIS C3*** | **3393** | **75,8** | **8.85** | **11 ZFs** | **N258** | **C97** | **DQ778115** |
| **BORIS isoprotein 3:**  ***BORIS***  ***BORIS A1***  ***BORIS A2***  ***BORIS C1*** | **3500**  **3601**  **3701**  **4073** | **75, 7** | **8.58** | **11 ZFs** | **N258** | **C95** | **AF336042**  **DQ778108**  **DQ778109**  **DQ778110** |
| **BORIS isoprotein 4:**  ***BORIS A5*** | **2955** | **71** | **8.51** | **10 ZFs** | **N258** | **C90** | **DQ778122** |
| **BORIS isoprotein 5:**  ***BORIS A3*** | **3897** | **70** | **8.42** | **9 ZFs** | **N258** | **C95** | **DQ778112** |
| **BORIS isoprotein 6:**  ***BORIS A6*** | **3002** | **66** | **8.65** | **10 ZFs** | **N258** | **C36** | **DQ778123** |
| **BORIS isoprotein 16:**  ***BORIS C6*** | **2995** | **54,2** | **6.11** | **5 ZFs** | **N258** | **C90** | **DQ778121** |
| **BORIS isoprotein 7:**  ***BORIS B3*** | **2267** | **52,6** | **9.75** | **11 ZFs** | **N53** | **C97** | **DQ778125** |
| **BORIS isoprotein 8:**  ***BORIS C8*** | **4030** | **51,4** | **6.35** | **6 ZFs** | **N258** | **C30** | **DQ778118** |
| **BORIS isoprotein 9:**  ***BORIS C4*** | **2999** | **49** | **6.02** | **5 ZFs** | **N258** | **C35** | **DQ778116** |
| **BORIS isoprotein 10:**  ***BORIS B4*** | **2300** | **48** | **9.59** | **10 ZFs** | **N53** | **C90** | **DQ778126** |
| **BORIS isoprotein 11:**  ***BORIS C7***  ***BORIS C9*** | **>2964**  **>2241** | **48** | **6.31** | **5 ZFs** | **N258** | **C34** | **DQ778119**  **DQ778120** |
| **BORIS isoprotein 12:**  ***BORIS B2*** | **2056** | **46,5** | **9.91** | **10 ZFs** | **N24** | **C97** | **DQ778124** |
| **BORIS isoprotein 13:**  ***BORIS A4***  ***BORIS C2*** | **1529**  **2001** | **45,8** | **5.48** | **3 ZFs** | **N258** | **C68** | **DQ778113**  **DQ778114** |
| **BORIS isoprotein 14:**  ***BORIS C5*** | **2394** | **37** | **4.77** | **1 ZF** | **N258** | **C53** | **DQ778117** |
| **BORIS isoprotein 15:**  ***BORIS B5*** | **2173** | **35** | **9.85** | **9ZFs** | **N24** | **C24** | **DQ778127** |
| **BORIS isoprotein 17:**  ***BORIS B6***  ***BORIS B7*** | **>1627**  **>902** | **19** | **10.26** | **4 ZFs** | **N24** | **C34** | **DQ778128**  **DQ778129** |
