# Supplementary material for: The Structural Complexity of the Human BORIS Gene in Gametogenesis and Cancer
Source: PLoS One. 2010 Nov 8;5(11):e13872. doi: 10.1371/journal.pone.0013872 (PMC2975627; doi:10.1371/journal.pone.0013872)
Supplement: Table S4 — Exon-Intron Junctions of the BORIS gene. The major spliceosome splices introns containing GU at the 5′ splice site and AG at the 3′ splice site. (0.05 MB DOC) [file pone.0013872.s010.doc]

| **BORIS isoforms** | **Exon** | **Splice Donor site (SD)** | **Splice Acceptor site (SA)** |
| --- | --- | --- | --- |
| *A1, A4, A5, A6* | Ea-E1 | CAAG**//gt**attt | aac**ag**//GCCA |
| *A2* | Ea-Eb | CAAG**//gt**attt | ccc**ag**//GTCC |
| *BORIS, B1* | Eb-E1 | CGCG//**gt**gagg | aac**ag**//GCCA |
| *B3, B4* | Eb-E2 | CGCG//**gt**gagg | att**ag**//CTCG |
| *B2, B5, B6, B7* | Eb-E3 | CGCG//**gt**gagg | tat**ag**//GAGC |
| *BORIS, A1, A2, A3, A4, A5, A6, B1, C1, C2, C3, C4, C5, C6, C7, C8, C9* | E1-E2 | CAAG//**gt**acag | att**ag**//CTCG |
| *BORIS, A1, A2, A3, A4, A5, A6, B1, B3, B4, C1, C2, C3, C4, C5, C6, C7, C8, C9* | E2-E3 | AAGG//**gt**aggc | tat**ag**//GAGC |
| *All isoforms, except C5* | E3-E4 | ACAG//**gt**aaaa | tac**ag**//GAAC |
| *C5* | E3-E10 | ACCT//ctgcc | aacct//CTGC |
| *BORIS, A1, A2, A3, A5, A6, B1, B2, B3, B4, B5, B6, B7, C1, C3, C4, C7, C8, C9* | E4-E5 | GGAG//**gt**aaag | att**ag**//GCAA |
| *C6* | E4-E8 | GGAG//**gt**aaag | gac**ag**//ATTA |
| *BORIS, A1, A2, A5, A6, B1, B2, B3, B4, B5, C1, C3, C8* | E5-E6 | TCAG//**gt**aagg | ctc**ag**//GTGA |
| *A3* | E5-E7 | TCAG//**gt**aagg | ttt**ag**//GTGT |
| *B7, C9* | E5a-E6 | TCAG//**gt**attt | ctc**ag**//GTGA |
| *C4* | E5-E8 | TCAG//**gt**aagg | gac**ag**//AAGC |
| *BORIS, A1, A2, A5, A6, B1, B2, B3, B4, B5, C1, C3,* | E6-E7 | CTAC//**gt**gagt | ttt**ag**//GTGT |
| *C8* | E6-E6a | CTAC//**gt**gagt | ttc**ag**//GATT |
| *BORIS, A1, A2, A3, A5, A6, B1, B2, B3, B4, B5, C1, C3, C8* | E7-E8 | GCAG//**gt**attg | ctt**ag**//GAAC |
| *BORIS, A1, A2, A3, B1, B2, B3, C1, C3, C4, C8* | E8-E9 | CTGG//**gt**aagc | gac**ag**//ATTA |
| *A5, B4, C6* | E8-E9a | CTGG//**gt**aagc | gac**ag**//ATTC |
| *A6* | E8-E9a(1) | CTGG//**gt**aagc | gac**ag**//ATTA |
| *B5* | E8-E9a(2) | CTGG//**gt**aagc | ttt**ag**//GTGT |
| *BORIS, A1, A2, A3, C1* | E9_E10 | GACG//**gt**actg | tca**ag**//AAG |
| *B2, B3, C3, C4, C8* | E9-E10b | GACG//**gt**actg | tcc**ag**//GTGT |
| *B1* | E10-E10a | ATAA//**gt**gaga | cat**ag**//TTCC |
