# Supplementary material for: The Structural Complexity of the Human BORIS Gene in Gametogenesis and Cancer
Source: PLoS One. 2010 Nov 8;5(11):e13872. doi: 10.1371/journal.pone.0013872 (PMC2975627; doi:10.1371/journal.pone.0013872)
Supplement: Table S5 — Table of BORIS Expressed Sequence Tags (EST) in Gene Bank. (0.03 MB DOC) [file pone.0013872.s011.doc]

| **GeneBank Number (EST)** | **BORIS (IsoForm)** | **Type of tissue** | **Sequence’s size** |
| --- | --- | --- | --- |
| **AW243377** | ***BORIS A4/C2, (BORIS* sf2*)*** | **2 pooled wilms' tumors, one primary and one metastatic to brain** | **522bp** |
| **BU170289** | ***BORIS* sf1** | **retinoblastoma** | **887bp** |
| **BF209606** | ***BORIS* sf1** | **chronic myelogenous leukemia** | **775bp** |
| **BG687495** | ***BORIS* sf1** | **mucoepidermoid carcinoma** | **872bp** |
| **BF185295** | ***BORIS* sf1** | **chronic myelogenous leukemia** | **841bp** |
| **BG527694** | ***BORIS* sf1** | **mucoepidermoid carcinoma** | **711bp** |
| **BU156125** | ***5’UTR_*Promoter A** | **retinoblastoma** | **1148bp** |
| **AL705921** | ***BORIS C8*** | **Library: 686 (synonym: hlcc3)** | **842bp** |
